# Social and financial incentives for overcoming a collective action problem

**DOI:** 10.1016/j.jdeveco.2023.103072

**Published:** 2023-05

**Authors:** M. Mehrab Bakhtiar, Raymond P. Guiteras, James Levinsohn, Ahmed Mushfiq Mobarak

**Affiliations:** a International Food Policy Research Institute, House 10A, Road 35, Gulshan 2, Dhaka 1212, Bangladesh; b North Carolina State University, Nelson Hall, Campus Box 8109, Raleigh NC, 27695, USA; c Yale University, 165 Whitney Ave., New Haven, CT 06520, USA; d Deakin University, 221 Burwood Highway, Burwood 3125, Australia

**Keywords:** Development, Environment, Sanitation, Collective action problems

## Abstract

Addressing public health externalities often requires community-level collective action. Due to social norms, each person’s sanitation investment decisions may depend on the decisions of neighbors. We report on a cluster randomized controlled trial conducted with 19,000 households in rural Bangladesh where we grouped neighboring households and introduced (either financial or social recognition) rewards with a joint liability component for the group, or asked each group member to make a private or public pledge to maintain a hygienic latrine. The group financial reward has the strongest impact in the short term (3 months), inducing a 7.5–12.5 percentage point increase in hygienic latrine ownership, but this effect dissipates in the medium term (15 months). In contrast, the public commitment induced a 4.2–6.3 percentage point increase in hygienic latrine ownership in the short term, but this effect persists in the medium term. Non-financial social recognition or a private pledge has no detectable effect on sanitation investments.

## Highlights

• Randomized trial on hygienic latrine adoption in rural Bangladesh.
• Tests financial incentives, social recognition and joint public commitments.
• Financial reward has the largest short term impact, but effect fades.
• Public commitment has a smaller but more persistent effect.
• Social recognition has no detectable effect.

## 1 Introduction

One billion people, or about 15% of the world’s population, currently practice open defecation (OD) in spite of the existence of simple, affordable pour flush latrines that effectively confine fecal matter in sealed pits (WHO and UNICEF, 2017). Open defecation spreads bacterial, viral, and parasitic infections, and has been identified as a leading cause of child stunting (Spears, 2013, Chambers and Von Medeazza, 2013, Augsburg and Rodríguez-Lesmes, 2018) and infant death (Hathi et al., 2017). Diarrheal diseases kill nearly one million people per year (Prüss-Ustün et al., 2014), and cause nearly 20% of deaths of children under five in low income countries (Mara et al., 2010).

Since these pathogens are communicable, a large portion of the health gains from a household’s use of a hygienic latrine likely accrue to other households in the community (Fuller et al., 2016, Andrés et al., 2017). This creates a divergence between the incidence of benefits and costs, and with it, a classic collective action problem — while it may be in all households’ interests collectively for all households to use and maintain hygienic latrines, any individual household may not find these behaviors privately optimal. With strong institutions, regulation mandating adoption and enforcing use can solve this problem. However, in the absence of such institutions, other tools are required.

In this paper, we test several such tools designed to overcome this collective action problem in rural Bangladesh, a setting where social and financial incentives to encourage sanitation adoption and maintenance are a promising alternative to command-and-control approaches. The interventions were designed to help groups of households overcome collective action failures impeding investment and maintenance of hygienic latrines. All participating households are grouped with 15–20 neighbors who jointly participate in monthly meetings for 3 months with a health worker from a well-known NGO to discuss sanitation, OD and disease risk. On top of this common treatment, we randomize four additional treatments and study their effects.

The first treatment, a group “monetary reward”, is a slight variation on the standard public finance policy prescription: a subsidy for a well-maintained hygienic latrine. The non-standard component is an element of joint liability: households receive the reward only if both that household’s latrine is hygienic *and* a certain share of all households in the group maintain a hygienic latrine. Given the financial sustainability concerns about such payments, we substitute a recognition certificate from the local government instead of money as our second treatment, and call this a “recognition reward”. The same element of joint group liability is also present for this treatment, and only the form of the reward is changed. This treatment is more akin to certifications like open-defection-free (ODF) status sometimes conferred by governments to encourage investments in improved sanitation.

Our third treatment, “public commitment”, explores whether a simple verbal coordination device between neighbors can sustain a cooperative equilibrium (Schelling, 1960). In public commitment groups, all households in the group are asked to make a joint public (but non-binding) commitment in front of each other, stating that they will try to address the OD issue in their neighborhood by using and maintaining hygienic latrines. This public commitment could be operating through two mechanisms. First, the act of making a commitment is an “implementation intention” that can itself spur action (Gollwitzer and Brandstätter, 1997). Second, the fact that this commitment is made in public in front of and with others who are making the same commitment simultaneously can help coordinate action. Our fourth treatment, “private commitment”, was designed to separate these two mechanisms. In this arm, all group members are asked to make the same pledge as those in the public commitment arm, but this pledge is made in private only to the NGO health worker, so that it activates the implementation intention without offering the direct coordination device for neighbors.

These interventions are implemented between November 2013 and February 2014, covering 19,271 households in 107 villages in rural Tanore sub-district (upazila), Rajshahi district (zila), Bangladesh. Note that while our interventions are not household-specific and instead focused on groups and joint liability, the popular and sensible technology for this context is a *private* household-specific latrine, not a latrine that is shared between unrelated neighbors. We measure short-term (at the time of the assessment for rewards, roughly 3 months after the interventions began) and medium-term (12–15 months after assessment) effects of the treatments on private, household-specific sanitation investments and maintenance. Earlier, between April and June 2012, we had tested a broader set of demand and supply-side interventions to also encourage investment in hygienic latrines in this same location (Guiteras et al., 2015). The group commitments and joint incentives – which are the focus of this study – were implemented around one and a half years after the interventions described in Guiteras et al. (2015) were completed. We conduct all our analysis controlling for sanitation ownership in June 2013 (which acts as the baseline for this study), which is a full year after the earlier round of interventions were completed, so household exposure to those earlier treatments should not materially affect the comparison between our new treatments reported in this paper.

Another distinguishing characteristic of this study is that while our earlier research primarily focused on the initial sanitation investment decision, we now carefully measure proper use and maintenance beyond the initial adoption. Sustaining intervention effects has been an important challenge for the sanitation sector (Coffey et al., 2014, Orgill-Meyer et al., 2019, Pakhtigian et al., 2021, Deutschmann et al., 2021). Hygienic latrines only produce health benefits if they are consistently used and are kept in good condition so that fecal pathogens are safely isolated from the environment. This requires each household to incur time and materials costs to keep the latrine clean, conduct maintenance and dispose of waste properly.

We find that group-level monetary reward has the strongest impact in the short term, inducing an 7.5 to 12.5 percentage point (pp) increase in the share of households with hygienic latrines. The public commitment treatment caused a 4.2 to 6.3 pp increase in that same period. Neither the non-monetary reward nor the private commitment treatments had statistically significant impacts. In the medium term, the effect of the monetary reward dissipates relative to the comparison group, while the effect of the public commitment treatment persists. We find that in the case of both the monetary reward and public commitment treatments, households tended to meet the short-run assessment criteria for hygienic status through small, relatively inexpensive improvements to or repairs of existing latrines, rather than making large investments in major improvements or on entirely new latrines. In the public commitment group, households tended to maintain these small improvements into the medium-term, while those in the monetary reward group tended to let these improvements depreciate.

Our research adds to a vibrant literature on barriers to sanitation adoption. Much of the earlier work explores various determinants of adoption, such as microfinance loans to overcome credit constraints (BenYishay et al., 2017, Smets et al., 2021), education and motivation to overcome information deficiencies (Pattanayak et al., 2009, Gertler et al., 2015), and targeted subsidies to increase affordability (Guiteras et al., 2015, Cameron et al., 2021). Our distinctive contribution is to design and test a new set of interventions inspired by the observation that sanitation adoption decisions are likely inter-linked across households, because they generate public health externalities and because social norms are important drivers of behavior. Under those conditions, it may be possible to induce sanitation investments and maintenance choices that improve community health using creative social and financial interventions that encourage positive interactions with neighbors.

There has been much academic and policy interest in “community led total sanitation” (CLTS) interventions (Kar and Pasteur, 2005, Pattanayak et al., 2009, Pickering et al., 2015), which aim to bring the community together to jointly discuss the public health externality problems. Our social and financial interventions are conceptually linked to CLTS, in that they are designed to make most salient the joint-commitment and public-promise aspects of CLTS. CLTS also often contains a large informational component, but that is not the focus of the randomized treatments we test.

Our experimental design is closely tied to theories of social image and reputational concerns (Benabou and Tirole, 2003, Bénabou and Tirole, 2006). If a person’s utility depends on others’ views about her, then having her make a public commitment gives her an opportunity to signal her type to others, and may also act as a disciplining device to ensure that she follows through on that commitment. Karing (2021) shows that giving parents an ability to signal their child’s vaccination status improves adherence to vaccine schedules. In our setting, public commitments may be additionally valuable because reputational concerns persist and can produce long-term behavior change in a way that a short-run monetary incentives cannot. This theory also produces a sharper empirical test, in that if social image is important, we would expect households to invest in latrine features that are more easily observable by neighbors, such as pit covers that sit above ground outside the toilet structure, as opposed to ceramic pans and water seals inside the toilets that are not as publicly visible.

Even absent any public health externality, sanitation investments are thought to be privately beneficial for dense populations like in rural South Asia, the setting of our study (Hathi et al., 2017). As such, our research is also linked to the broader literature on the surprisingly low adoption of efficacious technologies with the potential to address important development challenges, such as drinking water disinfectants (Ashraf et al., 2010), agricultural technologies (Duflo et al., 2011, BenYishay and Mobarak, 2019, Udry, 2010), nutritional supplements (Maluccio et al., 2009), rainfall insurance (Cole et al., 2014), improved cookstoves (Berkouwer and Dean, 2022, Mobarak et al., 2012), and migration (Bryan et al., 2014).

The paper proceeds as follows: Section 2 describes the study setting and the sample; Section 3 describes our interventions and experimental design; Section 4 describes our data; Section 5 presents our estimation equations and results, with reduced-form treatment effects in Section 5.1 and mechanisms in Section 5.2; Section 6 concludes.

## 2 Setting and sample

This study was conducted with 19,271 households in 107 villages in 4 unions^1^ of Tanore upazila (sub-district) of Rajshahi district, Bangladesh. Tanore is located in a poor region of the country - sub-district level poverty mapping of Bangladesh in 2016 places Tanore at a moderate level of poverty (BBS, 2020). These villages had been the site of a randomized evaluation of a set of interventions designed to study interdependencies in household investment in hygienic latrines (Guiteras et al., 2015). We refer to this first set of interventions as the “first set of interventions” or the “demand study interventions”, and the second set, the focus of this paper, as the “second set of interventions” or the “incentives for use interventions”. The study area was chosen in part because of its low level of latrine coverage: at the time of the demand study baseline, 30.8% of households reported a regular level of open defecation among adults, 50.4% reported that they had access to a hygienic latrine and 40.1% owned a hygienic latrine. This first set of interventions was conducted February 2012–August 2012, with baseline data collected December 2011–February 2012 and four rounds of followup data collected through April 2012–June 2013. Baseline data from the first set of interventions show that around 10% of the households are headed by females (Guiteras et al., 2015). Household heads, on average, have approximately 6 years of completed education. Around 70% of households in the study area work in agriculture, while around 30% of households do not own any land. Around a third of the households report to have not eaten proper meals during Monga (hungry season). A similar proportion of households does not own a cell phone or have access to electricity. 

Guiteras et al. (2015) show that subsidies increase adoption of hygienic latrines, both directly – among households winning a subsidy voucher in a public lottery – and indirectly – the share of subsidy winners was randomized at the community level, and as this “saturation” increased, investment increased among both subsidized and unsubsidized households. The current study was intended to understand how to sustain or increase these gains.

In our 107 study villages, we created 1236 groups of approximately 14–17 neighboring households, roughly 4–16 groups per village, and the incentives-for-use interventions were conducted at this group level. See SM1 of the Online Supplementary Materials for details on the group formation process. While the unit of intervention was the group, randomization was at the village level. All households in the four study unions were included in the group formation process. The intervention was carried out by 15 health motivators who were supervised by 2 field supervisors. The overall program was managed by an area coordinator.

## 3 Interventions and experimental design

**Fig. 1:**
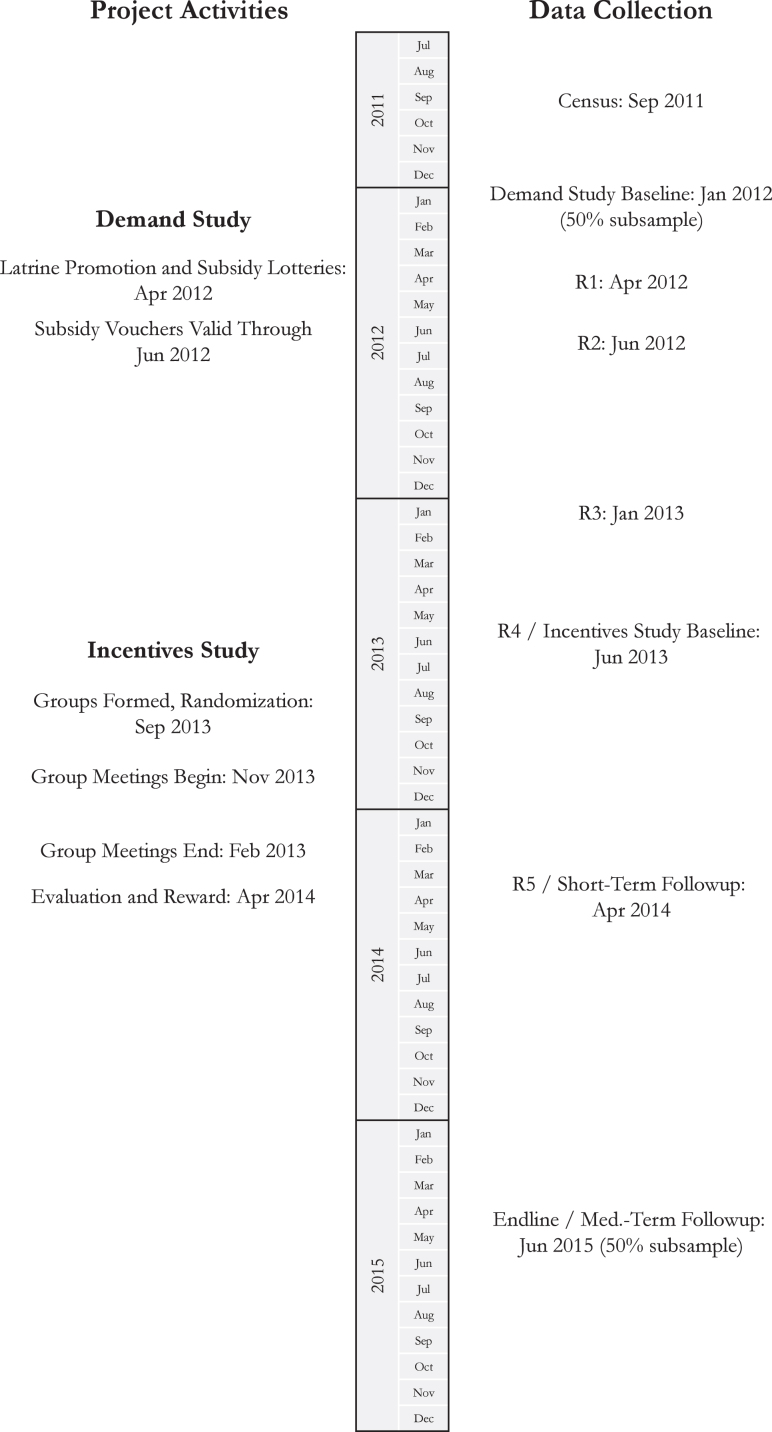
Timeline for a typical village.

In this section, we describe the treatments and the randomization. A timeline for a typical village is provided in Fig. 1.

### 3.1 Common intervention

All 980 treatment groups (in 84 treatment villages) received a basic intervention consisting of monthly meetings for three consecutive months with a Health Motivator to encourage investment in and maintenance and use of hygienic latrines.^2^ Health Motivators, trained by and contracted from our implementation partner, the Village Education Resource Center (VERC), discussed the health risks of open defecation and unhygienic sanitation practices, the collective nature of the problem (i.e., the externality in non-technical terms), the types and costs of hygienic latrines, and the current level and monthly change in the share of households with or advancing towards a hygienic latrine.^3^

In the common as well as in the cross-cutting interventions, the Health Motivator provided both a general, conceptual definition of a hygienic latrine and a specific, technical definition. The conceptual definition emphasized that a hygienic latrine was one that:

1. Limits the spread of diseases caused by feces in the water and keeps the environment pollution free;
2. Confines feces in an enclosed pit so that they cannot be seen or smelled;
3. Prevents flies or other insects from entering the pit.

The specific, technical definition listed the characteristics based on which a latrine was judged to be hygienic, in particular:

1. There must be a slab and it cannot be broken.
2. There must be a water-seal (locally known as ‘gooseneck’ or ‘siphon’) and it cannot be broken.
3. Different latrine components such as rings, delivery pipe, Y-junction (whenever applicable), pit cover (whenever applicable), etc. should be functional and without any leaks.
4. There should not be any feces in or around the latrine.
5. The latrine cannot pollute the environment. In particular, the latrine/delivery pipe can only discharge the waste into a sealed pit and not to the external environment (for example, a stream or just out in the open).

These characteristics of a hygienic latrine were relayed to participants at each of the three group meetings.^4^ Participants were made aware of the fact that for latrines to be considered hygienic all the above mentioned requirements had to be met by the specified deadline, approximately four months after the intervention began.

Health Motivators also emphasized that a latrine’s hygienic status was not just determined by the collection of parts, but depended on maintenance, repair and sanitary use. Discussions, both with the group and with individual households, emphasized small improvements or repairs that could be made to achieve hygienic status, and how to maintain hygienic status once it was achieved.

### 3.2 Reward treatments

There were two reward treatments, monetary and non-monetary, both of which were conditioned on both the household’s own status and the share of households in the group achieving hygienic latrine status. This element of “joint liability” was intended to incentivize households to motivate and assist each other. This feature is reminiscent of Grameen Bank-style “group lending” programs with joint liability, in which an applicant receives a microcredit loan only if her group members repay their loans (Ghatak and Guinnane, 1999). While the joint liability can motivate new investments in sanitation, it could also create excessive pressure. RCTs in the microfinance context have found that joint liability outperforms individual liability in Mongolia (Attanasio et al., 2015), but not in Philippines (Giné and Karlan, 2014).

The monetary reward consisted of a cash payment to the household if, at the end of the intervention period, (a) the household owned a hygienic latrine and (b) the share of households in that group with a hygienic latrine was above a designated threshold. Hygienic was defined as described in Section 3.1 above. The reward was BDT 250 (USD 3.33) in groups that surpassed the lower of the two thresholds and BDT 500 (USD 6.67) in groups that surpassed the higher of the two thresholds.^5^ For comparison, the cost of building a single-pit hygienic latrine was approximately BDT 2350 (USD 31.33), while common improvements to existing latrines that would be necessary to reach hygienic status cost substantially less, e.g., a new water seal BDT 65 (USD 0.87), delivery pipe BDT 360 (USD 4.8).

The non-monetary reward consisted of a certificate of hygiene attainment from the local government, presented to qualifying households in a public ceremony.^6^ The non-monetary reward used the same standard for “hygienic” as the monetary reward.

Thresholds were determined based on baseline hygienic latrine ownership by union. In three of the four unions, the lower threshold was set at one-third of households and the upper threshold at two-thirds. In one union with significantly lower hygienic latrine ownership at baseline, the lower and upper threshold were set at one-quarter and one-half, respectively. A lower threshold was set such that even low baseline-ownership groups would feel they could attain something, while high baseline-ownership groups would have something to reach for. We also chose thresholds that were simple and easy to explain at a group meeting: a phrase like “two out of every three households” is easier to understand than a phrase like “sixty-six percent of all households”.

**Table 1:** Randomization.

Reward	Commitment		
	None	Private	Public
None	A: 8 villages; 121 groups	B: 11 villages; 177 groups	C: 11 villages; 69 groups
	1898 households (9.8%)	2626 households (13.6%)	1088 households (5.6%)

Monetary	D: 10 villages; 79 groups	E: 5 villages; 58 groups	F: 9 villages; 97 groups
	1159 households (6.0%)	885 households (4.6%)	1568 households (8.1%)

Certificate	G: 12 villages; 145 groups	H: 9 villages; 110 groups	I: 9 villages; 124 groups
	2314 households (12.0%)	1694 households (8.8%)	1970 households (10.2%)

Pure control	J: 23 villages; 256 groups		
	4069 households (21.1%)		

The assessment was conducted approximately four months after the intervention began, after three group meetings with the Health Motivator. Health Motivators did not conduct assessments in villages where they had worked. See Section 4.3 for a discussion of the assessment process. The full survey instrument is provided in Section SM5 of the Supplementary Materials. Households knew the deadline for achieving hygienic status, and that the assessment would occur within one-two weeks after the deadline, but did not know the specific day of the assessment.

### 3.3 Commitment treatments

There were two commitment treatments, public and private.

In the public commitment arm, during each group meeting, members from all the households of a group were encouraged to make a public pledge that those who did not yet have hygienic latrines would meet hygienic latrine standards as set by the project. Those with hygienic latrines pledged to help others reach the goal within the time limit set by the project. The script of the pledge, in English translation, was: “I hereby promise before everyone present that I will do my best to set up hygienic latrines or improve existing ones into hygienic latrines for myself and for my neighbors by [end date].” In the public commitment arm, this pledge was repeated at the end of each monthly group meeting.

In the private commitment arm, health motivators visited each household in the group after each group meeting. The member of the household attending the meeting would be encouraged by the Health Motivator to make a commitment before the health motivator that he/she would transform their unhygienic latrines to hygienic ones within the time limit set by the project. The script of the pledge was identical to that in the public commitment arm.

### 3.4 Experimental design

The reward and commitment treatments lead to a 3 × 3 design, plus a pure control group. The design is summarized in Table 1. Although the treatments were implemented at the group level, randomization was conducted at the village level because of the potential for spillovers within village. We allocated approximately 25% of villages to pure control, and then the remaining villages were intended to be allocated equally across the commitment and reward treatments. With 107 villages (84 treatment villages), we did not expect to have adequate power to detect interaction effects. The randomization was stratified by union. Because of a coding error, there is some imbalance in the number of villages per cell. Most significantly, the basic treatment only cell was under-populated (8 villages), so we use Wild bootstrap standard errors for inference in our group-level analyses (MacKinnon and Webb, 2017, Roodman et al., 2019). Descriptive statistics and balancing tests for key baseline observables are provided in Table 2.

**Table 2:** Descriptive statistics and balance tests.

Treatment:	All	Pure	Basic	Reward		Commitment		Joint
		Control	Only	Monetary	Certificate	Private	Public	p-val.
	Mean	Mean	Mean	Diff	Diff	Diff	Diff	
	(S.D.)	(S.D.)	(S.D.)	[S.E.]	[S.E.]	[S.E.]	[S.E.]	
	(1)	(2)	(3)	(4)	(5)	(6)	(7)	(8)
*Group characteristics:*								
Group size (num. HH)	15.59	15.89	15.69	−0.25	0.09	−0.60	0.27	0.286
	(2.74)	(2.78)	(2.48)	[0.51]	[0.46]	[0.47]	[0.48]	
Share landless	0.350	0.363	0.312	0.054	0.025	0.031	0.027	0.852
	(0.243)	(0.251)	(0.208)	[0.042]	[0.033]	[0.034]	[0.036]	
Regular open defecation by adults	0.263	0.270	0.199	0.082${}^{*}$	0.035	0.073	0.049	0.675
(HH self-report)	(0.250)	(0.251)	(0.224)	[0.045]	[0.045]	[0.049]	[0.044]	
Density (mean num. HH within 50 m)	12.33	11.69	13.67	−1.81	−1.03	−0.87	−1.18	0.884
	(6.07)	(5.74)	(6.58)	[1.39]	[1.43]	[1.43]	[1.55]	
Village leader in group	0.153	0.156	0.116	0.072${}^{**}$	0.032	0.009	0.060${}^{*}$	0.376
	(0.360)	(0.364)	(0.321)	[0.035]	[0.032]	[0.032]	[0.032]	
*Baseline latrine ownership:*								
Owns no latrine	0.403	0.394	0.377	0.014	0.012	0.040	0.008	0.187
	(0.202)	(0.195)	(0.188)	[0.026]	[0.029]	[0.033]	[0.029]	
Owns any latrine	0.597	0.606	0.623	−0.014	−0.012	−0.040	−0.008	0.187
	(0.202)	(0.195)	(0.188)	[0.026]	[0.029]	[0.033]	[0.029]	
Owns non-hygienic latrine	0.214	0.244	0.212	0.000	−0.015	−0.007	0.001	0.903
	(0.154)	(0.155)	(0.151)	[0.024]	[0.025]	[0.025]	[0.026]	
Owns hygienic latrine	0.397	0.374	0.435	−0.023	−0.006	−0.043	−0.016	0.625
	(0.218)	(0.201)	(0.203)	[0.040]	[0.044]	[0.050]	[0.041]	
*Baseline latrine access:*								
No latrine access	0.211	0.196	0.170	0.042	0.024	0.061	0.028	0.525
	(0.223)	(0.205)	(0.209)	[0.039]	[0.039]	[0.043]	[0.040]	
Access to any latrine	0.789	0.804	0.830	−0.042	−0.024	−0.061	−0.028	0.525
	(0.223)	(0.205)	(0.209)	[0.039]	[0.039]	[0.043]	[0.040]	
Access to hygienic latrine	0.491	0.466	0.533	−0.031	0.001	−0.053	−0.015	0.496
	(0.257)	(0.242)	(0.232)	[0.046]	[0.052]	[0.056]	[0.048]	
*Sample sizes:*								
Villages	107	23	8	24	30	25	29	
Groups	1236	256	121	234	379	345	290	
Households	19,271	4069	1898	3612	5978	5205	4626	

*Notes:* this table presents summary statistics (means and standard deviations) of key baseline variables for all villages (Column 1), pure control villages (Column 2) and villages where groups received only the basic health messaging treatment (Column 3). Standard deviations are in parentheses. Columns 4–7 show estimated coefficients for indicators for the village-level treatments (monetary reward, reward certificate, private commitment, public commitment) in regressions where the baseline variable is the dependent variable, and the basic health messaging treatment is the omitted category. Estimated standard errors robust to clustering at the village level are in brackets. Column 8 shows the p-value on a joint F-test of significance of the treatment indicators. Sample sizes do not sum because villages may be assigned to one reward treatment, one commitment treatment, one from each category, or neither. (See discussion of experimental design in the text.) ${}^{*}$ p<0.10, ${}^{**}$ p<0.05, ${}^{***}$ p<0.01.

## 4 Data

The full timeline of all data-collection activities for a typical village is presented in Fig. 1.

### 4.1 Previous surveys

As noted above, several rounds of surveys had been completed for the previous demand study. Specifically, these were: a census, a baseline (conducted on a 50% subsample of households in each village) and three monitoring rounds focused on latrine improvements and condition. In this study, we primarily use: (1) the census data on landless status, social networks, in particular who households identify as local leaders; and (2) the third followup monitoring round, in which we collected location data to assist in creating groups and to construct density measures.

### 4.2 Baseline latrine coverage

A few months before beginning the interventions in this study, we conducted what we will refer to as the “baseline” survey for this study.^7^ We collected data from all households on latrine ownership, including detailed information on the condition of each household’s latrine. This allowed us to classify each household’s latrine as “none”, “non-hygienic”, or “hygienic”. We include hanging latrines (an exposed platform over a marsh or stream) and uncovered pits in the “none” category, since these are effectively the same as open defecation in terms of disease, and cannot possibly be transformed into a hygienic latrine through simple improvements. This provided our baseline measures of our outcome variables. We used these data to determine union-specific thresholds for the reward treatments when designing the interventions.

### 4.3 Short-term outcomes

At the end of the intervention, we collected data from all households on latrine investment, use and maintenance. In reward and recognition groups, these data were collected as part of the reward determination process. These assessment data were collected 1–2 weeks after the end of program activities, or roughly 3 months after program activities began; households knew the general time frame but not the specific date. For budgetary reasons and because Health Motivators already had the training to assess latrine conditions, we used Health Motivators to collect these data, but no Health Motivator collected data in a village where he or she had led an intervention. The Health Motivators that collected data were not informed of the village’s treatment status, nor which Health Motivators had led the intervention in that village. Similarly, Health Motivators were not told which of their peers had collected the evaluation data in villages where they had led the intervention. In addition, to understand the mechanisms for the success or failure of the intervention, households were asked whether they received any assistance (financial, labor, advice) from community members, and whether they were pressured or encouraged by others in their group.

The criteria by which a household’s latrine was judged “hygienic” for the purpose of the reward are given in Section 3.2. See the Supplementary Materials for precise definitions for coding the outcome variables of interest (Section SM3) and the survey instrument (Section SM5). Data were collected following the same protocol in all villages, regardless of treatment status.

**Table 3:** Program effects: Hygienic latrine ownership.

	Short term		Medium term	
	(1)	(2)	(3)	(4)
Monetary reward	0.125${}^{***}$	0.078${}^{***}$	0.047	0.012
	(0.034)	(0.015)	(0.030)	(0.020)
	[0.052, 0.200]	[0.045, 0.110]	[−0.018, 0.111]	[−0.031, 0.054]
Reward certificate	0.044	0.011	0.043	0.019
	(0.037)	(0.012)	(0.035)	(0.022)
	[−0.047, 0.130]	[−0.016, 0.037]	[−0.041, 0.124]	[−0.032, 0.067]
Private commitment	0.008	0.009	0.013	0.013
	(0.038)	(0.012)	(0.039)	(0.025)
	[−0.076, 0.098]	[−0.019, 0.036]	[−0.079, 0.102]	[−0.047, 0.068]
Public commitment	0.063${}^{*}$	0.045${}^{***}$	0.072${}^{**}$	0.057${}^{***}$
	(0.036)	(0.015)	(0.028)	(0.017)
	[−0.018, 0.144]	[0.012, 0.078]	[0.012, 0.132]	[0.023, 0.091]
Baseline share owning hyg. lat.		0.709${}^{***}$		0.513${}^{***}$
		(0.022)		(0.036)
Share of households landless		−0.083${}^{***}$		−0.089${}^{***}$
		(0.017)		(0.032)
Union FEs	Yes	Yes	Yes	Yes

Diff.: Monetary – Public	0.062	0.033	−0.025	−0.045
	(0.046)	(0.020)	(0.042)	(0.030)
p-value	0.182	0.089	0.544	0.140
Diff.: Monetary – Certificate	0.081	0.067	0.004	−0.007
	(0.039)	(0.015)	(0.032)	(0.019)
p-value	0.038	0.000	0.899	0.732
Diff.: Public – Private	0.055	0.036	0.059	0.045
	(0.038)	(0.014)	(0.036)	(0.022)
p-value	0.156	0.014	0.098	0.047

Number of groups	1236	1235	1235	1234
Number of villages	107	107	107	107
Omitted category mean	0.451	0.451	0.544	0.544
Omitted category S.D.	(0.189)	(0.189)	(0.255)	(0.255)

*Notes:* the dependent variable is the share of households in the group owning a hygienic latrine. Columns (1) and (2) report short-term effects (at the time of assessment); columns (3) and (4) report medium-term effects (12–15 months after assessment). Observations (groups) are weighted by the number of households. The comparison group consists of groups that received the meetings only treatment. Pure control villages are included as a separate category to enhance precision. Standard errors clustered at the village level. Standard errors clustered at the village level in parentheses. Wild cluster bootstrap (9999 repetitions, Webb weights) 95% confidence intervals, resampling at the village level, in brackets for the coefficients of interest. ${}^{*}$ p<0.10, ${}^{**}$ p<0.05, ${}^{***}$ p<0.01.

### 4.4 Medium-term outcomes

Medium-term outcome data were collected 12–15 months after the assessment (June 2015–August 2015). This round served as an endline survey for the project as a whole, and so included several lengthy socio-economic and demographic modules. Because of budget constraints, we conducted this survey with the 50% subsample surveyed at baseline in the demand study (see Section 4.1 above). This led to some imbalance in the endline subsample across groups. First, the baseline subsampling was stratified by village, and since the sub-village groups for this study had not been created yet, randomness led to some imbalance. Second, some new households had been formed since the demand study census. To avoid under-sampling groups, in groups with fewer than six households included in the endline sample, we randomly selected additional households from the group for a brief “top-up” survey on latrine status. We used the same modules on latrine status, use and maintenance as with those households receiving the full endline survey.

## 5 Estimation and results

### 5.1 Program effects

The primary outcome of interest is the group-level share of households owning and maintaining an hygienic latrine, as defined in Section 4 above. Secondary outcomes of interest include the share of households with access to a hygienic latrine, owning any latrine, with access to any latrine, and engaging in open defecation. We provide detailed definitions for all of these outcomes in the Supplementary Materials (Section SM3).

To measure reduced-form effects of our treatments, we estimate

(1) $$y_{gv}=\beta_{1}\text{Incent}{}_{v}+\beta_{2}\text{Cert}{}_{v}+\beta_{3}\text{Priv}{}_{v}+\beta_{4}\text{Publ}{}_{v}$$

$$+\delta y_{0gv}+\gamma \text{ShareLandless}{}_{gv}+\beta_{0}\text{PureControl}{}_{v}+\varphi_{u}+{ɛ}_{gv}$$

where $y_{gv}$ is the outcome variable of interest (e.g., share of households owning a hygienic latrine) for group g in village v, $\text{Incent}{}_{v}$ and $\text{Cert}{}_{v}$ are indicators for village v’s reward treatment assignment (financial incentive and social incentive, respectively), $\text{Priv}{}_{v}$ and $\text{Publ}{}_{v}$ are indicators for village v’s commitment treatment assignment (private commitment and public commitment, respectively), $y_{0gv}$ is the pre-intervention level of the outcome variable, so estimates with this control are an ANCOVA specification (McKenzie, 2012), $\text{ShareLandless}{}_{gv}$ is the share of landless households in the group, which proxies for the financial resources available to the group as a whole, $\varphi_{u}$ is a set of union fixed effects, and ${ɛ}_{gv}$ is an error term which may be correlated at the village level (the level of randomization).^8^

The coefficients $\beta_{1}$ and $\beta_{2}$ represent the effects of the reward treatments, controlling for potential imbalances in the commitment treatment, while coefficients $\beta_{3}$ and $\beta_{4}$ represent the effects of the commitment treatment, controlling for potential imbalances in the reward treatment.^9^ The omitted category in our main specifications consists of villages receiving the common, “meetings-only” treatment, but no other treatment (cell A in Table 1), and our estimates should be interpreted as effects relative to this basic, common treatment. We include the pure control villages in the regressions to enhance precision, and the “effect” of being in the pure control group relative to the meetings-only treatment is captured by $\beta_{0}$. In other words, the effect of the meetings-only treatment relative to the pure control group is $-\beta_{0}$. In the main text, we focus on the effects of the incentive and commitment treatments relative to the common treatment. We present and discuss the largely null effects of the common treatment compared to pure control in Appendix A.

Our main outcome of interest is the share of households in the group owning a hygienic latrine. As discussed in Sections 3.1, 4, “hygienic” refers not just to the physical components (especially, water seal and sealed pit), but also the condition of these components (e.g., no leaks). Ideally, we would like to estimate effects on actual use and open defecation but these are difficult to measure objectively. Households may overstate the condition of their latrine and understate their rate of open defecation because of social desirability bias, and this is especially likely when a reward or their reputation may be at stake. In contrast, whether a household owns a hygienic latrine and whether that latrine is being kept clean can be assessed in a fairly objective manner. Our evaluation visits were unannounced so households could not meet our criteria by rushing to complete a repair or a major cleaning, although we cannot rule out that news of the assessment team’s arrival in the village would spread in time to allow a household to conduct some minor cleaning.

#### Short-term results

**Fig. 2:**
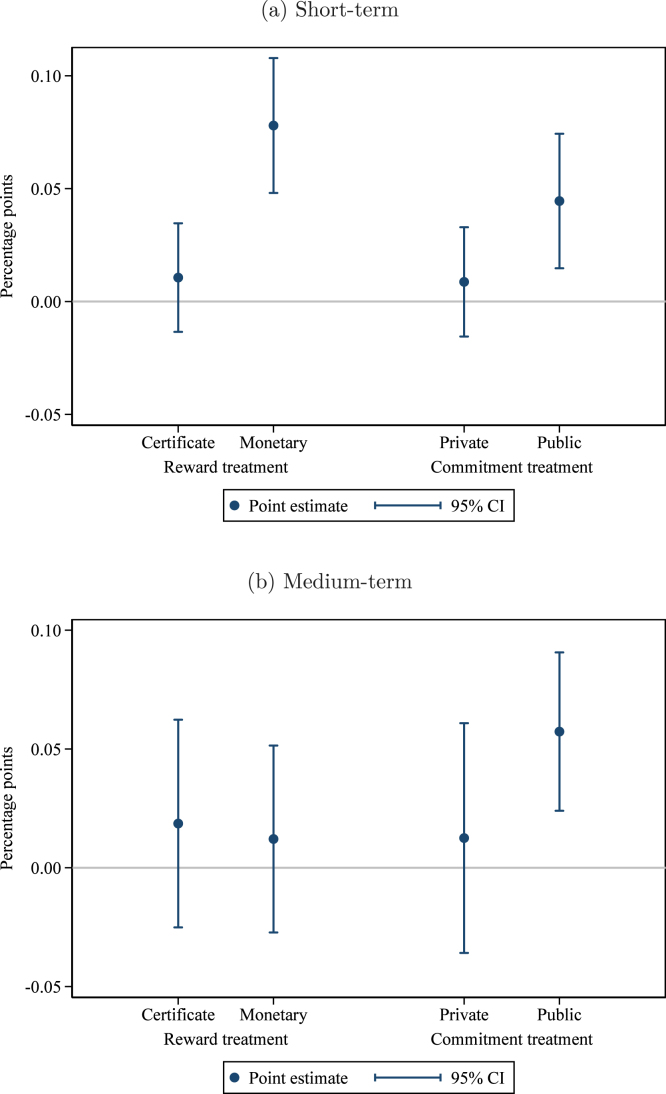
Program effects: Hygienic latrine ownership. *Notes:* this graph presents estimated treatment effects of the interventions on the share of households in the group owning a hygienic latrine. Panel (a) presents effects in the short term (at the time of assessment); panel (b) in the medium term (12–15 months after assessment). The regression controls for the baseline level of the outcome variable, the share of households in the group that are landless, and union fixed effects. Observations (groups) are weighted by the number of households. The comparison group consists of groups that received the meetings only treatment. Pure control villages are included as a separate category to enhance precision. 95% confidence intervals use standard errors clustered at the village level (the level of randomization).

Columns (1) and (2) of Table 3 report the short-term effects of the different treatment arms. Column (1) estimates Eq. (1) with union fixed effects but no other controls, while in column (2) we add controls for the baseline value of the outcome variable and the share of households in the group that are landless. Column (2) represents our pre-specified preferred model. The unit of observation is the group, and groups are weighted by the number of households in the group, although results are not sensitive to weighting (see Appendix Table B1). In parentheses, we report standard errors robust to clustering at the village level (the level of randomization). In brackets, we report 95% confidence intervals from wild cluster bootstrapping for our coefficients of interest (MacKinnon and Webb, 2017, Roodman et al., 2019). Estimated coefficients from column (2), with 95% confidence intervals, are plotted in Fig. 2(a). Estimated differences between key pairs of treatments are presented, with p-values, at the bottom of the table.

As shown in Table 3, the monetary reward treatment is most effective at increasing hygienic latrine ownership in the short term. The point estimate ranges from +7.8 to +12.5 percentage points (pp) depending on the specification, relative to an omitted category mean of 45.1%. The public commitment treatment increases ownership by 4.5 to 6.3 pp. The difference between the monetary reward treatment and the public commitment treatment is 3.3 percentage points in the pre-specified model, significant at the 10% level. The effects of the reward certificate and the private commitment are both economically small and statistically insignificant. Including interactions with the (de-meaned) control variables leaves the results virtually unchanged (Appendix Table B2).

**Fig. 3:**
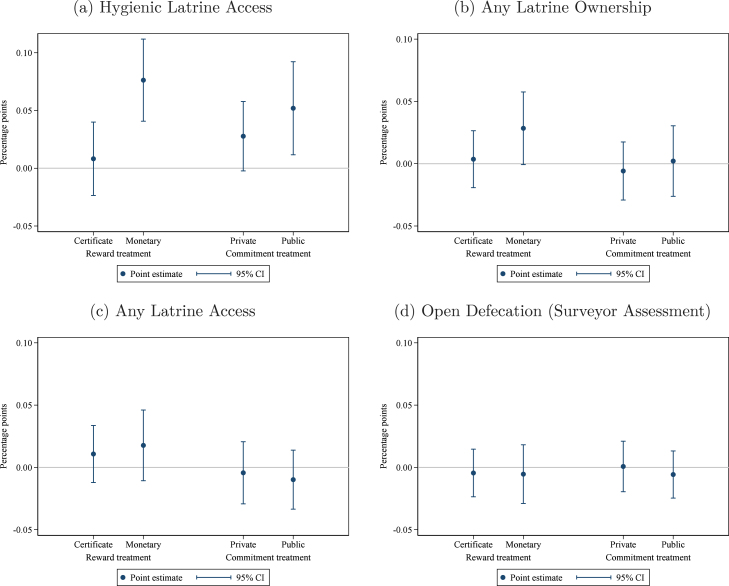
Treatment effects on secondary outcomes — Short term. *Notes:* these graphs present estimated short-term effects of the interventions on the outcome variable indicated in the figure caption. The regression controls for the baseline level of the outcome variable, the share of households in the group that are landless, and union fixed effects. Observations (groups) are weighted by the number of households. The comparison group consists of groups that received the meetings only treatment. Pure control villages are included as a separate category to enhance precision. 95% confidence intervals use standard errors clustered at the village level (the level of randomization).

To assess whether these interventions affected the sanitation environment in these communities beyond hygienic latrine ownership, we present short-term effects on secondary outcomes in Fig. 3.^10^ It is possible that the impact of these interventions on the overall health environment could be greater than just the effect on ownership if households allow others to use their hygienic latrine. However, when we use *access* to a hygienic latrine as the outcome variable rather than *ownership*, as in Fig. 3(a), we see little evidence of this. Similarly, there does not seem to be an effect on overall latrine ownership and access outside the hygienic category: in Fig. 3(b), we see that ‘*any* latrine ownership’ (including non-hygienic) is not affected except in the monetary reward treatment, and the effect there is small (+2.8 pp) and only marginally statistically significant (p<0.10). We see little impact on open defecation, as shown in Fig. 3(d).^11^ Together, these results suggest that the successful interventions appear to be mostly working by inducing households to upgrade or better maintain existing latrines, rather causing new latrines to be built. We will return to this hypothesis when we examine household behavior and investment in Section 5.2.1 below.

#### Medium-term results

To measure effects in the medium-term, we again estimate Eq. (1) using endline ownership (12–15 months after the intervention) as the outcome variable. The results are reported in columns (3) and (4) of Table 3, with estimated coefficients and 95% confidence intervals from the pre-specified preferred specification (column (4) in the table) plotted in Fig. 2(b). The effect of the monetary reward has faded (+1.2 to +4.7 pp, insignificant at conventional levels) relative to the comparison group,^12^ while the effect of the public commitment treatment persists (+5.7 to +7.2 pp, p<0.01). The difference between the monetary reward treatment and the public commitment treatment is just short of statistical significance in our preferred specification (point estimate −4.5 pp, p=0.14). As in the short term, neither the reward certificate nor the private commitment have statistically significant effects. Again, these results are not sensitive to weighting (Appendix Table B1) nor to including interactions with the (de-meaned) control variables (Appendix Table B2).

**Fig. 4:**
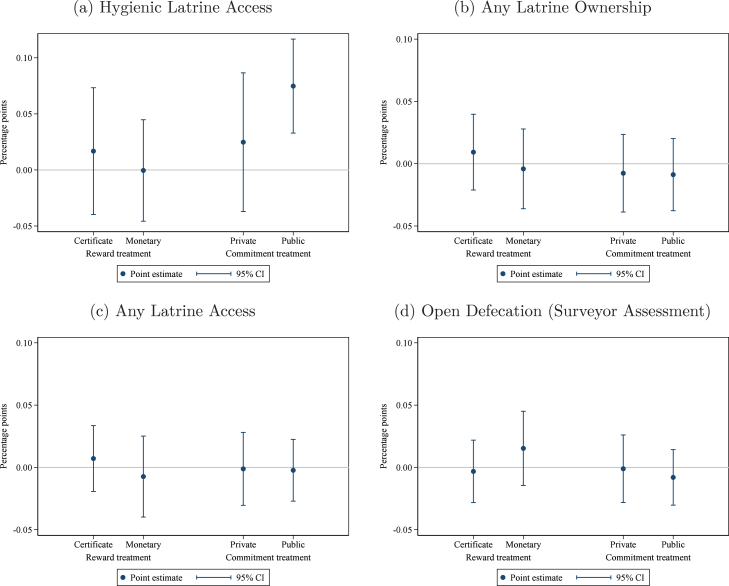
Treatment effects on secondary outcomes — Medium term. *Notes:* these graphs present estimated medium-term effects of the interventions on the outcome variable indicated in the figure caption. The regression controls for the baseline level of the outcome variable, the share of households in the group that are landless, and union fixed effects. Observations (groups) are weighted by the number of households. The comparison group consists of groups that received the meetings only treatment. Pure control villages are included as a separate category to enhance precision. 95% confidence intervals use standard errors clustered at the village level (the level of randomization).

When we examine medium-term effects on our secondary outcomes of interest,^13^ we find that there may be some enhancement of the effect of the public commitment treatment on the community environment beyond ownership, as its effect on access (+7.5 pp, Fig. 4(a)) is slightly greater than the effect on hygienic latrine ownership (+5.7 pp). While this is plausible given that the public commitment treatment placed greater emphasis on collective responsibility than the other treatments, we consider this only suggestive, since the marginal gain in ‘access’ over ‘ownership’ is only an extra 2 pp and this difference is not statistically significant. As with the short-term results, the ownership effects are concentrated on ‘hygienic latrines’ (the target of our intervention design), not ‘any latrine’ (Fig. 4(b)). Similarly, the effects of the interventions on open defecation remain null (Fig. 4(d)).

### 5.2 Mechanisms

#### 5.2.1 Household investments and behavior

The clear pattern that emerges is that monetary rewards produce the largest short-term gains in hygienic latrine ownership, but this effect dissipates over the following 12–15 months; in contrast, the public commitment treatment produces a steady increase which persists for at least a year or more. In this section, we delve into our detailed data on latrine components to understand the specific investment decisions households made under different treatments that could produce these patterns.

First, the basic program effects we show in Table 3 could have been produced by either households investing in entirely new hygienic latrines, or making smaller investments to maintain or improve their existing latrines. In Table 4, we show that the latter mechanism was at work — estimated effects on new latrine construction are small in magnitude and not statistically significant (columns (1) and (2)), while we do see statistically significant effects on installation of new latrine components (columns (3) and (4)).^14^

**Fig. 5:**
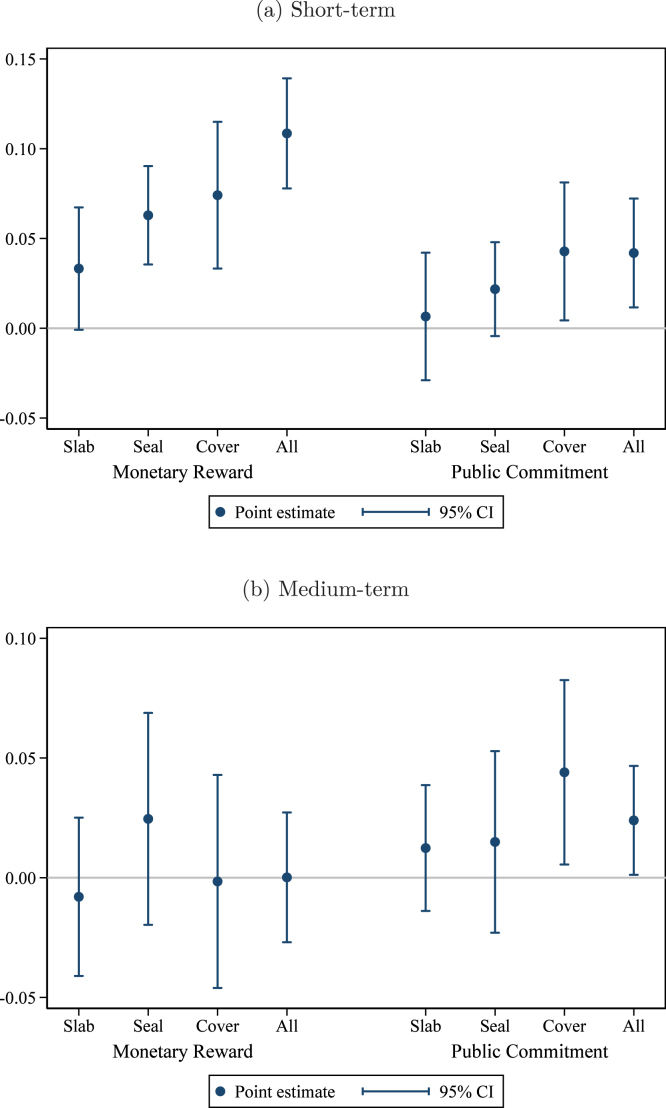
Latrine components functional and unbroken. *Notes:* these graphs present estimated treatment effects of the Monetary Reward and Public Commitment treatments on indicators for whether the household owns a latrine with the component indicated functional and unbroken. “Cover” refers to the pit cover and rings. “All” indicates that all of the slab, seal, and pit cover and rings are functional and unbroken. The top panel shows short-term effects and the bottom panel shows medium-term effects. The comparison group consists of households in villages receiving only the basic health intervention. Households in pure control villages are included to increase precision. The regression controls for group-level baseline hygienic latrine ownership, group share of landless households, and union fixed effects. 95% confidence intervals use standard errors clustered at the village level (the level of randomization).

Next, we investigate the specific latrine components the households prioritized for investment. We show effects on the three most important components that – properly installed, functional and unbroken – are necessary for a latrine to be classified as hygienic. These components are a concrete slab (on which the ceramic pan is placed, where the user squats), a water seal (to prevent bad smells and flies from moving in and out of the pit where the waste is stored), and the cover for the latrine pit and rings that safely confine the accumulated waste and prevent any leakages. In Fig. 5(b), consistent with the results on our main outcome (ownership of a hygienic latrine), we see the largest short-term effect from the monetary reward treatment, with statistically significant gains in each of the three components individually, as well as an indicator for all three. However, these gains dissipate in the medium term, as shown in Fig. 5(b).^15^

**Table 4:** Household investments.

	New latrine		Components	
	(1)	(2)	(3)	(4)
Monetary reward	0.004	0.009	0.030${}^{**}$	0.032${}^{**}$
	(0.007)	(0.008)	(0.013)	(0.012)
Reward certificate	0.002	0.005	−0.004	−0.003
	(0.006)	(0.007)	(0.010)	(0.010)
Private commitment	0.003	0.003	0.022${}^{**}$	0.022${}^{**}$
	(0.006)	(0.007)	(0.010)	(0.010)
Public commitment	0.008	0.009	0.036${}^{***}$	0.036${}^{***}$
	(0.007)	(0.007)	(0.011)	(0.011)
Baseline share owning hyg. lat.		−0.065${}^{***}$		−0.024
		(0.012)		(0.021)
Share of households landless		−0.012		−0.036${}^{***}$
		(0.010)		(0.012)
Union FEs	Yes	Yes	Yes	Yes

Number of households	15,984	15,980	15,984	15,980
Number of groups	1236	1235	1236	1235
Number of villages	107	107	107	107
Omitted category mean	0.056	0.056	0.046	0.046

*Notes:* the dependent variable in columns (1) and (2) is an indicator for whether the household constructed a new latrine in the period since the beginning of the intervention. The dependent variable in columns (3) and (4) is an indicator for whether the household installed new latrine components in the same period. In both cases, data are collected in the short term (at the time of assessment). The comparison group consists of groups that received the meetings only treatment. Pure control villages are included as a separate category to enhance precision. Standard errors clustered at the village level. ${}^{*}$ p<0.10, ${}^{**}$ p<0.05, ${}^{***}$ p<0.01.

In contrast, the public commitment treatment has a more modest effect in the short term (statistically different from zero only for pit cover and rings, as well as all three), but this effect persists into the medium term, where we observe a statistically significant +2.4 pp increase in the probability that a household owns a latrine with all three key components functional and intact. It is of interest that this medium-term effect is concentrated in the functional, intact pit cover and rings (+4.4 pp, p<0.05). One characteristic that distinguishes the pit cover from the other components is that it sits outside the toilet and the toilet’s superstructure (since the pits have to be emptied periodically, and are designed to be ‘offset’ from the toilet and not directly underneath), and therefore more easily visible to neighbors. Under the ‘public commitment’ treatment, we therefore detect investments in the component that neighbors can more easily monitor. This is consistent with the formulation of theories of social image and reputational concerns (Benabou and Tirole, 2003). Under this formulation, the fact that the public commitment treatment produces lasting effects may indicate that concerns about reputation outlast the monetary incentives provided at the outset. People become uninterested when the incentives disappear, but they continue to care about saving face in front of neighbors.

Next, we examine outcomes related to latrine maintenance. We orient all variables so that one corresponds to better condition and zero to worse. We assign one to households that own a latrine with the specified desirable characteristic, and zero to households that either own a latrine without the desired characteristic or do not own a latrine. The proxies analyzed are no bad smell noticed, no leaks observed, and whether water and soap for hand-washing are present at or near the latrine.

**Fig. 6:**
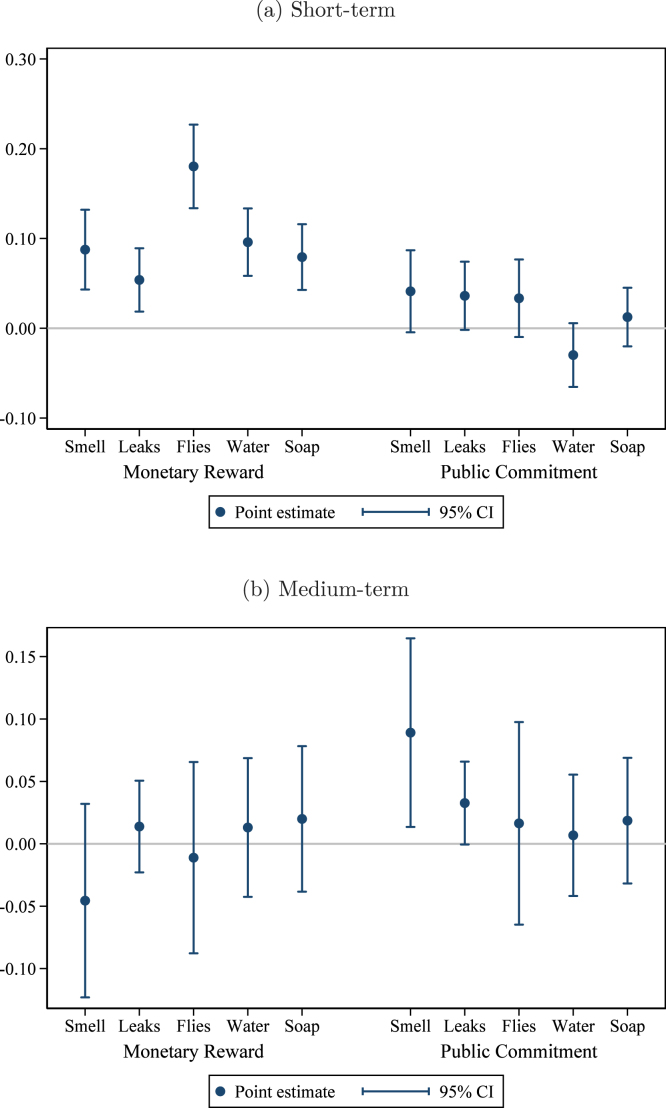
Latrine condition and maintenance. *Notes:* these graphs present estimated treatment effects of the Monetary Reward and Public Commitment treatments on indicators for whether the household owns a latrine in the condition noted. In all cases, the dependent variable is oriented so that one corresponds to better condition and zero to worse. “Smell” indicates no bad smell noticed; “Leaks” indicates no leaks observed; “Flies” indicates no flies observed; “Water” and “Water” indicate whether water and soap are available for handwashing. The top panel shows short-term effects and the bottom panel shows medium-term effects. The comparison group consists of households in villages receiving only the basic health intervention. Households in pure control villages are included to increase precision. The regression controls for group-level baseline hygienic latrine ownership, group share of landless households, and union fixed effects. 95% confidence intervals use standard errors clustered at the village level (the level of randomization).

The results, presented in Fig. 6, exhibit similar patterns to those observed for latrine components.^16^ Again, there are improvements in all dimensions in the short-run under the monetary reward treatment, which dissipate after a year. In contrast, under the public commitment treatment, there are statistically significant effects on avoiding bad smells and leaks in both the short and the medium term.

Again, smells and pit leaks are the most visible components of maintenance, as opposed to water, soap and flies inside the toilet, which are aspects that neighbors cannot easily monitor. Avoiding leaks and smells requires the household to invest in fixing broken pit covers and rings, which are precisely the components for which we observe statistically significant improvements in Fig. 5(b). In summary, the data suggest that households who were asked to make a public commitment to maintain hygienic latrines, choose to make the (relatively inexpensive) investments in latrine components and make maintenance choices that avoid the most obvious, visible failures that can create slippage into a ‘non-hygienic’ sanitation territory.

**Fig. 7:**
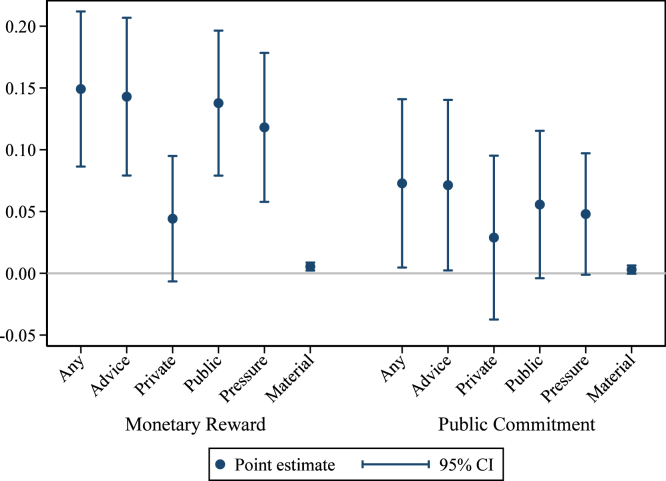
Short-term effects: Assistance from others in group. *Notes:* these graphs present estimated treatment effects of the Monetary Reward and Public Commitment treatments on indicators for different types of assistance the household reports receiving from others in the group. “Any” indicates any assistance; “Advice” indicates advice or information; “Private” indicates that the advice or information was provided in private; “Public” indicates that the advice or information was provided in public; “Pressure” indicates that the household felt pressured by others in the group to make its latrine hygienic; “Material” indicates material support, including materials, money, or labor. The comparison group consists of households in villages receiving only the basic health intervention. Households in pure control villages are included to increase precision. The regression controls for group-level baseline hygienic latrine ownership, group share of landless households, and union fixed effects. 95% confidence intervals use standard errors clustered at the village level (the level of randomization).

Finally, we study the nature of interactions between households within the same treatment group, to investigate whether the interventions generated any conversations, cooperation, advice, or reciprocity that ultimately produced the changes in investment behavior. We show effects of the treatments on indicators for whether the household reports receiving different types of assistance or information from their neighbors, or pressure from others in the group. These were only collected in our short-term survey. Fig. 7 shows that, generally speaking, all treatments led to greater assistance, advice and information sharing, so our interventions were successful in achieving the immediate, proximate goal.^17^ Households in the monetary reward treatment felt the most pressure from others in their group. However, we do not observe any clear pattern that helps explain why those conversations and assistance converted into persistent hygienic latrine maintenance effects in the public commitment treatment.

One criticism of interventions that leverage social pressure is that they can lead to conflict between households. This does not appear to have been an issue here. In the endline survey, we asked households if they had experienced conflicts with neighbors over latrines. We examine the effects of our treatments on this outcome in Table D6 in Appendix D. None of the treatments were associated with an increase in conflict, and the public commitment treatment was associated with a reduction of 0.6 pp reduction in conflicts with neighbors over sanitation issues (p<0.05), relative to a control group mean of 1.2%. These results indicate that sanitation improvements do not come at a cost of increased community tension, although conflict over sanitation appears to be rare in any case in these communities.

#### 5.2.2 Household characteristics

To examine the extent to which program effects vary with respect to household characteristics, we modify Eq. (1) in two ways: by using household-level data and by interacting household characteristics with treatments. Specifically, we estimate

(2) $$y_{hgv}=\alpha_{0}+\alpha_{1}D_{hgv}$$

$$+\overset{4}{\underset{p=0}{\sum }}\beta_{p}\cdot 1\left\{\text{Treat}{}_{v}=p\right\}+\overset{4}{\underset{p=0}{\sum }}\theta_{p}\cdot 1\left\{\text{Treat}{}_{v}=p\right\}\times D_{hgv}+\;\delta y_{0gv}+\gamma \text{ShareLandless}{}_{gv}+\varphi_{u}+{ɛ}_{hgv}$$

where $y_{hgv}$ is the outcome variable of interest for household h in group g in village v, $D_{hgv}$ is a characteristic of household h, $1\left\{\text{Treat}{}_{v}=p\right\}$ is an indicator for the treatment status of village v, i.e., p=0,1,2,3,4 refer to pure control, financial incentive, social incentive, private commitment and public commitment, respectively, and all other variables are as defined in Eq. (1). The coefficient $\alpha_{1}$ represents the level effect of characteristic D, i.e., the association of D with the outcome variable $y_{gv}$ in the comparison group, the coefficient $\beta_{p}$ represents the level effect of treatment p, i.e., the effect of treatment p on households with D=0, and the coefficient $\theta_{p}$ is the interaction between treatment p and characteristic D.^18^ We focus on the primary outcome of hygienic latrine ownership unless otherwise noted, and present results for the monetary reward and public commitment treatments, with full regression results for all treatments in Appendix E (Table E1-E3).

**Fig. 8:**
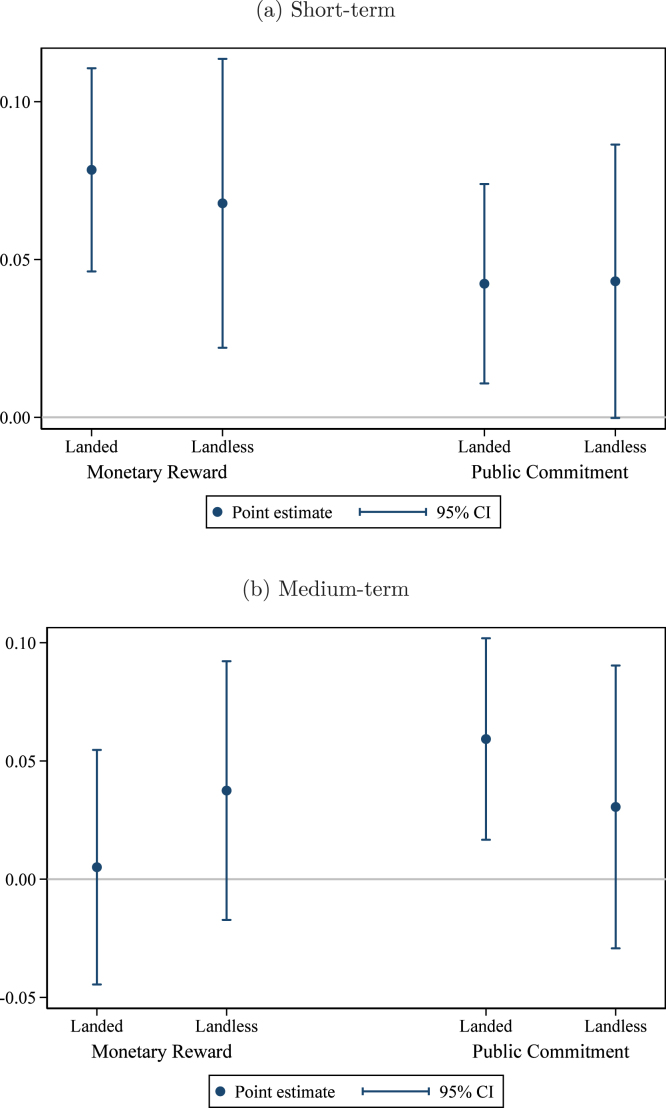
Effect on hygienic latrine ownership. *Notes:* these graphs present estimated treatment effects of the Monetary Reward and Public Commitment treatments on ownership of a hygienic latrine by household land ownership status. The top panel shows short-term effects and the bottom panel shows medium-term effects. The comparison group consists of households in villages receiving only the basic health intervention. Households in pure control villages are included to increase precision. The regression controls for group-level baseline hygienic latrine ownership, group share of landless households, and union fixed effects. 95% confidence intervals use standard errors clustered at the village level (the level of randomization). By Household’s Landless Status.

We first examine whether households’ responsiveness differ by poverty, which we proxy by landlessness. We hypothesized that landless households would be less able to respond to the non-monetary arms but might benefit from cross-subsidization in the monetary arms. In fact, in the short term, landless households responded nearly identically, as shown in Fig. 8(a). (Regression results reported in Appendix Table E2.) In the medium term (Fig. 8(b)), point estimates suggest some heterogeneity in response: both the fading of the effect of the monetary treatment and the sustained effect of the public commitment treatment is among *landed* households, although in neither case do the estimated interaction terms reach statistical significance.

We also investigate heterogeneity by the household’s baseline ownership status. Households are classified as owning none (the base category), owning a non-hygienic latrine, or owning a hygienic latrine. We hypothesized that households owning a non-hygienic or hygienic latrine at baseline would be relatively more responsive to the non-monetary treatments than households owning no latrine at baseline, since these households might need only minor improvements to reach or sustain hygienic status.

**Fig. 9:**
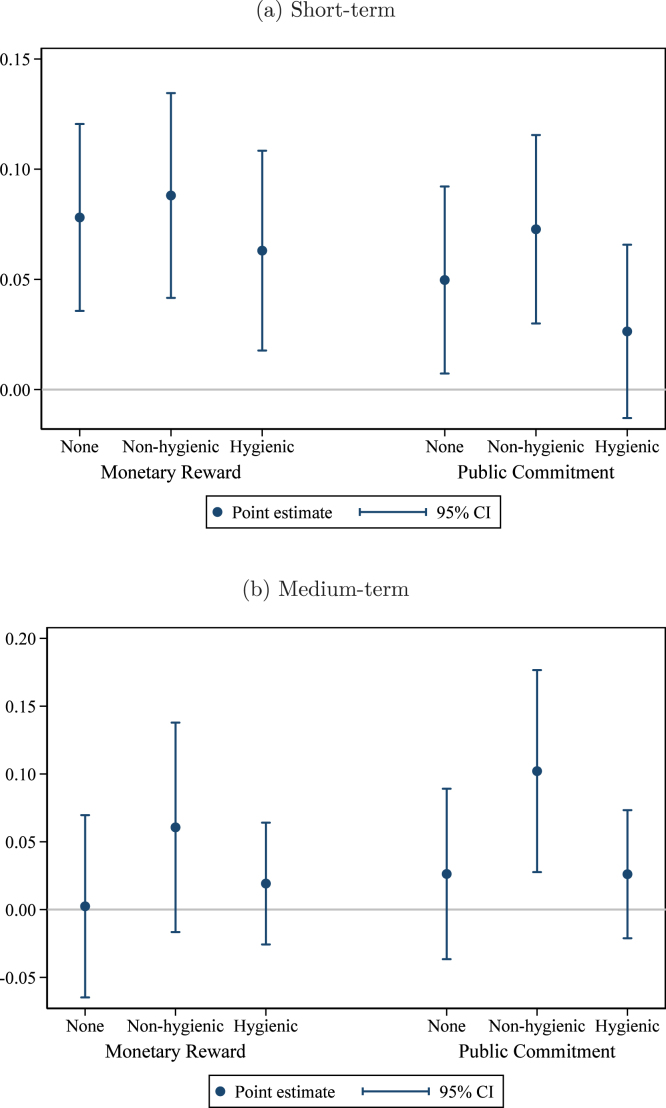
Effect on hygienic latrine ownership. By household’s baseline latrine ownership category. *Notes:* these graphs present estimated treatment effects of the Monetary Reward and Public Commitment treatments on ownership of a hygienic latrine by category of baseline latrine ownership. The top panel shows short-term effects and the bottom panel shows medium-term effects. The comparison group consists of households in villages receiving only the basic health intervention. Households in pure control villages are included to increase precision (estimates not reported). The regression controls for group-level baseline hygienic latrine ownership, group share of landless households, and union fixed effects. 95% confidence intervals use standard errors clustered at the village level (the level of randomization).

In the short term (Fig. 9(a), Appendix Table E3 columns (1) and (2)), the effects of both the monetary reward and public commitment treatment are similar across baseline ownership status categories. In the medium term (Fig. 9(b), Appendix Table E3 columns (3) and (4)), the point estimates indicate larger impacts among households owning a non-hygienic latrine at baseline, with borderline statistical significance for the public commitment treatment. That the public commitment treatment’s effect is sustained into the medium term suggests that modest improvements to existing latrines were more sustainable than major efforts to build a new, hygienic latrine quickly.

#### 5.2.3 Group characteristics

Ex ante, we proposed that the strength of the response to the reward treatment could vary with the group’s distance to the reward threshold at baseline. To test this hypothesis, we estimate

(3) $$y_{gv}=\beta_{1}\text{Incent}{}_{v}+\theta_{1}\left(\text{Incent}{}_{v}\times {\text{Dist}}_{gv}\right)$$

$$+\beta_{2}\text{Cert}{}_{v}+\theta_{2}\left(\text{Cert}{}_{v}\times {\text{Dist}}_{gv}\right)+\;\beta_{3}\text{Priv}{}_{v}+\beta_{4}\text{Publ}{}_{v}+\;\theta_{0}{\text{Dist}}_{gv}+\delta y_{0gv}+\gamma \text{ShareLandless}{}_{gv}+\;\beta_{0}\text{PureControl}{}_{v}+\varphi_{u}+{ɛ}_{gv}\text{,}$$

where ${\text{Dist}}_{gv}$ represents the distance between the group’s hygienic latrine ownership share at baseline and the next threshold above. For example, in any of Unions 2, 3, and 4, where the lower reward threshold was 33% and the upper reward threshold was 66%, a group with 20% hygienic latrine ownership at baseline would have ${\text{Dist}}_{gv}=0.13$, while a group with 50% hygienic latrine ownership at baseline would have ${\text{Dist}}_{gv}=0.16$.^19^ All other variables are as defined in Eq. (1) in the main text.

**Fig. 10:**
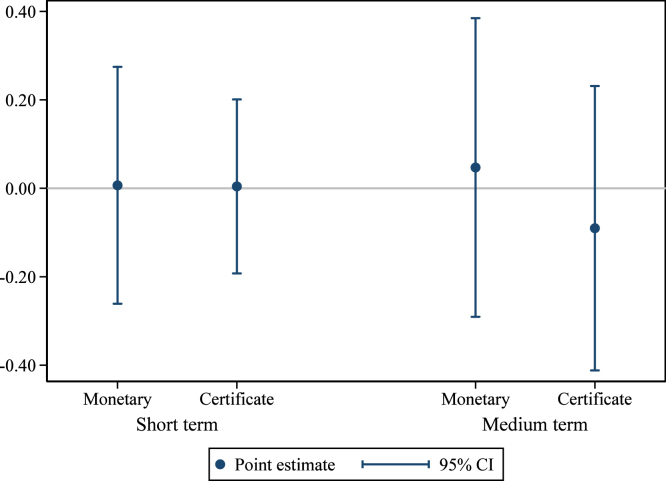
Treatment effect heterogeneity by distance to reward threshold hygienic latrine ownership. *Notes:* these graphs present estimated coefficients on interactions between the treatment indicated and the group’s distance at baseline to the nearest reward threshold above its current status. These correspond to $\theta_{1}$ and $\theta_{2}$ from Eq. (3) in the text. The dependent variable is the share of households in the group owning a hygienic latrine in either the short-term or medium-term followup. Groups above the higher threshold for their union are dropped. Groups in pure control villages are included to increase precision. The regression controls for group-level baseline hygienic latrine ownership, group share of landless households, and union fixed effects. 95% confidence intervals use standard errors clustered at the village level (the level of randomization).

These interactions are only estimated for the reward groups (monetary reward, reward certificate) because the thresholds were not relevant for the commitment treatments. The results are presented in Fig. 10.^20^ In no case is the estimated interaction term statistically significant, although all estimates are imprecise.

The parsimonious linear specification of Eq. (3) could mask a theoretically plausible nonlinear effect. For example, groups very near the threshold might respond only enough to get over the threshold, groups far from the threshold could be discouraged and respond very little, and the strongest effect on groups could be observed among groups at an intermediate distance from the threshold.

**Fig. 11:**
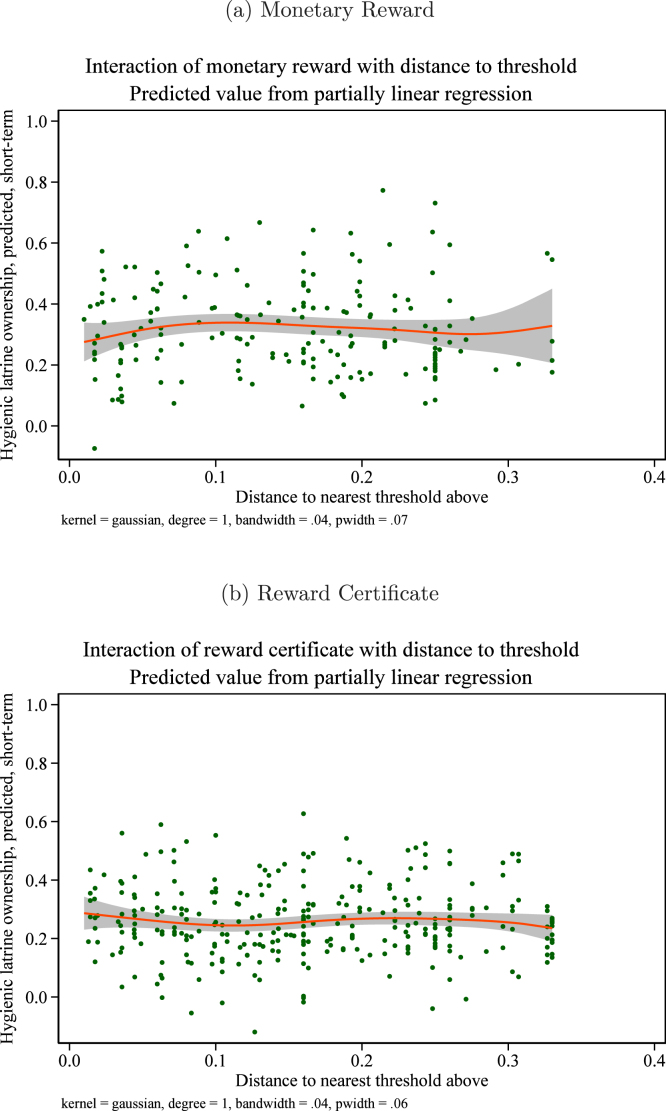
Interaction of reward treatments with distance to threshold. Short-term effects. *Notes:* these figures show the interaction between the treatment indicated with the group’s distance to the nearest threshold above at baseline, i.e., the response functions $f_{1}$ and $f_{2}$ in Eq. (4). The outcome variable is hygienic latrine ownership in the short term.

**Fig. 12:**
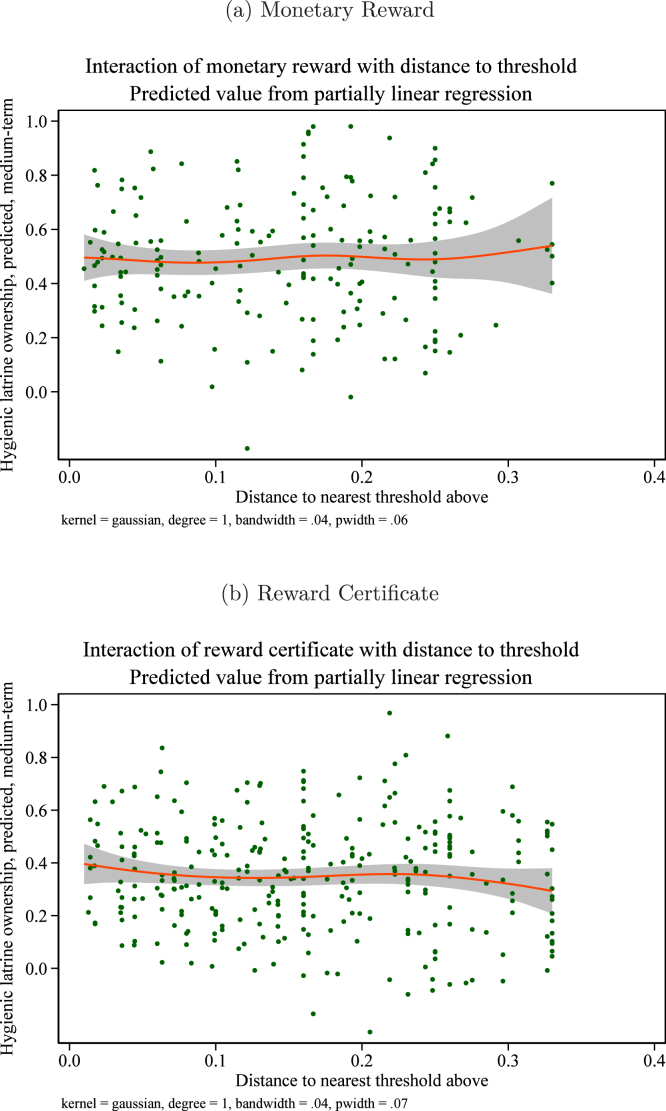
Interaction of reward treatments with distance to threshold. Medium-term effects. *Notes:* these figures show the interaction between the treatment indicated with the group’s distance to the nearest threshold above at baseline, i.e., the response functions $f_{1}$ and $f_{2}$ in Eq. (4). The outcome variable is hygienic latrine ownership in the medium term.

To allow for such nonlinearities, we estimate a semiparametric version of Eq. (3), as in

(4) $$y_{gv}=\beta_{1}\text{Incent}{}_{v}+f_{1}\left(\text{Incent}{}_{v}\times {\text{Dist}}_{gv}\right)$$

$$+\beta_{2}\text{Cert}{}_{v}+f_{2}\left(\text{Cert}{}_{v}\times {\text{Dist}}_{gv}\right)+\;\beta_{3}\text{Priv}{}_{v}+\beta_{4}\text{Publ}{}_{v}+\;\theta_{0}{\text{Dist}}_{gv}+\delta y_{0gv}+\gamma \text{ShareLandless}{}_{gv}+\;\beta_{0}\text{PureControl}{}_{v}+\varphi_{u}+{ɛ}_{gv}\text{,}$$

where, following Robinson (1988), the response functions $f_{1}$ and $f_{2}$ are estimated nonparametrically.^21^ Fig. 11, Fig. 11 plot results for short term hygienic latrine ownership and Fig. 12, Fig. 12 for medium term hygienic latrine ownership. In neither case do we see evidence of heterogeneous treatment effects.

Our second ex-ante hypothesis with respect to group-level characteristics was that treatment effects could vary with the baseline level of hygienic latrine ownership, especially if norms for sanitation that lead a group to have higher baseline hygienic latrine ownership enhance the effectiveness of treatments. An alternative possibility is that “holdout” households – i.e., households that do not own a hygienic latrine – in groups with high baseline ownership levels are especially set in their ways and unlikely to change their behavior. In this case, baseline ownership levels would be negatively associated with treatment effects.

To test this hypothesis, we estimate

(5) $$y_{gv}=\alpha_{0}+\alpha_{1}y_{0gv}$$

$$+\overset{4}{\underset{p=0}{\sum }}\beta_{p}\cdot 1\left\{1\text{Treat}{}_{v}=p\right\}+\overset{4}{\underset{p=0}{\sum }}\theta_{p}\cdot 1\left\{\text{Treat}{}_{v}=p\right\}\times y_{0gv}+\;\gamma \text{ShareLandless}{}_{gv}+\varphi_{u}+{ɛ}_{gv}\text{,}$$

where $y_{gv}$ is the outcome variable of interest for group g in village v, $y_{0gv}$ is the baseline level of the outcome variable for group g and all other variables are as defined in Eq. (2).^22^ The coefficient $\alpha_{1}$ represents the level effect of $y_{0gv}$, i.e., the association of $y_{0gv}$ with the outcome variable $y_{gv}$ in the comparison group, the coefficient $\beta_{p}$ represents the level effect of treatment p, i.e., the effect of treatment p on groups at the mean level of $y_{0gv}$, and the coefficient $\theta_{p}$ is the interaction between treatment p and characteristic $y_{0gv}$. We control for $\text{ShareLandless}{}_{gv}$ as a proxy for the overall economic resources of the group to attempt to isolate the norm-based mechanisms posited above.

**Fig. 13:**
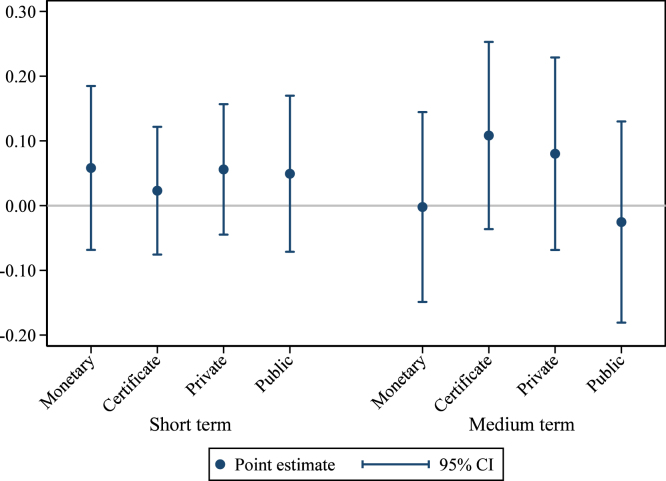
Treatment effect heterogeneity by baseline hygienic latrine ownership effect on hygienic latrine ownership. *Notes:* these graphs present estimated coefficients on interactions between the treatment indicated and the share of households in the group owning a hygienic latrine at baseline. These correspond to ${\left\{\theta_{p}\right\}}_{p=1}^{4}$ from Eq. (5). The dependent variable is share of households in the group with a hygienic latrine in either the short-term or medium-term followup. The comparison group consists of groups that received the meetings only treatment. Pure control villages are included as a separate category to enhance precision. The regression controls for group-level baseline hygienic latrine ownership (as a level effect), group share of landless households, and union fixed effects. 95% confidence intervals use standard errors clustered at the village level (the level of randomization).

The results are presented in Fig. 13.^23^ In the short term, there is some evidence in favor of a positive association, in that the point estimate of the interaction effect is positive across all four treatments, but the estimates are imprecise and no single estimate rises to statistical significance. The estimates are similarly imprecise in the medium term, and in this case there is no pattern in the sign of the point estimates.

Ex post, we conducted an exploratory analysis of the association between other group-level characteristics and the magnitude of treatment effects. We did not find strong evidence of differential effects with respect to group-level characteristics. This analysis and the results are presented in Appendix F. Responsiveness to our public commitment treatment is related to Cameron et al. (2019)’s finding that Indonesian communities with more social capital respond more strongly to a sanitation promotion treatment. To probe this further, we construct measures of community inter-connectedness using our social network surveys, but Tables F9 and F10 show that our treatment effects are no larger in more inter-connected communities.

## 6 Conclusion

Our research contributes to the technology adoption literature in development economics by drawing attention to the importance of inter-dependencies in decision-making. When each household’s investment decisions depends on others, that can lead to failures of collective action. We explore whether we can address an important public health externality by creating coordination schemes through simple social and financial group incentives that help communities overcome collective action failures. The two specific strategies we tested were creating joint liability by offering a joint monetary or non-monetary reward, and by encouraging community members to publicly commit to pursuing behaviors that would benefit community health in front of their neighbors.

We find that the monetary reward has the largest effect in the short term (3 months), increasing the share of households with hygienic latrines by 7.5 to 12.5 percentage points. The public commitment treatment leads to a 4.2 to 6.3 pp increase in the same period. The effect of the monetary reward faded in the medium term (15 months), while the effect of the public commitment treatment persisted. We find that this difference is explained by households in the public commitment treatment maintaining improvements in publicly visible components of the latrine. We find little evidence of heterogeneity in impacts with respect to group characteristics.

Public commitments are cheaper to implement than paying monetary rewards. The administrative costs of implementing the meetings required in our public commitment arm was about US$2 per household. This implies that each additional hygienic latrine investment generated by the public commitment arm (relative to pure control) came at an implementation cost of US$28.60 in the short term and US$24.40 in the medium term. In contrast, the implementation costs of the monetary reward treatment was US$4.29 per household (accounting for the actual rewards paid out), which translates to US$66.92 per additional hygienic latrine generated by that arm in the short term. Unsurprisingly given the treatment effects we report, public commitments are over twice as cost-effective as monetary rewards.^24^

The persistent increase in hygienic latrine ownership generated by our public commitment intervention is comparable in magnitude to the 6 percentage point increase in safely managed sanitation coverage in all of rural Bangladesh between 2015 and 2020 (WHO and UNICEF, 2022). The effect size is therefore large relative to observed improvements in sanitation in a country that has invested heavily in this sector. Providing direct latrine subsidies (Guiteras et al., 2015) increases the ownership of hygienic latrines by 15 percentage points (and “any latrine” by 13 percentage points), but those subsidies are much more expensive than encouraging public commitments and group interactions. And as we show above, much of our effect comes from latrine maintenance and investments in components (rather than new latrine construction), so effect sizes from the two studies are not directly comparable in all relevant dimensions.

Our results are immediately relevant for policymakers in South Asia and other developing countries struggling with the stubborn problem of low investment in improved sanitation and hygiene. They are also more broadly relevant for development economists studying the under-investment in a broader range of (seemingly beneficial) products, technologies and behaviors, including hand-washing and masks (Abaluck et al., 2021) that became especially relevant during the COVID-19 pandemic. We highlight decision inter-dependencies as a driving factor for adoption of product categories that may impose externalities on other members of society, or are strategic complements in investment. Our direct comparison of incentives and rewards (both monetary and in-kind) against public commitments contribute to an even broader literature in public economics on how personal and social incentives are shaped.

## Data Availability

Replication code and data are posted to the Harvard Dataverse at https://doi.org/10.7910/DVN/ACFSWO.

## Appendix A Supplementary data

MMC S1

## Footnotes

1 Union parishads or unions are the smallest rural administrative and local government units in Bangladesh.

2 Different sources define “hygienic” in different ways, and there are also other labels such as “improved” or “sanitary”. Conceptually, a hygienic latrine safely confines feces. For pour-flush latrines (the relevant type in our context), this typically requires slab, a water seal to block flies and other insects, and a sealed pit to store fecal matter for safe disposal (Hanchett et al., 2011). See Section SM2 of the Online Supplementary Materials for illustrations of the key components. Our precise definition of *hygienic* is below. A latrine that does not meet the criteria for hygienic is classified as a *non-hygienic* latrine. Households with a bucket, a “hanging latrine” (a platform over open land or water), or an open (uncovered) pit are classified as having no latrine. The detailed mapping from our survey instrument to these categories is provided in our Supplemental Materials, Section SM3.

3 The intervention protocol is provided in Section SM4 of the Online Supplementary Materials.

4 Households were also encouraged to ensure that no gaps existed between different latrines component that could compromise the ‘sealed’ nature of a pit. For single-pit latrines, this meant that there would not be any gap between the cover of the slab and the top-most ring. For offset latrines (involving multiple pits or a pit that is not situated directly below the latrine) there should not be any gap between the top-most ring and the pit cover. Moreover, although not perfectly observed (and therefore, not a strict requirement of a hygienic latrine) households were encouraged to install an adequate number of rings depending on the depth of the latrine pit.

5 US dollar equivalents at 75 BDT/USD, the approximate market exchange rate at the time.

6 This certificate was printed on thick glossy paper, so it could be displayed on an interior wall. However, no weatherproof frame was provided. As a result, it would be difficult for a household to display the certificate outdoors where it would be publicly visible.

7 This was the fourth round of followup data-collection for the project as a whole. We will refer to the baseline survey for the overall project as the “demand study baseline”. See Fig. 1 for the full project timeline.

8 As a robustness check, we add interactions with (de-meaned) values of the control variables. Following Lin (2013) and Gibbons et al. (2019), the level (non-interacted) terms are a more robust estimator of the average treatment effect in the presence of heterogeneity with respect to the control variables. The estimated level effects are similar to those we find in our main specification. See Appendix B for details.

9 As discussed in Muralidharan et al. (2020), in a factorial (interacted) design, the interpretation of coefficients in this “short” regression depends on priors about interaction effects. In the presence of interactions between treatments, $\text{Incent}{}_{v}$, for example, should be interpreted as the average effect of the incentive treatment in a context where some groups are receiving no other treatment, some the public commitment treatment, and some the private commitment treatment. It is important to note that the “short” regression was our pre-specified analysis. That is, Muralidharan et al. (2020) emphasize the incorrect inference that will result from a two-step analysis that first tests for the presence of interaction effects and then, if significant interaction effects are not detected, proceeds to the short regression. That was not our mode of analysis. Still, in retrospect, given our sample size constraints, it would have been preferable to design the experiment without treatment interactions. In Section G.1 of the Appendix, we present estimates using only the “single-treatment” villages, i.e., receiving only monetary reward, reward certificate, etc. The pattern of results is generally similar to those of our preferred specification, although the magnitude is somewhat reduced, suggesting the possibility of positive interaction between treatments. Similarly, when we estimate fully interacted factorial models in Section G.2 of the Appendix, we see some evidence of positive interactions, especially in the short term. However, as Muralidharan et al. (2020) point out, these tests have low power, so we view these results only as suggestive.

10 For full regression results, see Tables C1–C4 in Appendix C. The estimates plotted here correspond to our preferred specification, i.e., column (2) of the regression tables.

11 We prioritize our direct observation of the latrine condition as the outcome variable rather than self-reported open defecation, because data on these short-term outcomes were collected as part of the end-of-intervention assessment and therefore even more prone to bias than usual, and especially so in the rewards treatments.

12 We cannot distinguish clearly between an absolute fading of the program effect versus a catch-up by the comparison group. The share of households in the comparison group (meetings-only treatment villages) owning a hygienic latrine increased from 0.45 to 0.54 between the short-term and medium-term followups, and a similar pattern was observed in the pure control group (0.40 to 0.53). However, these measures are not exactly comparable across rounds both because of seasonality and some inconsistencies in the survey.

13 Full regression results reported in Tables C1–C5 of Appendix C.

14 We were not able to collect reliable data on these outcomes in the medium term. That survey took place roughly a year later, and we found that households struggled to remember the exact timing of different investments. This was especially difficult for households in the pure control and meetings only arms, since they did not have the reward or recognition event as a reference point to anchor their memories.

15 In these figures, we focus on estimates of the Monetary Reward and Public Commitment treatments. For full regression results and estimated differences between treatments, see Tables D1 and D2.

16 For full regression results and formal tests of differences between treatment arms, see Tables D3 and D4.

17 For full regression results and formal tests of differences between treatment arms, see Table D5.

18 For comparison with the group-level results, we estimate Eq. (2) with no household characteristics or interactions in Appendix Table E1. The results are very close to the corresponding group-level estimates in Table 3.

19 In the regressions, we de-mean ${\text{Dist}}_{gv}$, so the level effect $\beta_{p}$ represents the effect of treatment p on groups with the mean level of ${\text{Dist}}_{gv}$ (Wainer, 2000).

20 For full regression results, see Table F1.

21 We gratefully acknowledge Verardi and Debarsy (2012)’s Stata implementation of the Robinson estimator.

22 As with Eq. (2), we de-mean $y_{0gv}$, so the level terms $\beta_{p}$ represent the effect of treatment p on groups with the mean level of $y_{0gv}$.

23 See Table F2 for full regression results.

24 See Section SM6 for details of these calculations.
