# Supplementary material for: Social and financial incentives for overcoming a collective action problem
Source: J Dev Econ. 2023 May;162:103072. doi: 10.1016/j.jdeveco.2023.103072 (PMC10115903; doi:10.1016/j.jdeveco.2023.103072)
Supplement: MMC S1 [file mmc1.pdf]

Social and Financial Incentives for Overcoming a Collective Action Problem

Appendix Tables and Figures

M. Mehrab Bakhtiar*
IFPRI

Raymond Guiteras
North Carolina State University

James Levinsohn
Yale University

Ahmed Mushfiq Mobarak
Yale University

January 2023

*Email: m.bakhtiar@cgiar.org, rpguiter@ncsu.edu, james.levinsohn@yale.edu, ahmed.mobarak@yale.edu.

A Effect of Common, Meetings-Only Treatment **A-1**

B Robustness Checks for Main Outcomes of Interest **B-1**

C Estimated Effects on Secondary Outcomes **C-1**

D Household Investments and Behaviors **D-1**

E Interaction with Household Characteristics **E-1**

F Interaction with Group Characteristics **F-1**

G Single Treatment Villages and Fully Interacted Model **G-1**

G.1 Single-Treatment Village Estimates G-1

G.2 Fully Interacted Model G-1

Appendix References

A Effect of Common, Meetings-Only Treatment

This appendix provides estimates of the effect of the basic information treatment relative to the pure control group. This effect is captured by $-\beta_0$ in Equation 1 in the main text, reproduced here for convenience:

$$y_{gv} = \beta_1 \text{Incent}_v + \beta_2 \text{Cert}_v + \beta_3 \text{Priv}_v + \beta_4 \text{Publ}_v \quad (1)$$

$$+ \delta y_{0gv} + \gamma \text{ShareLandless}_{gv} + \beta_0 \text{PureControl}_v + \varphi_u + \varepsilon_{gv}$$

In each table, estimates of short-term effects are presented in columns (1) and (2), with medium-term effects in columns (3) and (4). The meetings-only treatment has null effects on nearly all of our main outcomes of interest in both the short and medium term. The one exception is the economically large and borderline statistically significant ($p < 0.10$) effect on household self-reported open defecation in the medium term (Table A6). However, note that this is not reflected in the surveyor assessment of open defecation in the same period (Table A5), nor in any of the endline measures of access or ownership (Tables A1, A2, A3, A4), and so this seems likely to be the result of social desirability bias rather than a true change in behavior.

Table A1: Hygienic Latrine Ownership

	Short term		Medium term	
	(1)	(2)	(3)	(4)
Meetings Only	-0.015	-0.014	-0.025	-0.025
	(0.041)	(0.023)	(0.034)	(0.021)
	[-0.075, 0.113]	[-0.041, 0.071]	[-0.047, 0.103]	[-0.020, 0.071]
Baseline share owning hyg. lat.		0.709***		0.513***
		(0.022)		(0.036)
Share of households landless		-0.083***		-0.089***
		(0.017)		(0.032)
Union FEs	Yes	Yes	Yes	Yes
Number of groups	377	376	377	376
Number of villages	31	31	31	31
Omitted category mean	0.399	0.401	0.540	0.543
Omitted category S.D.	(0.240)	(0.240)	(0.249)	(0.247)

Notes: the dependent variable is the share of households in the group owning a hygienic latrine. Columns (1) and (2) report short-term effects (at the time of assessment); columns (3) and (4) report medium-term effects (12-15 months after assessment). Observations (groups) are weighted by the number of households. The sample consists of all villages. This table presents only the effect of the meetings-only treatment relative to pure controls; effects of the other treatments are reported in the main text. The sample sizes reported here are groups and villages in the meetings-only treatment and pure controls. The omitted category consists of groups in pure control villages. Standard errors clustered at the village level. Standard errors clustered at the village level in parentheses. Wild cluster bootstrap (9,999 repetitions, Webb weights) 95% confidence intervals, resampling at the village level, in brackets for the coefficients of interest. * $p < 0.10$, ** $p < 0.05$, *** $p < 0.01$.

Table A2: Hygienic Latrine Access

	Short term		Medium term	
	(1)	(2)	(3)	(4)
Meetings Only	-0.023	-0.027	-0.034	-0.037
	(0.045)	(0.026)	(0.037)	(0.025)
	[-0.072, 0.129]	[-0.035, 0.089]	[-0.044, 0.118]	[-0.018, 0.093]
Hygienic latrine access (group share, R4)		0.691***		0.443***
		(0.025)		(0.038)
Share of households landless		-0.096***		-0.083**
		(0.022)		(0.033)
Union FEs	Yes	Yes	Yes	Yes
Number of groups	377	377	377	377
Number of villages	31	31	31	31
Omitted category mean	0.484	0.484	0.636	0.636
Omitted category S.D.	(0.268)	(0.268)	(0.253)	(0.253)

Notes: the dependent variable is the share of households in the group with access to a hygienic latrine. Columns (1) and (2) report short-term effects (at the time of assessment); columns (3) and (4) report medium-term effects (12-15 months after assessment). Observations (groups) are weighted by the number of households. The sample consists of all villages. This table presents only the effect of the meetings-only treatment relative to pure controls; effects of the other treatments are reported in the main text. The sample sizes reported here are groups and villages in the meetings-only treatment and pure controls. The omitted category consists of groups in pure control villages. Standard errors clustered at the village level. Standard errors clustered at the village level in parentheses. Wild cluster bootstrap (9,999 repetitions, Webb weights) 95% confidence intervals, resampling at the village level, in brackets for the coefficients of interest. * $p < 0.10$, ** $p < 0.05$, *** $p < 0.01$.

Table A3: Any Latrine Ownership

	Short term		Medium term	
	(1)	(2)	(3)	(4)
Meetings Only	-0.005	0.019	0.001	0.017
	(0.031)	(0.020)	(0.024)	(0.020)
	[-0.061, 0.073]	[-0.061, 0.029]	[-0.052, 0.052]	[-0.060, 0.026]
Any latrine ownership (group share, R4)		0.682***		0.513***
		(0.031)		(0.043)
Share of households landless		-0.096***		-0.129***
		(0.021)		(0.026)
Union FEs	Yes	Yes	Yes	Yes
Number of groups	377	376	377	376
Number of villages	31	31	31	31
Omitted category mean	0.666	0.669	0.768	0.771
Omitted category S.D.	(0.229)	(0.225)	(0.203)	(0.198)

Notes: the dependent variable is the share of households in the group owning any latrine. Columns (1) and (2) report short-term effects (at the time of assessment); columns (3) and (4) report medium-term effects (12-15 months after assessment). Observations (groups) are weighted by the number of households. The sample consists of all villages. This table presents only the effect of the meetings-only treatment relative to pure controls; effects of the other treatments are reported in the main text. The sample sizes reported here are groups and villages in the meetings-only treatment and pure controls. The omitted category consists of groups in pure control villages. Standard errors clustered at the village level. Standard errors clustered at the village level in parentheses. Wild cluster bootstrap (9,999 repetitions, Webb weights) 95% confidence intervals, resampling at the village level, in brackets for the coefficients of interest. * $p < 0.10$, ** $p < 0.05$, *** $p < 0.01$.

Table A4: Any Latrine Access

	Short term		Medium term	
	(1)	(2)	(3)	(4)
Meetings Only	-0.018	-0.004	-0.009	-0.000
	(0.030)	(0.014)	(0.023)	(0.015)
	[-0.044, 0.081]	[-0.027, 0.035]	[-0.042, 0.058]	[-0.035, 0.033]
Any latrine access (group share, R4)		0.680***		0.456***
		(0.032)		(0.045)
Share of households landless		-0.102***		-0.078***
		(0.022)		(0.025)
Union FEs	Yes	Yes	Yes	Yes
Number of groups	377	377	377	377
Number of villages	31	31	31	31
Omitted category mean	0.850	0.850	0.916	0.916
Omitted category S.D.	(0.208)	(0.208)	(0.153)	(0.153)

Notes: the dependent variable is the share of households in the group with access to any latrine. Columns (1) and (2) report short-term effects (at the time of assessment); columns (3) and (4) report medium-term effects (12-15 months after assessment). Observations (groups) are weighted by the number of households. The sample consists of all villages. This table presents only the effect of the meetings-only treatment relative to pure controls; effects of the other treatments are reported in the main text. The sample sizes reported here are groups and villages in the meetings-only treatment and pure controls. The omitted category consists of groups in pure control villages. Standard errors clustered at the village level. Standard errors clustered at the village level in parentheses. Wild cluster bootstrap (9,999 repetitions, Webb weights) 95% confidence intervals, resampling at the village level, in brackets for the coefficients of interest. * $p < 0.10$, ** $p < 0.05$, *** $p < 0.01$.

Table A5: Open Defecation – Surveyor Assessment

	Short term		Medium term	
	(1)	(2)	(3)	(4)
Meetings Only	0.018	-0.000	0.009	-0.003
	(0.030)	(0.012)	(0.023)	(0.014)
	[-0.081, 0.045]	[-0.024, 0.025]	[-0.058, 0.043]	[-0.029, 0.034]
Open defecation (group share, R4)		0.760***		0.504***
		(0.028)		(0.046)
Share of households landless		0.050**		0.046*
		(0.019)		(0.025)
Union FEs	Yes	Yes	Yes	Yes
Number of groups	377	377	377	377
Number of villages	31	31	31	31
Omitted category mean	0.150	0.150	0.084	0.084
Omitted category S.D.	(0.208)	(0.208)	(0.153)	(0.153)

Notes: the dependent variable is the share of households primarily practicing OD. Columns (1) and (2) report short-term effects (at the time of assessment); columns (3) and (4) report medium-term effects (12-15 months after assessment). Observations (groups) are weighted by the number of households. The sample consists of all villages. This table presents only the effect of the meetings-only treatment relative to pure controls; effects of the other treatments are reported in the main text. The sample sizes reported here are groups and villages in the meetings-only treatment and pure controls. The omitted category consists of groups in pure control villages. Standard errors clustered at the village level. Standard errors clustered at the village level in parentheses. Wild cluster bootstrap (9,999 repetitions, Webb weights) 95% confidence intervals, resampling at the village level, in brackets for the coefficients of interest. * $p < 0.10$, ** $p < 0.05$, *** $p < 0.01$.

Table A6: Medium Term OD – Household Self-Report

	(1)	(2)
Meetings Only	-0.171*	-0.162*
	(0.089)	(0.087)
	[-0.050, 0.368]	[-0.051, 0.353]
Open defecation (group share, R4)		0.414***
		(0.052)
Share of households landless		0.143***
		(0.040)
Union FEs	Yes	Yes
Number of groups	377	377
Number of villages	31	31
Omitted category mean	0.469	0.469
Omitted category S.D.	(0.355)	(0.355)

Notes: the dependent variable is the share of households regularly practicing OD in the medium term (12-15 months after assessment). Observations (groups) are weighted by the number of households. The sample consists of all villages. This table presents only the effect of the meetings-only treatment relative to pure controls; effects of the other treatments are reported in the main text. The sample sizes reported here are groups and villages in the meetings-only treatment and pure controls. The omitted category consists of groups in pure control villages. Standard errors clustered at the village level. Standard errors clustered at the village level in parentheses. Wild cluster bootstrap (9,999 repetitions, Webb weights) 95% confidence intervals, resampling at the village level, in brackets for the coefficients of interest. * $p < 0.10$, ** $p < 0.05$, *** $p < 0.01$.

B Robustness Checks for Main Outcomes of Interest

This appendix reports two sets of robustness checks for our estimates of treatment effects on the main outcomes of interest: hygienic latrine ownership in the short and medium term.

The first robustness check consists of re-estimating Equation 1 from the main text, but assigning each group equal weight (i.e., without weighting groups by the number of households). The estimates, reported in Table B1, are virtually unchanged from those of the main text (Table 3).

The second robustness check consists of specifications augmenting Equation 1 from the main text by interacting the treatment dummies with demeaned values of the control variables. As argued by Lin (2013) and Gibbons et al. (2019), the level (non-interacted) terms are a more robust estimator of the average treatment effect in the presence of heterogeneity with respect to the control variables. Specifically, we estimate

$$\begin{aligned} y_{gv} = & \sum_{t=0}^{4} \beta_t \{\text{Treatment}_v = t\} \\ & + \sum_{t=0}^{4} \delta_t \{\text{Treatment}_v = t\} \times y_{0gv} + \sum_{t=0}^{4} \gamma_t \{\text{Treatment}_v = t\} \times \text{ShareLandless}_{gv} \\ & + \delta_L y_{0gv} + \gamma_L \text{ShareLandless}_{gv} \\ & + \varphi_u + \varepsilon_{gv}, \end{aligned} \tag{B-1}$$

where $t = 0, \ldots, 4$ indexes treatments (PureControl, Incent, Cert, Priv, Publ), and each treatment is interacted with (demeaned values of) the control variables y_{0gv} and $\text{ShareLandless}_{gv}$. The coefficients β_t on the non-interacted treatment term represent average treatment effects. As in the main text, the common, meetings-only treatment is the omitted category so estimates are relative to that group.

We present results for our main outcome of interest, hygienic latrine ownership, in Table B2 and Figure B1. Here, we report the non-interacted coefficients (i.e., $\{\beta_t\}$ from Equation B-1), representing the average treatment effects; we discuss interactions in Section 5.2. The results are generally similar to those in the main text. The main difference to note is that the estimated difference between the short-run effects of the monetary reward and private commitment treatments has the same magnitude but is no longer significant at the 10% level ($p = 0.101$).

Table B1: Program Effects: Hygienic Latrine Ownership
Unweighted Regressions

	Short term		Medium term	
	(1)	(2)	(3)	(4)
Monetary Reward	0.125***	0.079***	0.048	0.013
	(0.035)	(0.016)	(0.031)	(0.020)
	[0.046, 0.203]	[0.044, 0.111]	[-0.019, 0.116]	[-0.030, 0.056]
Reward Certificate	0.048	0.012	0.045	0.018
	(0.040)	(0.012)	(0.038)	(0.023)
	[-0.050, 0.142]	[-0.015, 0.039]	[-0.044, 0.132]	[-0.033, 0.068]
Private Commitment	0.012	0.010	0.017	0.016
	(0.040)	(0.012)	(0.041)	(0.025)
	[-0.078, 0.106]	[-0.018, 0.037]	[-0.082, 0.113]	[-0.046, 0.071]
Public Commitment	0.067*	0.045***	0.080***	0.062***
	(0.037)	(0.016)	(0.029)	(0.017)
	[-0.016, 0.150]	[0.010, 0.080]	[0.019, 0.140]	[0.027, 0.098]
Baseline share owning hyg. lat.		0.712***		0.518***
		(0.022)		(0.037)
Share of households landless		-0.083***		-0.090***
		(0.018)		(0.033)
Union FEs	Yes	Yes	Yes	Yes
Diff.: Monetary – Public	0.058	0.034	-0.032	-0.049
	(0.047)	(0.020)	(0.042)	(0.031)
p-value	0.216	0.097	0.448	0.121
Diff.: Monetary – Certificate	0.076	0.067	0.003	-0.005
	(0.040)	(0.016)	(0.033)	(0.019)
p-value	0.058	0.000	0.935	0.795
Diff.: Public – Private	0.055	0.035	0.063	0.047
	(0.041)	(0.015)	(0.037)	(0.022)
p-value	0.180	0.023	0.095	0.040
Number of groups	1,236	1,235	1,235	1,234
Number of villages	107	107	107	107
Omitted category mean	0.451	0.451	0.544	0.544
Omitted category S.D.	(0.189)	(0.189)	(0.255)	(0.255)

Notes: the dependent variable is the share of households in the group owning a hygienic latrine. Columns (1) and (2) report short-term effects (at the time of assessment); columns (3) and (4) report medium-term effects (12-15 months after assessment). Observations (groups) are unweighted. The comparison group consists of groups that received the meetings only treatment. Pure control villages are included as a separate category to enhance precision. Standard errors clustered at the village level. Standard errors clustered at the village level in parentheses. Wild cluster bootstrap (9,999 repetitions, Webb weights) 95% confidence intervals, resampling at the village level, in brackets for the coefficients of interest. * $p < 0.10$, ** $p < 0.05$, *** $p < 0.01$.

Table B2: Program Effects: Hygienic Latrine Ownership
Interact Treatment with De-Meaned Control Variables

	Short term		Medium term	
	(1)	(2)	(3)	(4)
Monetary Reward	0.125***	0.075***	0.047	0.012
	(0.034)	(0.015)	(0.030)	(0.021)
	[0.052, 0.200]	[0.042, 0.108]	[-0.017, 0.111]	[-0.031, 0.056]
Reward Certificate	0.044	0.008	0.043	0.015
	(0.037)	(0.012)	(0.035)	(0.021)
	[-0.047, 0.130]	[-0.019, 0.035]	[-0.040, 0.124]	[-0.033, 0.061]
Private Commitment	0.008	0.007	0.013	0.009
	(0.038)	(0.012)	(0.039)	(0.024)
	[-0.076, 0.098]	[-0.019, 0.034]	[-0.079, 0.103]	[-0.046, 0.063]
Public Commitment	0.063*	0.043***	0.072**	0.055***
	(0.036)	(0.015)	(0.028)	(0.018)
	[-0.017, 0.143]	[0.009, 0.078]	[0.012, 0.132]	[0.018, 0.093]
Baseline share owning hyg. lat.		0.694***		0.451***
		(0.053)		(0.084)
Share of households landless		-0.016		-0.152**
		(0.040)		(0.066)
Union FEs	Yes	Yes	Yes	Yes
Diff.: Monetary – Public	0.062	0.033	-0.025	-0.043
	(0.046)	(0.020)	(0.042)	(0.030)
p-value	0.182	0.101	0.544	0.161
Diff.: Monetary – Certificate	0.081	0.068	0.004	-0.003
	(0.039)	(0.015)	(0.032)	(0.019)
p-value	0.038	0.000	0.899	0.892
Diff.: Public – Private	0.055	0.035	0.059	0.046
	(0.038)	(0.014)	(0.036)	(0.021)
p-value	0.156	0.013	0.098	0.030
Number of groups	1,236	1,235	1,235	1,234
Number of villages	107	107	107	107
Omitted category mean	0.451	0.451	0.544	0.544
Omitted category S.D.	(0.189)	(0.189)	(0.255)	(0.255)

Notes: the dependent variable is the share of households in the group owning a hygienic latrine. Columns (1) and (2) report short-term effects (at the time of assessment); columns (3) and (4) report medium-term effects (12-15 months after assessment). Observations (groups) are weighted by the number of households. The comparison group consists of groups that received the meetings only treatment. Pure control villages are included as a separate category to enhance precision. Standard errors clustered at the village level. Standard errors clustered at the village level in parentheses. Wild cluster bootstrap (9,999 repetitions, Webb weights) 95% confidence intervals, resampling at the village level, in brackets for the coefficients of interest. * $p < 0.10$, ** $p < 0.05$, *** $p < 0.01$.

Figure B1: Program Effects: Hygienic Latrine Ownership
Interact Treatment with De-Meaned Control Variables

(a) Short-term

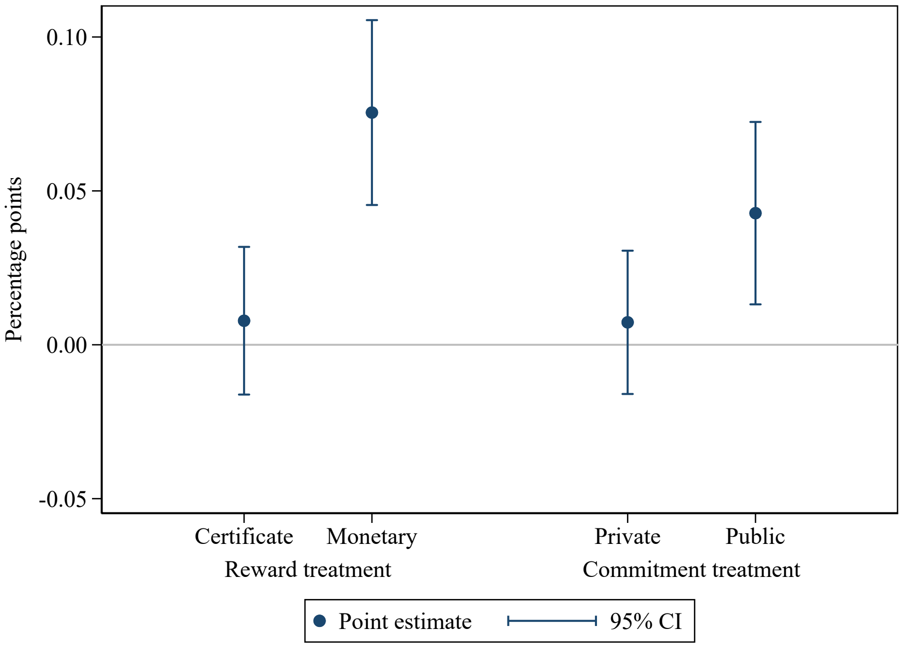

(b) Medium-term

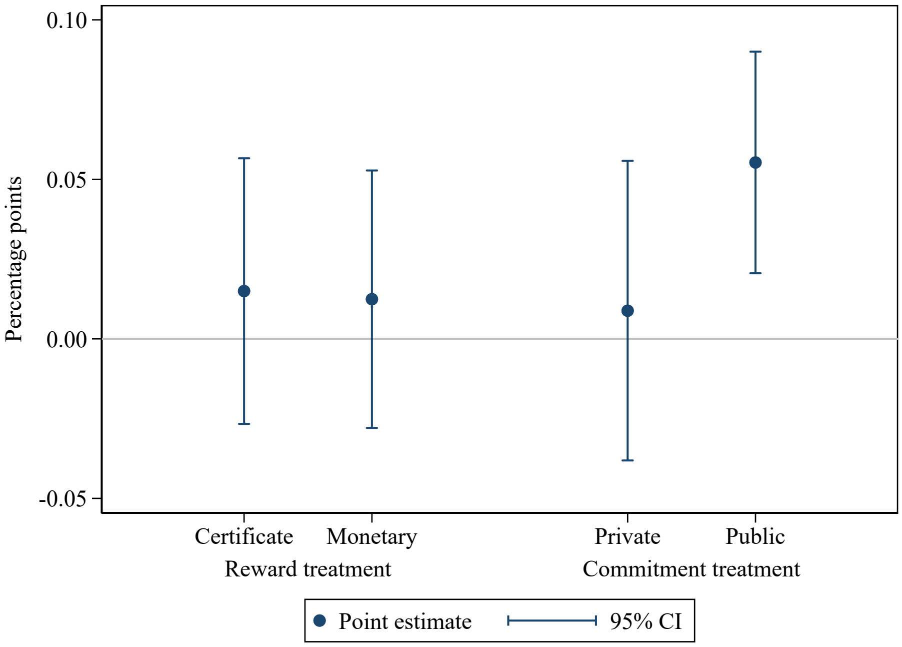

Notes: this graph presents estimated treatment effects of the interventions on the share of households in the group owning a hygienic latrine. Panel (a) presents effects in the short term (at the time of assessment); panel (b) in the medium term (12-15 months after assessment). The regression controls for the baseline level of the outcome variable, the share of households in the group that are landless, and union fixed effects. Following Lin (2013), the control variables are de-meaned and interacted with treatment indicators. This figure plots the non-interacted treatment coefficients, representing average treatment effects. Observations (groups) are weighted by the number of households. The comparison group consists of groups that received the meetings only treatment. Pure control villages are included as a separate category to enhance precision. 95% confidence intervals use standard errors clustered at the village level (the level of randomization).

C Estimated Effects on Secondary Outcomes

This appendix presents estimated treatment effects in the short- and medium-term on secondary outcomes of interest, namely:

- Hygienic Latrine Access: Table C1
- Any Latrine Ownership: Table C2
- Any Latrine Access: Table C3
- Open Defecation – Surveyor Assessment: Table C4
- Open Defecation – Household Self-Report: Table C5 (medium-term only)

The estimation equation is given by Equation 1 in the main text.

Table C1: Program Effects: Hygienic Latrine Access

	Short term		Medium term	
	(1)	(2)	(3)	(4)
Monetary Reward	0.123***	0.076***	0.030	-0.000
	(0.037)	(0.018)	(0.034)	(0.023)
	[0.045, 0.202]	[0.037, 0.114]	[-0.043, 0.105]	[-0.050, 0.049]
Reward Certificate	0.052	0.008	0.045	0.017
	(0.044)	(0.016)	(0.043)	(0.029)
	[-0.057, 0.152]	[-0.028, 0.045]	[-0.060, 0.145]	[-0.049, 0.081]
Private Commitment	0.021	0.028*	0.021	0.025
	(0.045)	(0.015)	(0.047)	(0.032)
	[-0.076, 0.124]	[-0.006, 0.060]	[-0.091, 0.131]	[-0.054, 0.098]
Public Commitment	0.071	0.052**	0.088**	0.075***
	(0.043)	(0.021)	(0.034)	(0.021)
	[-0.024, 0.167]	[0.005, 0.100]	[0.016, 0.160]	[0.030, 0.122]
Hygienic latrine access (group share, R4)		0.691***		0.443***
		(0.025)		(0.038)
Share of households landless		-0.096***		-0.083**
		(0.022)		(0.033)
Union FEs	Yes	Yes	Yes	Yes
Diff.: Monetary – Public	0.052	0.024	-0.058	-0.075
	(0.055)	(0.026)	(0.050)	(0.035)
p-value	0.343	0.348	0.245	0.035
Diff.: Monetary – Certificate	0.071	0.068	-0.015	-0.017
	(0.045)	(0.020)	(0.038)	(0.024)
p-value	0.116	0.001	0.695	0.469
Diff.: Public – Private	0.049	0.024	0.067	0.050
	(0.043)	(0.020)	(0.042)	(0.029)
p-value	0.253	0.225	0.111	0.086
Number of groups	1,236	1,236	1,235	1,235
Number of villages	107	107	107	107
Omitted category mean	0.528	0.528	0.627	0.627
Omitted category S.D.	(0.218)	(0.218)	(0.278)	(0.278)

Notes: the dependent variable is the share of households in the group with access to a hygienic latrine. Columns (1) and (2) report short-term effects (at the time of assessment); columns (3) and (4) report medium-term effects (12-15 months after assessment). Observations (groups) are weighted by the number of households. The comparison group consists of groups that received the meetings only treatment. Pure control villages are included as a separate category to enhance precision. Standard errors clustered at the village level. Standard errors clustered at the village level in parentheses. Wild cluster bootstrap (9,999 repetitions, Webb weights) 95% confidence intervals, resampling at the village level, in brackets for the coefficients of interest. * $p < 0.10$, ** $p < 0.05$, *** $p < 0.01$.

Table C2: Program Effects: Any Latrine Ownership

	Short term		Medium term	
	(1)	(2)	(3)	(4)
Monetary Reward	0.065***	0.028*	0.023	-0.004
	(0.023)	(0.015)	(0.021)	(0.016)
	[0.017, 0.113]	[-0.002, 0.061]	[-0.022, 0.068]	[-0.038, 0.032]
Reward Certificate	0.029	0.004	0.029	0.009
	(0.025)	(0.012)	(0.020)	(0.016)
	[-0.031, 0.087]	[-0.021, 0.028]	[-0.017, 0.071]	[-0.024, 0.043]
Private Commitment	-0.007	-0.006	-0.008	-0.008
	(0.027)	(0.012)	(0.022)	(0.016)
	[-0.066, 0.053]	[-0.031, 0.019]	[-0.060, 0.039]	[-0.041, 0.028]
Public Commitment	0.023	0.002	0.008	-0.009
	(0.023)	(0.014)	(0.017)	(0.015)
	[-0.027, 0.073]	[-0.030, 0.033]	[-0.028, 0.044]	[-0.041, 0.022]
Any latrine ownership (group share, R4)		0.682***		0.513***
		(0.031)		(0.043)
Share of households landless		-0.096***		-0.129***
		(0.021)		(0.026)
Union FEs	Yes	Yes	Yes	Yes
Diff.: Monetary – Public	0.042	0.026	0.015	0.005
	(0.033)	(0.020)	(0.025)	(0.023)
p-value	0.209	0.201	0.560	0.842
Diff.: Monetary – Certificate	0.035	0.025	-0.006	-0.013
	(0.024)	(0.014)	(0.021)	(0.014)
p-value	0.142	0.076	0.774	0.353
Diff.: Public – Private	0.030	0.008	0.016	-0.001
	(0.026)	(0.014)	(0.021)	(0.016)
p-value	0.254	0.581	0.447	0.944
Number of groups	1,236	1,235	1,235	1,234
Number of villages	107	107	107	107
Omitted category mean	0.713	0.713	0.782	0.782
Omitted category S.D.	(0.204)	(0.204)	(0.217)	(0.217)

Notes: the dependent variable is the share of households in the group owning any latrine. Columns (1) and (2) report short-term effects (at the time of assessment); columns (3) and (4) report medium-term effects (12-15 months after assessment). Observations (groups) are weighted by the number of households. The comparison group consists of groups that received the meetings only treatment. Pure control villages are included as a separate category to enhance precision. Standard errors clustered at the village level. Standard errors clustered at the village level in parentheses. Wild cluster bootstrap (9,999 repetitions, Webb weights) 95% confidence intervals, resampling at the village level, in brackets for the coefficients of interest. * $p < 0.10$, ** $p < 0.05$, *** $p < 0.01$.

Table C3: Program Effects: Any Latrine Access

	Short term		Medium term	
	(1)	(2)	(3)	(4)
Monetary Reward	0.038	0.018	0.006	-0.007
	(0.026)	(0.014)	(0.022)	(0.017)
	[-0.016, 0.094]	[-0.014, 0.051]	[-0.043, 0.055]	[-0.044, 0.030]
Reward Certificate	0.042	0.011	0.028	0.007
	(0.026)	(0.012)	(0.022)	(0.013)
	[-0.019, 0.098]	[-0.014, 0.036]	[-0.023, 0.075]	[-0.022, 0.036]
Private Commitment	-0.016	-0.004	-0.009	-0.001
	(0.027)	(0.013)	(0.024)	(0.015)
	[-0.077, 0.042]	[-0.031, 0.025]	[-0.067, 0.044]	[-0.034, 0.031]
Public Commitment	-0.002	-0.010	0.003	-0.002
	(0.025)	(0.012)	(0.018)	(0.013)
	[-0.055, 0.050]	[-0.035, 0.016]	[-0.035, 0.041]	[-0.029, 0.025]
Any latrine access (group share, R4)		0.680***		0.456***
		(0.032)		(0.045)
Share of households landless		-0.102***		-0.078***
		(0.022)		(0.025)
Union FEs	Yes	Yes	Yes	Yes
Diff.: Monetary – Public	0.040	0.028	0.003	-0.005
	(0.039)	(0.019)	(0.030)	(0.024)
p-value	0.299	0.159	0.914	0.833
Diff.: Monetary – Certificate	-0.003	0.007	-0.021	-0.014
	(0.027)	(0.015)	(0.025)	(0.017)
p-value	0.905	0.655	0.398	0.390
Diff.: Public – Private	0.014	-0.006	0.012	-0.001
	(0.027)	(0.013)	(0.023)	(0.014)
p-value	0.606	0.674	0.597	0.934
Number of groups	1,236	1,236	1,235	1,235
Number of villages	107	107	107	107
Omitted category mean	0.868	0.868	0.918	0.918
Omitted category S.D.	(0.195)	(0.195)	(0.188)	(0.188)

Notes: the dependent variable is the share of households in the group with access to any latrine. Columns (1) and (2) report short-term effects (at the time of assessment); columns (3) and (4) report medium-term effects (12-15 months after assessment). Observations (groups) are weighted by the number of households. The comparison group consists of groups that received the meetings only treatment. Pure control villages are included as a separate category to enhance precision. Standard errors clustered at the village level. Standard errors clustered at the village level in parentheses. Wild cluster bootstrap (9,999 repetitions, Webb weights) 95% confidence intervals, resampling at the village level, in brackets for the coefficients of interest. * $p < 0.10$, ** $p < 0.05$, *** $p < 0.01$.

Table C4: Program Effects: Open Defecation
Based on surveyor assessment of household's primary latrine

	Short term		Medium term	
	(1)	(2)	(3)	(4)
Monetary Reward	-0.038	-0.005	-0.006	0.015
	(0.026)	(0.012)	(0.022)	(0.015)
	[-0.092, 0.015]	[-0.032, 0.021]	[-0.054, 0.042]	[-0.019, 0.048]
Reward Certificate	-0.042	-0.004	-0.028	-0.003
	(0.026)	(0.010)	(0.022)	(0.013)
	[-0.097, 0.019]	[-0.026, 0.015]	[-0.074, 0.022]	[-0.031, 0.026]
Private Commitment	0.016	0.001	0.009	-0.001
	(0.027)	(0.010)	(0.024)	(0.014)
	[-0.043, 0.077]	[-0.023, 0.023]	[-0.043, 0.067]	[-0.031, 0.031]
Public Commitment	0.002	-0.006	-0.003	-0.008
	(0.025)	(0.010)	(0.018)	(0.011)
	[-0.050, 0.056]	[-0.026, 0.015]	[-0.041, 0.035]	[-0.032, 0.017]
Open defecation (group share, R4)		0.760***		0.504***
		(0.028)		(0.046)
Share of households landless		0.050**		0.046*
		(0.019)		(0.025)
Union FEs	Yes	Yes	Yes	Yes
Diff.: Monetary – Public	-0.040	0.000	-0.003	0.023
	(0.039)	(0.016)	(0.030)	(0.022)
p-value	0.299	0.985	0.914	0.285
Diff.: Monetary – Certificate	0.003	-0.001	0.021	0.018
	(0.027)	(0.012)	(0.025)	(0.015)
p-value	0.905	0.940	0.398	0.222
Diff.: Public – Private	-0.014	-0.006	-0.012	-0.007
	(0.027)	(0.011)	(0.023)	(0.013)
p-value	0.606	0.543	0.597	0.598
Number of groups	1,236	1,236	1,235	1,235
Number of villages	107	107	107	107
Omitted category mean	0.132	0.132	0.082	0.082
Omitted category S.D.	(0.195)	(0.195)	(0.188)	(0.188)

Notes: the dependent variable is the share of households primarily practicing OD. Columns (1) and (2) report short-term effects (at the time of assessment); columns (3) and (4) report medium-term effects (12-15 months after assessment). Observations (groups) are weighted by the number of households. The comparison group consists of groups that received the meetings only treatment. Pure control villages are included as a separate category to enhance precision. Standard errors clustered at the village level. Standard errors clustered at the village level in parentheses. Wild cluster bootstrap (9,999 repetitions, Webb weights) 95% confidence intervals, resampling at the village level, in brackets for the coefficients of interest. * $p < 0.10$, ** $p < 0.05$, *** $p < 0.01$.

Table C5: Medium-term Effects: Open Defecation
Based on household's self-report

	(1)	(2)
Monetary Reward	0.079	0.083
	(0.061)	(0.059)
	[-0.069, 0.219]	[-0.055, 0.214]
Reward Certificate	0.061	0.077
	(0.058)	(0.054)
	[-0.067, 0.191]	[-0.048, 0.194]
Private Commitment	0.042	0.039
	(0.057)	(0.053)
	[-0.091, 0.175]	[-0.088, 0.161]
Public Commitment	0.008	0.017
	(0.065)	(0.060)
	[-0.139, 0.154]	[-0.114, 0.151]
Open defecation (group share, R4)		0.414***
		(0.052)
Share of households landless		0.143***
		(0.040)
Union FEs	Yes	Yes
Diff.: Monetary – Public	0.071	0.066
	(0.089)	(0.082)
p-value	0.425	0.424
Diff.: Monetary – Certificate	0.017	0.006
	(0.056)	(0.053)
p-value	0.755	0.912
Diff.: Public – Private	-0.035	-0.022
	(0.055)	(0.051)
p-value	0.525	0.666
Number of groups	1,235	1,235
Number of villages	107	107
Omitted category mean	0.346	0.346
Omitted category S.D.	(0.318)	(0.318)

Notes: the dependent variable is the share of households regularly practicing OD in the medium term (12-15 months after assessment). Observations (groups) are weighted by the number of households. The comparison group consists of groups that received the meetings only treatment. Pure control villages are included as a separate category to enhance precision. Standard errors clustered at the village level. Standard errors clustered at the village level in parentheses. Wild cluster bootstrap (9,999 repetitions, Webb weights) 95% confidence intervals, resampling at the village level, in brackets for the coefficients of interest. * $p < 0.10$, ** $p < 0.05$, *** $p < 0.01$.

D Household Investments and Behaviors

In this appendix, we report full regression results the analysis of household behavior discussed in Section 5.2.1. In each case, we estimate a regression of the form

$$\begin{aligned} y_{hgv} = {} & \beta_1 \text{Incent}_v + \beta_2 \text{Cert}_v + \beta_3 \text{Priv}_v + \beta_4 \text{Publ}_v \\ & + \delta \text{ShareOwnHygienic}_{0gv} + \gamma \text{ShareLandless}_{gv} + \beta_0 \text{PureControl}_v + \varphi_u + \varepsilon_{igv} \end{aligned} \quad \text{(D-1)}$$

where y_{hgv} is the outcome variable of interest (e.g., whether the household owns a latrine with an intact, properly installed slab) for household h in group g in village v, Incent_v and Cert_v are indicators for village v's reward treatment assignment (financial incentive and social incentive, respectively), Priv_v and Publ_v are indicators for village v's commitment treatment assignment (private commitment and public commitment, respectively), $\text{ShareOwnHygienic}_{0gv}$ is the pre-intervention share of households in group g owning a hygienic latrine, $\text{ShareLandless}_{gv}$ is the share of landless households in the group, φ_u is a set of union fixed effects, and ε_{hgv} is an error term which may be correlated at the village level (the level of randomization).

Tables D1 and D2 present results on latrine components, specifically, whether the household owns a latrine with the corresponding component intact and properly installed. These tables correspond to the estimates presented in Figures 5b and 5b of the main text.

Tables D3 and D4 examine outcomes related to latrine maintenance. These estimates correspond to those presented in Figures 6a and 6b of the main text.

Table D5 shows the effects of the treatments on whether the household reported receiving advice, assistance, or pressure from others in the group. These estimates correspond to those presented in Figure 7 of the main text.

Table D6 shows the effects of the treatments on whether the household reported conflicts with neighbors over latrines. This outcome was only collected in the endline survey.

Table D1: Short-term Effects: Latrine Components Functional and Unbroken

	(1) Slab	(2) Seal	(3) Pit Cover and Rings	(4) All components
Monetary Reward	0.033*	0.063***	0.074***	0.109***
	(0.017)	(0.014)	(0.021)	(0.016)
Reward Certificate	0.012	0.009	0.028	0.034**
	(0.016)	(0.011)	(0.019)	(0.013)
Private Commitment	-0.007	0.002	0.004	0.014
	(0.015)	(0.011)	(0.020)	(0.013)
Public Commitment	0.007	0.022	0.043**	0.042***
	(0.018)	(0.013)	(0.020)	(0.015)
Baseline share owning hyg. lat.	0.531***	0.758***	0.467***	0.538***
	(0.034)	(0.021)	(0.038)	(0.031)
Share of households landless	-0.081***	-0.085***	-0.045*	-0.046**
	(0.022)	(0.019)	(0.025)	(0.018)
Union FEs	Yes	Yes	Yes	Yes
Diff.: Monetary – Public	0.027	0.041	0.031	0.067
	(0.029)	(0.019)	(0.028)	(0.020)
p-value	0.355	0.028	0.269	0.001
Diff.: Monetary – Certificate	0.021	0.054	0.046	0.074
	(0.015)	(0.014)	(0.019)	(0.018)
p-value	0.146	0.000	0.017	0.000
Diff.: Public – Private	0.014	0.020	0.039	0.028
	(0.020)	(0.012)	(0.022)	(0.015)
p-value	0.496	0.090	0.075	0.070
Number of households	16,322	15,948	16,323	19,260
Number of groups	1,235	1,235	1,235	1,235
Number of villages	107	107	107	107
Omitted category mean	0.664	0.484	0.522	0.339

Notes: this table shows estimated treatment effects on indicators for whether the household owns a latrine with the component indicated in the column header functional and unbroken in the short term (at the time of assessment). The comparison group consists of groups that received the meetings only treatment. Pure control villages are included as a separate category to enhance precision. Standard errors clustered at the village level. * $p < 0.10$, ** $p < 0.05$, *** $p < 0.01$.

Table D2: Medium-term Effects: Latrine Components Functional and Unbroken

	(1)<br>Slab	(2)<br>Seal	(3)<br>Pit Cover and Rings	(4)<br>All components
Monetary Reward	-0.008	0.025	-0.002	0.000
	(0.017)	(0.023)	(0.023)	(0.014)
Reward Certificate	0.016	0.025	0.024	0.018
	(0.019)	(0.023)	(0.027)	(0.016)
Private Commitment	0.003	0.000	-0.000	0.010
	(0.020)	(0.025)	(0.030)	(0.017)
Public Commitment	0.012	0.015	0.044**	0.024**
	(0.013)	(0.019)	(0.020)	(0.012)
Baseline share owning hyg. lat.	0.407***	0.602***	0.330***	0.195***
	(0.038)	(0.032)	(0.040)	(0.020)
Share of households landless	-0.126***	-0.126***	-0.116***	-0.043***
	(0.026)	(0.030)	(0.028)	(0.016)
Union FEs	Yes	Yes	Yes	Yes
Diff.: Monetary – Public	-0.020	0.010	-0.046	-0.024
	(0.022)	(0.034)	(0.029)	(0.020)
p-value	0.363	0.777	0.116	0.235
Diff.: Monetary – Certificate	-0.024	-0.000	-0.025	-0.018
	(0.015)	(0.021)	(0.021)	(0.013)
p-value	0.117	0.996	0.231	0.157
Diff.: Public – Private	0.009	0.015	0.044	0.014
	(0.019)	(0.023)	(0.026)	(0.016)
p-value	0.634	0.515	0.095	0.378
Number of households	7,972	7,884	7,954	19,260
Number of groups	1,234	1,234	1,234	1,235
Number of villages	107	107	107	107
Omitted category mean	0.734	0.604	0.642	0.199

Notes: this table shows estimated treatment effects on indicators for whether the household owns a latrine with the component indicated in the column header functional and unbroken in the medium term (12-15 months after assessment). The comparison group consists of groups that received the meetings only treatment. Pure control villages are included as a separate category to enhance precision. Standard errors clustered at the village level. * $p < 0.10$, ** $p < 0.05$, *** $p < 0.01$.

Table D3: Short-term Effects: Latrine Condition

	(1) No Bad Smell	(2) No Leaks	(3) No Flies	(4) Water	(5) Soap
Monetary Reward	0.088***	0.054***	0.180***	0.096***	0.079***
	(0.023)	(0.018)	(0.024)	(0.019)	(0.019)
Reward Certificate	-0.021	0.010	0.054***	-0.000	-0.000
	(0.026)	(0.018)	(0.019)	(0.017)	(0.022)
Private Commitment	-0.028	0.003	0.022	0.017	0.025
	(0.026)	(0.018)	(0.019)	(0.018)	(0.024)
Public Commitment	0.041*	0.036*	0.033	-0.030	0.012
	(0.023)	(0.019)	(0.022)	(0.018)	(0.017)
Baseline share owning hyg. lat.	0.480***	0.448***	0.589***	0.533***	0.475***
	(0.029)	(0.030)	(0.030)	(0.028)	(0.034)
Share of households landless	-0.036	-0.030	-0.032	-0.108***	-0.125***
	(0.022)	(0.026)	(0.023)	(0.021)	(0.021)
Union FEs	Yes	Yes	Yes	Yes	Yes
Diff.: Monetary – Public	0.046	0.018	0.147	0.126	0.067
	(0.034)	(0.026)	(0.034)	(0.025)	(0.022)
p-value	0.171	0.495	0.000	0.000	0.002
Diff.: Monetary – Certificate	0.109	0.044	0.126	0.096	0.079
	(0.026)	(0.015)	(0.026)	(0.018)	(0.018)
p-value	0.000	0.005	0.000	0.000	0.000
Diff.: Public – Private	0.069	0.033	0.012	-0.047	-0.012
	(0.030)	(0.018)	(0.023)	(0.017)	(0.020)
p-value	0.024	0.072	0.606	0.007	0.529
Number of households	16,322	16,320	16,307	16,013	16,009
Number of groups	1,235	1,235	1,235	1,235	1,235
Number of villages	107	107	107	107	107
Omitted category mean	0.323	0.614	0.340	0.398	0.299

Notes: this table shows estimated treatment effects on indicators for whether the household owns a latrine in the condition noted in the column header, as observed at the time of assessment (short term). The comparison group consists of groups that received the meetings only treatment. Pure control villages are included as a separate category to enhance precision. Standard errors clustered at the village level. * $p < 0.10$, ** $p < 0.05$, *** $p < 0.01$.

Table D4: Medium-term Effects: Latrine Condition

	(1)<br>No Bad Smell	(2)<br>No Leaks	(3)<br>No Flies	(4)<br>Water	(5)<br>Soap
Monetary Reward	-0.046	0.014	-0.011	0.013	0.020
	(0.040)	(0.019)	(0.039)	(0.028)	(0.030)
Reward Certificate	-0.065*	0.013	-0.093*	0.011	0.045*
	(0.033)	(0.021)	(0.054)	(0.030)	(0.025)
Private Commitment	-0.001	0.005	-0.068	-0.002	0.030
	(0.033)	(0.023)	(0.057)	(0.032)	(0.028)
Public Commitment	0.089**	0.033*	0.016	0.007	0.019
	(0.039)	(0.017)	(0.041)	(0.025)	(0.026)
Baseline share owning hyg. lat.	0.189***	0.311***	0.236***	0.442***	0.400***
	(0.045)	(0.035)	(0.048)	(0.048)	(0.043)
Share of households landless	-0.088***	-0.116***	-0.100***	-0.144***	-0.151***
	(0.028)	(0.028)	(0.035)	(0.033)	(0.036)
Union FEs	Yes	Yes	Yes	Yes	Yes
Diff.: Monetary – Public	-0.135	-0.019	-0.028	0.006	0.001
	(0.055)	(0.026)	(0.059)	(0.032)	(0.038)
p-value	0.016	0.480	0.642	0.844	0.971
Diff.: Monetary – Certificate	0.019	0.001	0.082	0.002	-0.025
	(0.041)	(0.019)	(0.038)	(0.022)	(0.029)
p-value	0.640	0.954	0.033	0.920	0.385
Diff.: Public – Private	0.090	0.028	0.085	0.009	-0.012
	(0.039)	(0.022)	(0.053)	(0.027)	(0.026)
p-value	0.024	0.201	0.116	0.746	0.658
Number of households	7,974	7,972	7,970	7,618	7,972
Number of groups	1,234	1,234	1,234	1,234	1,234
Number of villages	107	107	107	107	107
Omitted category mean	0.314	0.700	0.417	0.534	0.387

Notes: this table shows estimated treatment effects on indicators for whether the household owns a latrine in the condition noted in the column header, as observed 12-15 months after assessment (medium term). The comparison group consists of groups that received the meetings only treatment. Pure control villages are included as a separate category to enhance precision. Standard errors clustered at the village level. * $p < 0.10$, ** $p < 0.05$, *** $p < 0.01$.

Table D5: Short-term Effects: Assistance from others in group

	(1) Any	(2) Advice or info.	(3) Privately	(4) Publicly	(5) Pressure	(6) Materials, cash, labor
Monetary Reward	0.149***	0.143***	0.044*	0.138***	0.118***	0.005***
	(0.032)	(0.033)	(0.026)	(0.030)	(0.031)	(0.002)
Reward Certificate	0.080**	0.078**	0.038	0.081**	0.034*	-0.000
	(0.037)	(0.037)	(0.027)	(0.040)	(0.019)	(0.001)
Private Commitment	0.085**	0.084**	0.017	0.079*	0.019	-0.000
	(0.040)	(0.040)	(0.028)	(0.043)	(0.020)	(0.001)
Public Commitment	0.073**	0.071**	0.029	0.056*	0.048*	0.003*
	(0.035)	(0.035)	(0.034)	(0.030)	(0.025)	(0.002)
Baseline share owning hyg. lat.	0.191***	0.192***	0.079**	0.174***	0.148***	-0.003
	(0.040)	(0.041)	(0.033)	(0.041)	(0.027)	(0.002)
Share of households landless	0.013	0.014	0.009	0.030	0.021	-0.002
	(0.030)	(0.030)	(0.027)	(0.024)	(0.023)	(0.001)
Union FEs	Yes	Yes	Yes	Yes	Yes	Yes
Diff.: Monetary – Public	0.076	0.072	0.015	0.082	0.070	0.002
	(0.046)	(0.046)	(0.040)	(0.042)	(0.040)	(0.003)
p-value	0.097	0.123	0.702	0.053	0.082	0.368
Diff.: Monetary – Certificate	0.069	0.065	0.007	0.056	0.084	0.006
	(0.036)	(0.037)	(0.031)	(0.034)	(0.030)	(0.002)
p-value	0.061	0.082	0.829	0.103	0.006	0.001
Diff.: Public – Private	-0.012	-0.012	0.012	-0.023	0.029	0.003
	(0.039)	(0.040)	(0.027)	(0.043)	(0.027)	(0.002)
p-value	0.761	0.759	0.651	0.595	0.280	0.063
Number of households	15,950	15,889	15,931	15,931	15,936	15,889
Number of groups	1,235	1,235	1,235	1,235	1,235	1,235
Number of villages	107	107	107	107	107	107
Omitted category mean	0.258	0.259	0.187	0.183	0.153	0.001

Notes: this table shows estimated treatment effects on indicators for different types of assistance (noted in the column header) the household reports receiving from others in the group. Outcomes are in the short term (at the time of assessment). The comparison group consists of groups that received the meetings only treatment. Pure control villages are included as a separate category to enhance precision. Standard errors clustered at the village level in parentheses. * $p < 0.10$, ** $p < 0.05$, *** $p < 0.01$.

Table D6: Medium-term Effects: Conflicts with Neighbors over Latrines

	(1)	(2)	(3)	(4)
Monetary Reward	-0.001	-0.003	-0.002	-0.002
	(0.003)	(0.004)	(0.004)	(0.004)
Reward Certificate	-0.005*	-0.005*	-0.005	-0.005
	(0.003)	(0.003)	(0.003)	(0.003)
Private Commitment	-0.004	-0.004	-0.004	-0.004
	(0.003)	(0.003)	(0.003)	(0.003)
Public Commitment	-0.006**	-0.009**	-0.008**	-0.008**
	(0.003)	(0.003)	(0.003)	(0.003)
Baseline share owning hyg. lat.			-0.015**	-0.012*
			(0.007)	(0.006)
Share of households landless				0.007
				(0.006)
Union FEs	No	Yes	Yes	Yes
Number of households	7,979	7,979	7,974	7,974
Number of groups	1,235	1,235	1,234	1,234
Number of villages	107	107	107	107
Omitted category mean	0.012	0.012	0.012	0.012

Notes: the dependent variable is an indicator for whether the household reports conflict with neighbors over latrines in the medium term (12-15 months after assessment). The comparison group consists of groups that received the meetings only treatment. Pure control villages are included as a separate category to enhance precision. Standard errors clustered at the village level. * $p < 0.10$, ** $p < 0.05$, *** $p < 0.01$.

E Interaction with Household Characteristics

In this appendix, we report full regression results for our main outcome of interest (hygienic latrine ownership) interacting treatment indicators with household characteristics, as discussed in Section 5.2 of the main text. The regression equation is given by Equation 2 in the main text, which we reproduce here for convenience:

$$\begin{aligned} y_{hgv} &= \alpha_0 + \alpha_1 D_{hgv} \\ &+ \sum_{p=0}^{4} \beta_p \cdot 1\{1\text{Treat}_v = p\} + \sum_{p=0}^{4} \theta_p \cdot 1\{\text{Treat}_v = p\} \times D_{hgv} \\ &+ \delta y_{0gv} + \gamma \text{ShareLandless}_{gv} + \varphi_u + \varepsilon_{hgv} \end{aligned} \tag{2}$$

The estimates in Table E1 do not include any household characteristic D_{hgv}, and are included to show that regressions using household-level data are similar to those using group-level data as reported in the main text.

In Table E2, D_{hgv} is an indicator for whether the household is landless, a proxy for poverty. The estimates plotted in Figure 8 of the main text correspond to columns (2) (short-term) and (4) (medium-term) of this table.

In Table E3, D_{hgv} is a categorical variable for the household's latrine ownership status at baseline. The omitted category consists of households not owning any latrine at baseline. The estimates plotted in Figure 9 of the main text correspond to columns (2) (short-term) and (4) (medium-term) of this table.

Table E1: Program Effects: Hygienic Latrine Ownership
Household-level Data

	Short term		Medium term	
	(1)	(2)	(3)	(4)
Monetary Reward	0.125***	0.078***	0.049	0.017
	(0.033)	(0.014)	(0.031)	(0.021)
Reward Certificate	0.044	0.012	0.044	0.022
	(0.035)	(0.012)	(0.037)	(0.024)
Private Commitment	0.013	0.013	0.014	0.015
	(0.036)	(0.011)	(0.040)	(0.026)
Public Commitment	0.063*	0.047***	0.068**	0.050***
	(0.035)	(0.014)	(0.029)	(0.018)
Baseline share owning hyg. lat.		0.692***		0.510***
		(0.020)		(0.035)
Share of households landless		-0.064***		-0.094***
		(0.017)		(0.030)
Union FEs	Yes	Yes	Yes	Yes
Number of households	16,352	16,347	7,979	7,974
Number of groups	1,236	1,235	1,235	1,234
Number of villages	107	107	107	107
Omitted category mean	0.429	0.429	0.551	0.551

Notes: the dependent variable is an indicator for whether the household owns a hygienic latrine. Columns (1) and (2) report short-term effects (at the time of assessment); columns (3) and (4) report medium-term effects (12-15 months after assessment). The comparison group consists of groups that received the meetings only treatment. Pure control villages are included as a separate category to enhance precision. Standard errors clustered at the village level. * $p < 0.10$, ** $p < 0.05$, *** $p < 0.01$.

Table E2: Program Effects: Hygienic Latrine Ownership
Household-Level Data – By Landlessness

	Short term		Medium term	
	(1)	(2)	(3)	(4)
Monetary Reward	0.131***	0.078***	0.042	0.005
	(0.030)	(0.016)	(0.031)	(0.025)
Monetary Reward X Household landless	-0.011	-0.011	0.026	0.032
	(0.040)	(0.025)	(0.039)	(0.034)
Reward Certificate	0.048	0.016	0.036	0.014
	(0.038)	(0.015)	(0.037)	(0.028)
Reward Certificate X Household landless	-0.014	-0.017	0.027	0.023
	(0.031)	(0.021)	(0.036)	(0.033)
Private Commitment	0.008	0.009	0.028	0.033
	(0.039)	(0.015)	(0.040)	(0.030)
Private Commitment X Household landless	-0.008	-0.004	-0.042	-0.046
	(0.035)	(0.024)	(0.039)	(0.034)
Public Commitment	0.057*	0.042***	0.071**	0.059***
	(0.032)	(0.016)	(0.028)	(0.022)
Public Commitment X Household landless	0.002	0.001	-0.028	-0.029
	(0.035)	(0.022)	(0.043)	(0.037)
Household landless	-0.203***	-0.163***	-0.158***	-0.121***
	(0.026)	(0.020)	(0.030)	(0.030)
Baseline share owning hyg. lat.		0.715***		0.509***
		(0.021)		(0.036)
Group share landless		0.102***		0.031
		(0.018)		(0.032)
Union FEs	Yes	Yes	Yes	Yes
Number of households	15,204	15,199	7,974	7,969
Number of groups	1,236	1,235	1,235	1,234
Number of villages	107	107	107	107
Omitted category mean	0.452	0.452	0.552	0.552

Notes: the dependent variable is an indicator for whether the household owns a hygienic latrine. Columns (1) and (2) report short-term effects (at the time of assessment); columns (3) and (4) report medium-term effects (12-15 months after assessment). The comparison group consists of groups that received the meetings only treatment. Pure control villages are included as a separate category to enhance precision. Standard errors clustered at the village level. * $p < 0.10$, ** $p < 0.05$, *** $p < 0.01$.

Table E3: Program Effects: Hygienic Latrine Ownership
Household-Level Data – By Baseline Ownership Category

	Short term		Medium term	
	(1)	(2)	(3)	(4)
Monetary Reward	0.084***	0.078***	0.010	0.002
	(0.025)	(0.022)	(0.037)	(0.034)
Monetary Reward X Owns non-hygienic latrine	0.007	0.010	0.056	0.058
	(0.023)	(0.023)	(0.041)	(0.041)
Monetary Reward X Owns hygienic latrine	-0.017	-0.015	0.014	0.017
	(0.035)	(0.033)	(0.043)	(0.041)
Reward Certificate	0.004	-0.001	0.019	0.013
	(0.021)	(0.017)	(0.038)	(0.035)
Reward Certificate X Owns non-hygienic latrine	0.000	0.003	0.008	0.011
	(0.021)	(0.021)	(0.043)	(0.041)
Reward Certificate X Owns hygienic latrine	0.020	0.021	0.018	0.018
	(0.027)	(0.025)	(0.042)	(0.040)
Private Commitment	0.001	0.001	0.014	0.012
	(0.021)	(0.017)	(0.042)	(0.038)
Private Commitment X Owns non-hygienic latrine	0.027	0.026	-0.014	-0.009
	(0.022)	(0.022)	(0.045)	(0.043)
Private Commitment X Owns hygienic latrine	0.013	0.011	0.021	0.020
	(0.028)	(0.026)	(0.046)	(0.043)
Public Commitment	0.054**	0.050**	0.035	0.026
	(0.025)	(0.022)	(0.036)	(0.032)
Public Commitment X Owns non-hygienic latrine	0.021	0.023	0.070	0.076*
	(0.022)	(0.022)	(0.044)	(0.043)
Public Commitment X Owns hygienic latrine	-0.025	-0.023	-0.006	-0.000
	(0.032)	(0.029)	(0.043)	(0.041)
Owns non-hygienic latrine	-0.012	-0.015	-0.028	-0.039
	(0.019)	(0.020)	(0.039)	(0.038)
Owns hygienic latrine	0.643***	0.618***	0.413***	0.377***
	(0.029)	(0.027)	(0.040)	(0.038)
Baseline share owning hyg. lat.		0.104***		0.133***
		(0.021)		(0.033)
Share of households landless		-0.067***		-0.097***
		(0.017)		(0.029)
Union FEs	Yes	Yes	Yes	Yes
Number of households	15,927	15,927	7,827	7,827
Number of groups	1,235	1,235	1,234	1,234
Number of villages	107	107	107	107
Omitted category mean	0.432	0.432	0.553	0.553

Notes: the dependent variable is an indicator for whether the household owns a hygienic latrine. Columns (1) and (2) report short-term effects (at the time of assessment); columns (3) and (4) report medium-term effects (12-15 months after assessment). The comparison group consists of groups that received the meetings only treatment. Pure control villages are included as a separate category to enhance precision. Standard errors clustered at the village level. * $p < 0.10$, ** $p < 0.05$, *** $p < 0.01$.

F Interaction with Group Characteristics

In this appendix, we report full regression results for our main outcome of interest (hygienic latrine ownership) interacting treatment indicators with group characteristics, as discussed in Section 5.2.3.

First, in Table F1, we report estimates of Equation 3, interacting the reward treatments (monetary and certificate) with the group's distance to the nearest reward threshold above its baseline status. Columns (1) and (2) report short-term results and Columns (3) and (4) report medium-term results. Columns (2) and (4) correspond to the specification plotted in Figure 10 in the main text.

Second, in Table F2, we report estimates of Equation 5, interacting the treatments with the baseline share of households in the group owning a hygienic latrine. Columns (1) and (2) report short-term results and Columns (3) and (4) report medium-term results. Columns (2) and (4) correspond to the specification plotted in Figure 13 in the main text.

Finally, we report results from our ex-post exploratory analyses. Similar to equation 5 in the main text, we estimate

$$\begin{aligned} y_{gv} &= \alpha_0 + \alpha_1 D_{gv} \\ &+ \sum_{p=0}^{4} \beta_p \cdot 1\{1\text{Treat}_v = p\} + \sum_{p=0}^{4} \theta_p \cdot 1\{\text{Treat}_v = p\} \times D_{gv} \\ &+ \delta y_{0gv} + \gamma \text{ShareLandless}_{gv} + \varphi_u + \varepsilon_{gv} \end{aligned} \quad \text{(F-1)}$$

where D_{gv} is a characteristic of group g and all other variables are as defined in equation (5). As in equation (5), α_1 represents the level effect of characteristic D, the coefficient β_p represents the level effect of treatment p,[1] and the coefficient θ_p is the interaction between treatment p and characteristic D.

We considered the following characteristics, which were relevant as proxies for resources available to the group, baseline sanitation status beyond hygienic latrine ownership, strength of potential epidemiological externalities, or group social cohesion:

- Share of landless households in the group (Table F3)
- Baseline ownership of any latrine (Table F4)
- Baseline ownership of non-hygienic latrines (Table F5)
- Whether the group contained an individual considered by others in the village to be a village leader (Table F6)
- Group size (number of households) (Table F7)

[1]For discrete D, this is the effect of treatment p on groups in the reference category, i.e., with $D = 0$. For continuous D, as in in equation (5), we de-mean the interaction variable so β_p represents the effect of treatment p on groups with the mean level of D (Wainer 2000).

- Group density (the average number of households within 50m of each household in the group) (Table F8)
- Two social network statistics calculated using baseline data on household relationships within the village
  - Maximum eigenvalue of adjacency matrix, interpretable as the speed at which information will spread within the group (Table F9)
  - The second eigenvalue of the stochastized adjacency matrix, interpretable as how segregated a network is, i.e., negatively related to the extent to which information will spread within the group (Table F10)

Overall, we do not find strong evidence of an association between these variables and the size of our estimated treatment effects. There is a weak negative association between the share of landless household in the group and the effectiveness of the monetary reward treatment, although only in the short term (Table F3). Contrary to our expectation, the presence of a village leader in a group is negatively associated with the effectiveness of the monetary reward and reward certificate treatments, although again only in the short term (Table F6). The effectiveness of the monetary reward treatment is negatively associated with the network segregation measure, once again in the short term only (Table F10). Given the large number of hypotheses tested and the relatively low power to detect interactions, we view these results as suggestive only.

Table F1: Program Effects: Hygienic Latrine Ownership
Interacted with Distance to Reward Threshold

	Short term		Medium term	
	(1)	(2)	(3)	(4)
Monetary Reward	0.110***	0.086***	0.029	0.010
	(0.031)	(0.019)	(0.028)	(0.022)
Monetary Reward × Distance to nearest threshold above	-0.198	0.007	-0.117	0.047
	(0.206)	(0.137)	(0.224)	(0.172)
Reward Certificate	0.016	0.010	0.009	0.004
	(0.027)	(0.013)	(0.030)	(0.023)
Reward Certificate × Distance to nearest threshold above	0.127	0.004	0.013	-0.090
	(0.154)	(0.100)	(0.195)	(0.164)
Private Commitment	-0.004	0.009	-0.005	0.005
	(0.026)	(0.013)	(0.032)	(0.027)
Public Commitment	0.061**	0.048***	0.067**	0.056***
	(0.030)	(0.018)	(0.027)	(0.020)
Distance to nearest threshold above	-0.624***	0.024	-0.371***	0.173
	(0.106)	(0.080)	(0.135)	(0.121)
Baseline share owning hyg. lat.		0.742***		0.609***
		(0.039)		(0.053)
Share of households landless		-0.089***		-0.098***
		(0.018)		(0.035)
Union FEs	Yes	Yes	Yes	Yes
Number of groups	1,028	1,028	1,027	1,027
Number of villages	106	106	106	106
Omitted category mean	0.421	0.421	0.535	0.535
Omitted category S.D.	(0.175)	(0.175)	(0.258)	(0.258)

Notes: the dependent variable is the share of households in the group owning a hygienic latrine Columns (1) and (2) report short-term effects (at the time of assessment); columns (3) and (4) report medium-term effects (12-15 months after assessment).. Interactions with distance to threshold are estimated for the reward treatments (monetary reward, recognition reward). The thresholds were not relevant for the commitment treatments, so no interaction is estimated. Distance to threshold is de-meaned, so the level terms represent the effect of treatment at the mean value of distance to threshold (15.8 percentage points). Observations (groups) are weighted by the number of households. The comparison group consists of groups that received the meetings only treatment. Pure control villages are included as a separate category to enhance precision. Groups above the higher threshold for their union are dropped. Standard errors clustered at the village level. Standard errors clustered at the village level in parentheses. * $p < 0.10$, ** $p < 0.05$, *** $p < 0.01$.

Table F2: Program Effects: Hygienic Latrine Ownership
Interacted with Group Baseline Hygienic Latrine Ownership Share

	Short term		Medium term	
	(1)	(2)	(3)	(4)
Monetary Reward	0.072***	0.076***	0.008	0.011
	(0.016)	(0.016)	(0.021)	(0.020)
Monetary Reward X Baseline share owning hyg. latr.	0.067	0.058	0.007	-0.002
	(0.070)	(0.065)	(0.079)	(0.075)
Reward Certificate	0.008	0.009	0.013	0.014
	(0.012)	(0.012)	(0.022)	(0.022)
Reward Certificate X Baseline share owning hyg. latr.	0.022	0.023	0.107	0.108
	(0.053)	(0.050)	(0.076)	(0.074)
Private Commitment	0.009	0.008	0.009	0.008
	(0.012)	(0.012)	(0.025)	(0.024)
Private Commitment X Baseline share owning hyg. latr.	0.056	0.056	0.080	0.080
	(0.054)	(0.051)	(0.079)	(0.076)
Public Commitment	0.043***	0.042***	0.056***	0.055***
	(0.016)	(0.015)	(0.018)	(0.018)
Public Commitment X Baseline share owning hyg. latr.	0.043	0.049	-0.033	-0.025
	(0.065)	(0.062)	(0.082)	(0.079)
Baseline share owning hyg. latr.	0.696***	0.661***	0.516***	0.477***
	(0.057)	(0.054)	(0.076)	(0.074)
Share of households landless		-0.084***		-0.092***
		(0.018)		(0.031)
Union FEs	Yes	Yes	Yes	Yes
Number of groups	1,235	1,235	1,234	1,234
Number of villages	107	107	107	107
Omitted category mean	0.451	0.451	0.544	0.544
Omitted category S.D.	(0.189)	(0.189)	(0.255)	(0.255)

Notes: the dependent variable is the share of households in the group with a hygienic latrine. Columns (1) and (2) report short-term effects (at the time of assessment); columns (3) and (4) report medium-term effects (12-15 months after assessment). Observations (groups) are weighted by the number of households. The comparison group consists of groups that received the meetings only treatment. Pure control villages are included as a separate category to enhance precision. Standard errors clustered at the village level. Standard errors clustered at the village level in parentheses. * $p < 0.10$, ** $p < 0.05$, *** $p < 0.01$.

Table F3: Program Effects: Hygienic Latrine Ownership
Interacted with Group Share Landless

	Short term		Medium term	
	(1)	(2)	(3)	(4)
Monetary Reward	0.129***	0.077***	0.049*	0.012
	(0.029)	(0.014)	(0.027)	(0.020)
Monetary Reward X Group share landless	-0.059	-0.086**	0.095	0.074
	(0.091)	(0.043)	(0.084)	(0.059)
Reward Certificate	0.042	0.009	0.041	0.018
	(0.033)	(0.012)	(0.031)	(0.022)
Reward Certificate X Group share landless	0.007	-0.035	0.061	0.030
	(0.093)	(0.044)	(0.089)	(0.066)
Private Commitment	0.005	0.008	0.011	0.013
	(0.033)	(0.012)	(0.034)	(0.024)
Private Commitment X Group share landless	-0.056	-0.051	-0.070	-0.067
	(0.095)	(0.042)	(0.086)	(0.057)
Public Commitment	0.057*	0.044***	0.067**	0.058***
	(0.033)	(0.015)	(0.027)	(0.017)
Public Commitment X Group share landless	-0.046	-0.043	-0.003	-0.003
	(0.100)	(0.047)	(0.096)	(0.073)
Group share landless	-0.284***	-0.009	-0.322***	-0.124**
	(0.083)	(0.044)	(0.076)	(0.055)
Baseline share owning hyg. lat.		0.709***		0.511***
		(0.022)		(0.036)
Union FEs	Yes	Yes	Yes	Yes
Number of groups	1,236	1,235	1,235	1,234
Number of villages	107	107	107	107
Omitted category mean	0.451	0.451	0.544	0.544
Omitted category S.D.	(0.189)	(0.189)	(0.255)	(0.255)

Notes: the dependent variable is the share of households in the group with a hygienic latrine. Columns (1) and (2) report short-term effects (at the time of assessment); columns (3) and (4) report medium-term effects (12-15 months after assessment). Observations (groups) are weighted by the number of households. The comparison group consists of groups that received the meetings only treatment. Pure control villages are included as a separate category to enhance precision. Standard errors clustered at the village level. Standard errors clustered at the village level in parentheses. * $p < 0.10$, ** $p < 0.05$, *** $p < 0.01$.

Table F4: Program Effects: Hygienic Latrine Ownership
Interacted with Baseline Latrine Ownership Share

	Short term		Medium term	
	(1)	(2)	(3)	(4)
Monetary Reward	0.092***	0.098***	0.021	0.026
	(0.026)	(0.024)	(0.026)	(0.025)
Monetary Reward X Baseline share owning any latr.	0.016	-0.001	0.094	0.081
	(0.079)	(0.073)	(0.100)	(0.095)
Reward Certificate	0.020	0.022	0.024	0.026
	(0.024)	(0.024)	(0.027)	(0.026)
Reward Certificate X Baseline share owning any latr.	0.006	0.020	0.142	0.154*
	(0.056)	(0.052)	(0.095)	(0.091)
Private Commitment	0.010	0.007	0.010	0.007
	(0.024)	(0.024)	(0.029)	(0.028)
Private Commitment X Baseline share owning any latr.	0.054	0.057	0.022	0.025
	(0.063)	(0.059)	(0.104)	(0.101)
Public Commitment	0.047*	0.044*	0.054**	0.052**
	(0.028)	(0.027)	(0.022)	(0.022)
Public Commitment X Baseline share owning any latr.	0.050	0.076	0.036	0.057
	(0.070)	(0.067)	(0.087)	(0.082)
Baseline share owning any latr.	0.547***	0.465***	0.399***	0.333***
	(0.052)	(0.050)	(0.079)	(0.077)
Share of households landless		-0.205***		-0.165***
		(0.029)		(0.035)
Union FEs	Yes	Yes	Yes	Yes
Number of groups	1,235	1,235	1,234	1,234
Number of villages	107	107	107	107
Omitted category mean	0.451	0.451	0.544	0.544
Omitted category S.D.	(0.189)	(0.189)	(0.255)	(0.255)

Notes: the dependent variable is the share of households in the group with a hygienic latrine. Columns (1) and (2) report short-term effects (at the time of assessment); columns (3) and (4) report medium-term effects (12-15 months after assessment). Observations (groups) are weighted by the number of households. The comparison group consists of groups that received the meetings only treatment. Pure control villages are included as a separate category to enhance precision. Standard errors clustered at the village level. Standard errors clustered at the village level in parentheses. * $p < 0.10$, ** $p < 0.05$, *** $p < 0.01$.

Table F5: Program Effects: Hygienic Latrine Ownership
Interacted with Baseline Non-Hygienic Latrine Ownership Share

	Short term		Medium term	
	(1)	(2)	(3)	(4)
Monetary Reward	0.118***	0.078***	0.043	0.012
	(0.030)	(0.015)	(0.028)	(0.020)
Monetary Reward X Baseline share owning non-hyg. latr.	-0.222*	-0.128	0.032	0.103
	(0.126)	(0.080)	(0.141)	(0.114)
Reward Certificate	0.037	0.010	0.037	0.016
	(0.032)	(0.012)	(0.032)	(0.022)
Reward Certificate X Baseline share owning non-hyg. latr.	-0.218	-0.106	-0.147	-0.057
	(0.136)	(0.068)	(0.139)	(0.106)
Private Commitment	0.007	0.009	0.009	0.011
	(0.033)	(0.012)	(0.035)	(0.024)
Private Commitment X Baseline share owning non-hyg. latr.	0.007	0.010	-0.113	-0.111
	(0.127)	(0.072)	(0.130)	(0.100)
Public Commitment	0.065**	0.045***	0.073***	0.057***
	(0.031)	(0.015)	(0.026)	(0.018)
Public Commitment X Baseline share owning non-hyg. latr.	-0.085	-0.009	0.045	0.101
	(0.092)	(0.065)	(0.130)	(0.106)
Baseline share owning non-hyg. latr.	-0.265***	0.095	-0.188*	0.089
	(0.084)	(0.068)	(0.104)	(0.098)
Share of households landless		-0.082***		-0.086***
		(0.018)		(0.032)
Baseline share owning hyg. lat.		0.711***		0.543***
		(0.027)		(0.042)
Union FEs	Yes	Yes	Yes	Yes
Number of groups	1,235	1,235	1,234	1,234
Number of villages	107	107	107	107
Omitted category mean	0.451	0.451	0.544	0.544
Omitted category S.D.	(0.189)	(0.189)	(0.255)	(0.255)

Notes: the dependent variable is the share of households in the group with a hygienic latrine. Columns (1) and (2) report short-term effects (at the time of assessment); columns (3) and (4) report medium-term effects (12-15 months after assessment). Observations (groups) are weighted by the number of households. The comparison group consists of groups that received the meetings only treatment. Pure control villages are included as a separate category to enhance precision. Standard errors clustered at the village level. Standard errors clustered at the village level in parentheses. * $p < 0.10$, ** $p < 0.05$, *** $p < 0.01$.

Table F6: Program Effects: Hygienic Latrine Ownership
Interacted with Village Leader Present

	Short term		Medium term	
	(1)	(2)	(3)	(4)
Monetary Reward	0.138***	0.091***	0.058*	0.024
	(0.038)	(0.015)	(0.033)	(0.021)
Monetary Reward X Village Leader Present in Group	-0.074	-0.068*	-0.068	-0.064
	(0.054)	(0.035)	(0.050)	(0.043)
Reward Certificate	0.058	0.021*	0.056	0.029
	(0.040)	(0.012)	(0.038)	(0.023)
Reward Certificate X Village Leader Present in Group	-0.088*	-0.059**	-0.087	-0.065
	(0.046)	(0.027)	(0.052)	(0.045)
Private Commitment	0.019	0.011	0.015	0.009
	(0.040)	(0.012)	(0.040)	(0.025)
Private Commitment X Village Leader Present in Group	-0.058	-0.016	-0.008	0.023
	(0.041)	(0.029)	(0.050)	(0.044)
Public Commitment	0.064	0.039**	0.071**	0.051***
	(0.040)	(0.015)	(0.031)	(0.017)
Public Commitment X Village Leader Present in Group	-0.019	0.016	-0.011	0.017
	(0.048)	(0.030)	(0.051)	(0.045)
Village Leader Present in Group	0.110***	0.035	0.077	0.020
	(0.039)	(0.027)	(0.049)	(0.046)
Share of households landless		-0.086***		-0.091***
		(0.017)		(0.032)
Baseline share owning hyg. lat.		0.706***		0.513***
		(0.022)		(0.036)
Union FEs	Yes	Yes	Yes	Yes
Number of groups	1,236	1,235	1,235	1,234
Number of villages	107	107	107	107
Omitted category mean	0.451	0.451	0.544	0.544
Omitted category S.D.	(0.189)	(0.189)	(0.255)	(0.255)

Notes: the dependent variable is the share of households in the group with a hygienic latrine. Columns (1) and (2) report short-term effects (at the time of assessment); columns (3) and (4) report medium-term effects (12-15 months after assessment). Observations (groups) are weighted by the number of households. The comparison group consists of groups that received the meetings only treatment. Pure control villages are included as a separate category to enhance precision. Standard errors clustered at the village level. Standard errors clustered at the village level in parentheses. * $p < 0.10$, ** $p < 0.05$, *** $p < 0.01$.

Table F7: Program Effects: Hygienic Latrine Ownership
Interacted with Group Size (Number of Households)

	Short term		Medium term	
	(1)	(2)	(3)	(4)
Monetary Reward	0.128***	0.078***	0.051*	0.014
	(0.035)	(0.015)	(0.030)	(0.020)
Monetary Reward X Num. HH in group	0.000	-0.001	-0.002	-0.002
	(0.008)	(0.004)	(0.009)	(0.008)
Reward Certificate	0.048	0.012	0.045	0.018
	(0.039)	(0.012)	(0.039)	(0.023)
Reward Certificate X Num. HH in group	-0.009	-0.002	-0.007	-0.002
	(0.008)	(0.003)	(0.009)	(0.007)
Private Commitment	0.010	0.009	0.017	0.016
	(0.038)	(0.012)	(0.039)	(0.025)
Private Commitment X Num. HH in group	-0.010	-0.004	-0.011	-0.007
	(0.007)	(0.003)	(0.007)	(0.005)
Public Commitment	0.065*	0.043***	0.076***	0.059***
	(0.037)	(0.016)	(0.029)	(0.017)
Public Commitment X Num. HH in group	-0.006	0.001	-0.011*	-0.006
	(0.007)	(0.004)	(0.007)	(0.007)
Num. HH in group	0.009	0.002	0.014**	0.008
	(0.006)	(0.003)	(0.007)	(0.006)
Baseline share owning hyg. lat.		0.710***		0.516***
		(0.022)		(0.036)
Share of households landless		-0.082***		-0.086***
		(0.017)		(0.032)
Union FEs	Yes	Yes	Yes	Yes
Number of groups	1,236	1,235	1,235	1,234
Number of villages	107	107	107	107
Omitted category mean	0.451	0.451	0.544	0.544
Omitted category S.D.	(0.189)	(0.189)	(0.255)	(0.255)

Notes: the dependent variable is the share of households in the group with a hygienic latrine. Columns (1) and (2) report short-term effects (at the time of assessment); columns (3) and (4) report medium-term effects (12-15 months after assessment). Observations (groups) are weighted by the number of households. The comparison group consists of groups that received the meetings only treatment. Pure control villages are included as a separate category to enhance precision. Standard errors clustered at the village level. Standard errors clustered at the village level in parentheses. * $p < 0.10$, ** $p < 0.05$, *** $p < 0.01$.

Table F8: Program Effects: Hygienic Latrine Ownership
Interacted with Group Density

	Short term		Medium term	
	(1)	(2)	(3)	(4)
Monetary Reward	0.127***	0.078***	0.047	0.011
	(0.032)	(0.015)	(0.030)	(0.020)
Monetary Reward X Group density	0.003	0.003	0.003	0.003
	(0.004)	(0.002)	(0.004)	(0.003)
Reward Certificate	0.042	0.010	0.042	0.018
	(0.035)	(0.012)	(0.035)	(0.023)
Reward Certificate X Group density	-0.004	-0.001	-0.001	0.001
	(0.003)	(0.002)	(0.004)	(0.003)
Private Commitment	0.008	0.008	0.012	0.011
	(0.038)	(0.012)	(0.039)	(0.025)
Private Commitment X Group density	-0.007*	-0.002	-0.002	0.002
	(0.003)	(0.002)	(0.004)	(0.003)
Public Commitment	0.064*	0.044***	0.071**	0.056***
	(0.034)	(0.015)	(0.028)	(0.017)
Public Commitment X Group density	0.001	0.001	0.000	0.000
	(0.004)	(0.002)	(0.005)	(0.004)
Group density	0.004	0.000	0.000	-0.003
	(0.003)	(0.002)	(0.003)	(0.002)
Baseline share owning hyg. lat.		0.706***		0.513***
		(0.022)		(0.036)
Share of households landless		-0.082***		-0.091***
		(0.017)		(0.031)
Union FEs	Yes	Yes	Yes	Yes
Number of groups	1,236	1,235	1,235	1,234
Number of villages	107	107	107	107
Omitted category mean	0.451	0.451	0.544	0.544
Omitted category S.D.	(0.189)	(0.189)	(0.255)	(0.255)

Notes: the dependent variable is the share of households in the group with a hygienic latrine. Columns (1) and (2) report short-term effects (at the time of assessment); columns (3) and (4) report medium-term effects (12-15 months after assessment). Observations (groups) are weighted by the number of households. The comparison group consists of groups that received the meetings only treatment. Pure control villages are included as a separate category to enhance precision. Standard errors clustered at the village level. Standard errors clustered at the village level in parentheses. * $p < 0.10$, ** $p < 0.05$, *** $p < 0.01$.

Table F9: Program Effects: Hygienic Latrine Ownership
Interacted with Maximum eigenvalue of adjacency matrix

	Short term		Medium term	
	(1)	(2)	(3)	(4)
Monetary Reward	0.122***	0.075***	0.048	0.014
	(0.034)	(0.015)	(0.030)	(0.019)
Monetary Reward X Max. eigval. of adj. matrix	0.004	-0.009	-0.009	-0.017
	(0.015)	(0.009)	(0.014)	(0.013)
Reward Certificate	0.042	0.010	0.043	0.020
	(0.037)	(0.012)	(0.035)	(0.022)
Reward Certificate X Max. eigval. of adj. matrix	0.008	0.002	-0.012	-0.016*
	(0.013)	(0.007)	(0.012)	(0.009)
Private Commitment	0.010	0.009	0.013	0.012
	(0.038)	(0.012)	(0.038)	(0.024)
Private Commitment X Max. eigval. of adj. matrix	-0.009	-0.007	-0.007	-0.005
	(0.011)	(0.006)	(0.012)	(0.010)
Public Commitment	0.065*	0.044***	0.072**	0.055***
	(0.035)	(0.015)	(0.027)	(0.017)
Public Commitment X Max. eigval. of adj. matrix	-0.009	-0.005	0.001	0.005
	(0.015)	(0.008)	(0.013)	(0.011)
Max. eigval. of adj. matrix	-0.007	-0.001	0.010	0.014*
	(0.012)	(0.006)	(0.012)	(0.008)
Baseline share owning hyg. lat.		0.711***		0.514***
		(0.022)		(0.036)
Share of households landless		-0.079***		-0.088***
		(0.017)		(0.031)
Union FEs	Yes	Yes	Yes	Yes
Number of groups	1,234	1,233	1,233	1,232
Number of villages	107	107	107	107
Omitted category mean	0.451	0.451	0.544	0.544
Omitted category S.D.	(0.189)	(0.189)	(0.255)	(0.255)

Notes: the dependent variable is the share of households in the group with a hygienic latrine. Columns (1) and (2) report short-term effects (at the time of assessment); columns (3) and (4) report medium-term effects (12-15 months after assessment). Observations (groups) are weighted by the number of households. The comparison group consists of groups that received the meetings only treatment. Pure control villages are included as a separate category to enhance precision. Standard errors clustered at the village level. Standard errors clustered at the village level in parentheses. * $p < 0.10$, ** $p < 0.05$, *** $p < 0.01$.

Table F10: Program Effects: Hygienic Latrine Ownership
Interacted with Second eigenvalue of stochastized adjacency matrix

	Short term		Medium term	
	(1)	(2)	(3)	(4)
Monetary Reward	0.126***	0.079***	0.046	0.012
	(0.034)	(0.015)	(0.030)	(0.020)
Monetary Reward X Second eignval. of stoch. adj. matrix	0.017	-0.046**	0.036	-0.009
	(0.037)	(0.023)	(0.036)	(0.034)
Reward Certificate	0.042	0.010	0.041	0.018
	(0.037)	(0.012)	(0.036)	(0.023)
Reward Certificate X Second eignval. of stoch. adj. matrix	0.013	-0.010	0.024	0.007
	(0.022)	(0.016)	(0.029)	(0.028)
Private Commitment	0.009	0.009	0.014	0.013
	(0.039)	(0.012)	(0.039)	(0.025)
Private Commitment X Second eignval. of stoch. adj. matrix	0.007	-0.007	0.000	-0.010
	(0.025)	(0.017)	(0.029)	(0.027)
Public Commitment	0.065*	0.046***	0.073**	0.058***
	(0.036)	(0.016)	(0.028)	(0.017)
Public Commitment X Second eignval. of stoch. adj. matrix	-0.010	-0.001	0.004	0.012
	(0.031)	(0.022)	(0.034)	(0.032)
Second eignval. of stoch. adj. matrix	0.000	0.022	0.000	0.015
	(0.020)	(0.018)	(0.025)	(0.020)
Baseline share owning hyg. lat.		0.710***		0.512***
		(0.022)		(0.036)
Share of households landless		-0.082***		-0.089***
		(0.017)		(0.032)
Union FEs	Yes	Yes	Yes	Yes
Number of groups	1,234	1,233	1,233	1,232
Number of villages	107	107	107	107
Omitted category mean	0.451	0.451	0.544	0.544
Omitted category S.D.	(0.189)	(0.189)	(0.255)	(0.255)

Notes: the dependent variable is the share of households in the group with a hygienic latrine. Columns (1) and (2) report short-term effects (at the time of assessment); columns (3) and (4) report medium-term effects (12-15 months after assessment). Observations (groups) are weighted by the number of households. The comparison group consists of groups that received the meetings only treatment. Pure control villages are included as a separate category to enhance precision. Standard errors clustered at the village level. Standard errors clustered at the village level in parentheses. * $p < 0.10$, ** $p < 0.05$, *** $p < 0.01$.

G Single Treatment Villages and Fully Interacted Model

In this section, we present (1) estimates using only the single-treatment villages and (2) fully interacted factorial specifications.

G.1 Single-Treatment Village Estimates

Here, we restrict attention to the villages receiving only one of the treatments (monetary reward, reward certificate, private commitment, public commitment), plus the meetings-only and pure control villages. These correspond to cells A (meetings only), B (private commitment, no reward), C (public commitment, no reward), D (monetary reward, no commitment), G (reward certificate, no commitment), and J (pure controls) from Table 1. In other words, we estimate Equation 1 from the main text but drop villages receiving combined treatments, specifically cells E (monetary reward, private commitment), F (monetary reward, public commitment), H (reward certificate, private commitment), and I (reward certificate, public commitment) from Table 1.

We present the results for the main outcomes of interest, short-term and medium-term hygienic latrine ownership, in Table G1 and Figure G1. The results are noisier, as expected, but generally comparable to those using the full sample. The relative magnitudes of point estimates are the same – in the short term, the point estimate for monetary reward is largest, followed by public commitment; in the medium term, the point estimate for public commitment is largest – and the confidence intervals around each estimate from this restricted sample overlap with those from the full sample. However, the absolute magnitudes of these point estimates are smaller, suggesting some complementarity between treatments.

G.2 Fully Interacted Model

Here, we estimate a fully interacted model, augmenting Equation 1 with separate indicators for each treatment cell.

The estimates are presented in Table G2 and Figure G2. As with the results of Section G.1, there is suggestive evidence of positive interaction effects, although as expected the estimates are imprecise. Specifically, in the short term, the combination of the monetary reward and the public commitment treatment is larger than either of these treatments alone, and these differences are both economically meaningful and statistically significant ($p < 0.01$). However, this relationship does not hold into the medium term, when the combination of monetary incentive and public commitment does no better than the public commitment alone. Interestingly, in the medium term, the point estimate for the combination of public commitment and reward certificate (i.e., non-monetary reward) is greatest, which is consistent with the mechanism of signalling and reputation we discuss in the main text. However, given the imprecision of our estimates for individual cells, we view this only as suggestive.

Table G1: Program Effects: Hygienic Latrine Ownership
Single Treatment Villages

	Short term		Medium term	
	(1)	(2)	(3)	(4)
Reward Certificate Only	-0.011	-0.018	-0.009	-0.014
	(0.049)	(0.025)	(0.040)	(0.025)
	[-0.133, 0.109]	[-0.084, 0.044]	[-0.103, 0.089]	[-0.067, 0.046]
Monetary Reward Only	0.069*	0.036	0.011	-0.013
	(0.041)	(0.026)	(0.039)	(0.031)
	[-0.022, 0.164]	[-0.031, 0.093]	[-0.079, 0.104]	[-0.101, 0.052]
Private Commitment Only	-0.034	-0.021	-0.023	-0.015
	(0.040)	(0.024)	(0.046)	(0.040)
	[-0.132, 0.056]	[-0.083, 0.036]	[-0.134, 0.091]	[-0.100, 0.100]
Public Commitment	-0.005	0.009	0.014	0.023
	(0.043)	(0.022)	(0.033)	(0.024)
	[-0.093, 0.125]	[-0.043, 0.066]	[-0.053, 0.106]	[-0.028, 0.080]
Baseline share owning hyg. lat.		0.688***		0.479***
		(0.028)		(0.043)
Share of households landless		-0.075***		-0.100***
		(0.023)		(0.038)
Union FEs	Yes	Yes	Yes	Yes
Number of groups	847	846	847	846
Number of villages	75	75	75	75
Omitted category mean	0.399	0.401	0.540	0.543
Omitted category S.D.	(0.240)	(0.240)	(0.249)	(0.247)

Notes: the dependent variable is the share of households in the group with a hygienic latrine Columns (1) and (2) report short-term effects (at the time of assessment); columns (3) and (4) report medium-term effects (12-15 months after assessment).. Observations (groups) are weighted by the number of households. The sample includes villages that received only one treatment, plus meetings-only villages and pure controls. Meetings-only villages are the omitted category. Pure control villages are included for enhanced precision. Standard errors clustered at the village level. * $p < 0.10$, ** $p < 0.05$, *** $p < 0.01$.

Figure G1: Program Effects: Hygienic Latrine Ownership
Single Treatment Villages

(a) Short-term

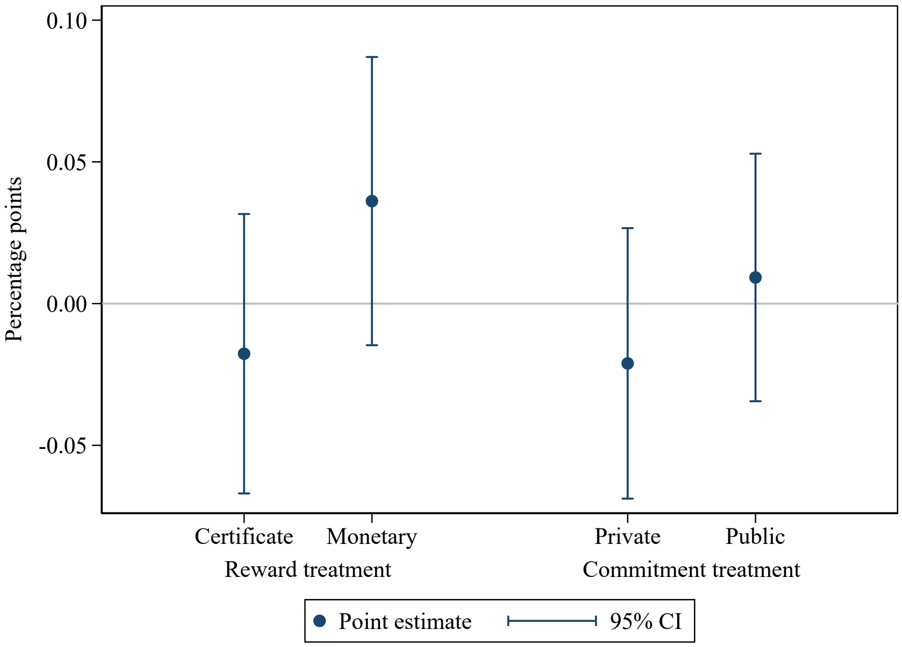

(b) Medium-term

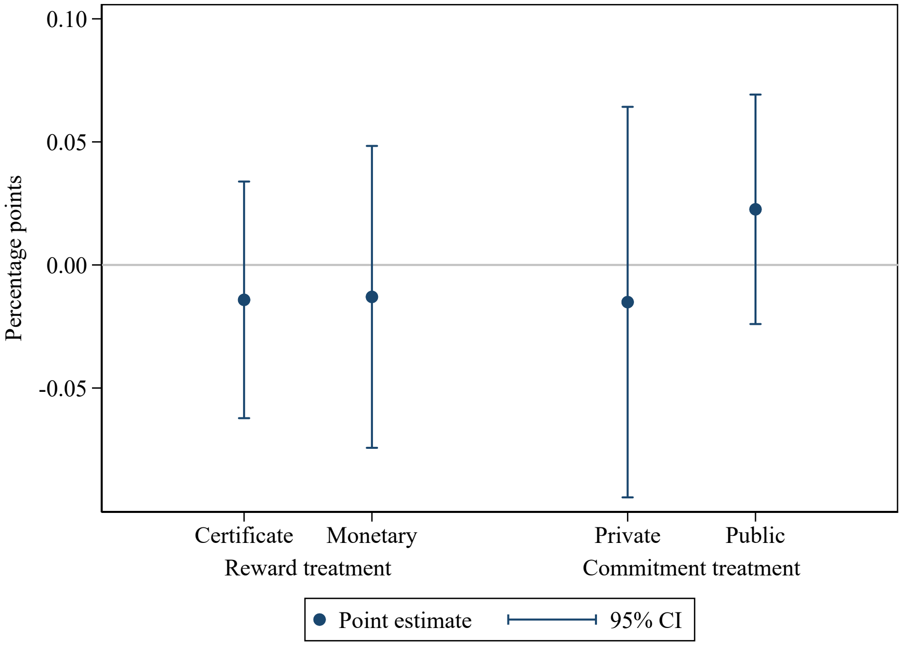

Notes: this graph presents estimated treatment effects of the interventions on the share of households in the group with a hygienic latrine. Panel (a) presents effects in the short term (at the time of assessment); panel (b) in the medium term (12-15 months after assessment). The regression controls for the baseline level of the outcome variable, the share of households in the group that are landless, and union fixed effects. Observations (groups) are weighted by the number of households. The sample includes villages that received only one treatment, plus meetings-only villages and pure controls. Meetings-only villages are the omitted category. Pure control villages are included for enhanced precision. 95% confidence intervals use standard errors clustered at the village level (the level of randomization).

Table G2: Program Effects: Hygienic Latrine Ownership
Fully Interacted Treatments

	Short term		Medium term	
	(1)	(2)	(3)	(4)
Monetary reward only	0.078*	0.040	0.018	-0.011
	(0.042)	(0.025)	(0.038)	(0.030)
	[-0.015, 0.176]	[-0.025, 0.094]	[-0.067, 0.115]	[-0.094, 0.049]
Monetary reward & private commitment	0.122***	0.094***	0.043	0.022
	(0.046)	(0.030)	(0.052)	(0.039)
	[0.009, 0.266]	[0.020, 0.194]	[-0.073, 0.209]	[-0.070, 0.162]
Monetary reward & public commitment	0.191***	0.114***	0.091**	0.031
	(0.058)	(0.026)	(0.041)	(0.021)
	[0.015, 0.302]	[0.046, 0.170]	[-0.028, 0.176]	[-0.020, 0.076]
Certificate only	-0.014	-0.016	-0.013	-0.015
	(0.054)	(0.025)	(0.044)	(0.024)
	[-0.142, 0.121]	[-0.078, 0.044]	[-0.120, 0.092]	[-0.067, 0.044]
Certificate & private commitment	0.087	0.017	0.057	0.004
	(0.057)	(0.023)	(0.061)	(0.032)
	[-0.078, 0.201]	[-0.046, 0.074]	[-0.103, 0.183]	[-0.083, 0.068]
Certificate & public commitment	0.099**	0.047*	0.105***	0.064***
	(0.046)	(0.026)	(0.031)	(0.024)
	[-0.012, 0.218]	[-0.018, 0.109]	[0.033, 0.175]	[0.010, 0.118]
Private commitment only	-0.040	-0.020	-0.031	-0.017
	(0.041)	(0.023)	(0.046)	(0.040)
	[-0.140, 0.051]	[-0.078, 0.035]	[-0.139, 0.082]	[-0.099, 0.100]
Public commitment only	0.005	0.012	0.023	0.027
	(0.042)	(0.022)	(0.034)	(0.025)
	[-0.082, 0.137]	[-0.040, 0.067]	[-0.050, 0.122]	[-0.030, 0.090]
Baseline share owning hyg. lat.		0.701***		0.512***
		(0.023)		(0.036)
Share of households landless		-0.082***		-0.088***
		(0.018)		(0.032)
Union FEs	Yes	Yes	Yes	Yes
Diff.: Monetary & Public − Monetary Only	0.112	0.074	0.073	0.043
	(0.063)	(0.027)	(0.049)	(0.029)
p-value	0.078	0.007	0.138	0.150
Diff.: Monetary & Public − Public Only	0.185	0.102	0.068	0.004
	(0.062)	(0.025)	(0.044)	(0.024)
p-value	0.003	0.000	0.130	0.864
Number of groups	1,236	1,235	1,235	1,234
Number of villages	107	107	107	107
Omitted category mean	0.451	0.451	0.544	0.544
Omitted category S.D.	(0.189)	(0.189)	(0.255)	(0.255)

Notes: the dependent variable is the share of households in the group with a hygienic latrine Columns (1) and (2) report short-term effects (at the time of assessment); columns (3) and (4) report medium-term effects (12-15 months after assessment).. Observations (groups) are weighted by the number of households. The comparison group consists of groups that received the meetings only treatment. Pure control villages are included as a separate category to enhance precision. Standard errors clustered at the village level in parentheses. Wild cluster bootstrap (9,999 repetitions, Webb weights) 95% confidence intervals, resampling at the village level, in brackets for the coefficients of interest. * $p < 0.10$, ** $p < 0.05$, *** $p < 0.01$.

Figure G2: Program Effects: Hygienic Latrine Ownership
Fully Interacted Treatments

(a) Short-term

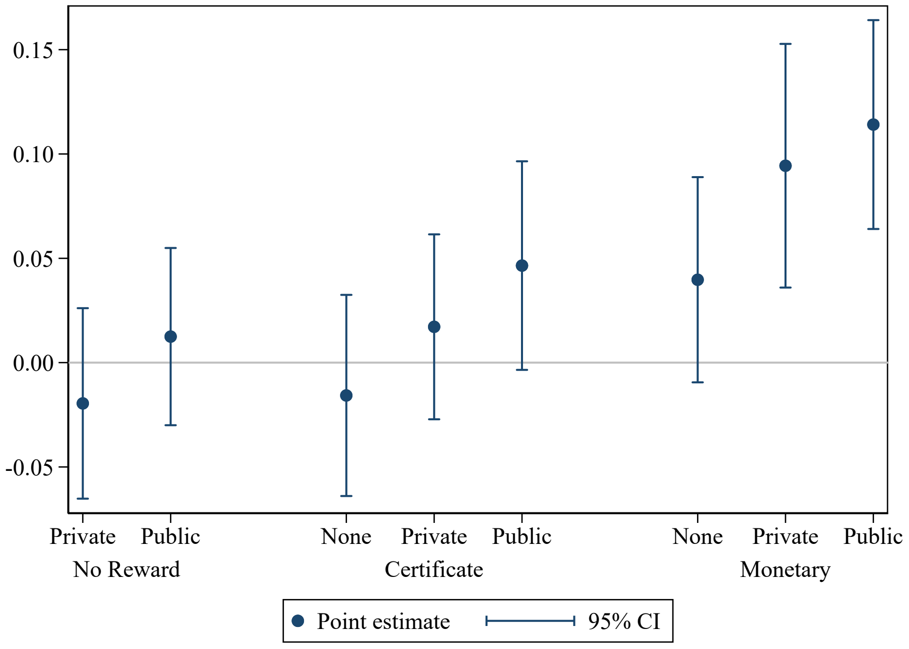

(b) Medium-term

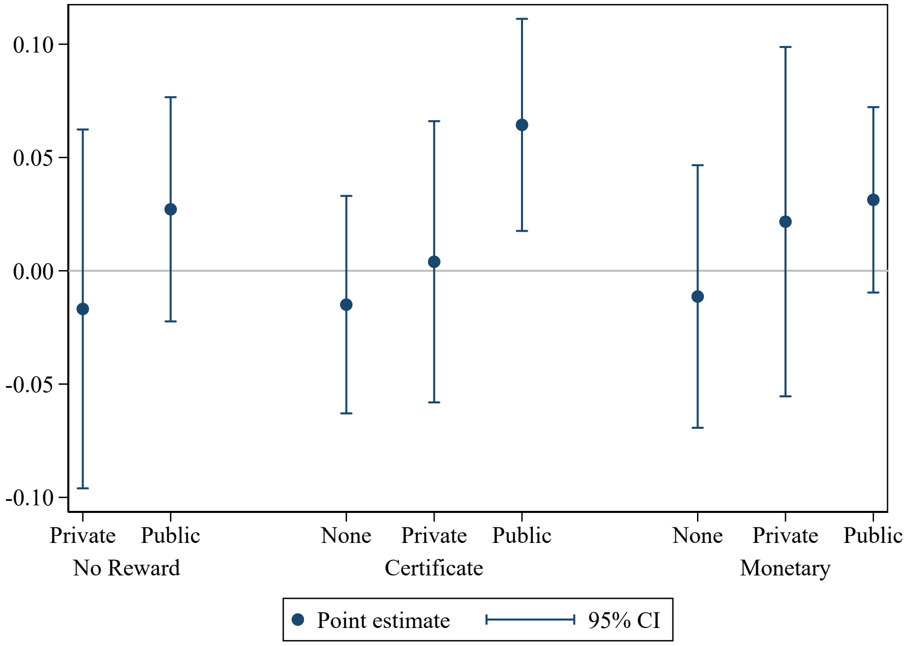

Notes: this graph presents estimated treatment effects of the interventions on the share of households in the group owning a hygienic latrine. Panel (a) presents effects in the short term (at the time of assessment); panel (b) in the medium term (12-15 months after assessment). The regression controls for the baseline level of the outcome variable, the share of households in the group that are landless, and union fixed effects. Observations (groups) are weighted by the number of households. The comparison group consists of groups that received the meetings only treatment. Pure control villages are included as a separate category to enhance precision. 95% confidence intervals use standard errors clustered at the village level (the level of randomization).

Appendix References

Gibbons, C. E., S. J. C. Suárez, and M. B. Urbancic (2019). "Broken or Fixed Effects?" *Journal of Econometric Methods* 8.1. DOI: 10.1515/jem-2017-0002.

Lin, W. (2013). "Agnostic notes on regression adjustments to experimental data: Reexamining Freedman's critique." *The Annals of Applied Statistics* 7.1, pp. 295–318. DOI: 10.1214/12-AOAS583.

Wainer, H. (2000). "The Centercept: An Estimable and Meaningful Regression Parameter." *Psychological Science* 11.5, pp. 434–436. DOI: 10.1111/1467-9280.00284.

Social and Financial Incentives for Overcoming a Collective Action Problem

Supplementary Materials

M. Mehrab Bakhtiar†
IFPRI

Raymond Guiteras
North Carolina State University

James Levinsohn
Yale University

Ahmed Mushfiq Mobarak
Yale University

January 2023

†Email: m.bakhtiar@cgiar.org, rpguiter@ncsu.edu, james.levinsohn@yale.edu, ahmed.mobarak@yale.edu.

SM1 Group Formation

As noted in Section 2, the sample consisted of households living in villages that were part of the previous demand study. In that study, villages were subdivided into neighborhoods, and that first set of interventions was conducted at the neighborhood level.[1] The median number of households per neighborhood was 40 (IQR 26-56), and the median number of neighborhoods per village was 4 (IQR 2-6).

For this study, a somewhat smaller intervention unit was appropriate because our fieldwork and qualitative background-information gathering suggested that having households making public commitments to smaller groups of immediate neighbors was more sensible, and that there would be less free-riding and meeting non-attendance in smaller groups. Therefore, we further divided neighborhoods into "groups": sets of 15-20 roughly contiguous households within the neighborhood.

The intervention supervisors who had also been involved with the first set of interventions – the "demand study interventions" – were tasked with the process of assigning households to groups. Because of their long stay in the survey area, the supervisors had developed a close understanding of the socio-dynamics of the sample. This helped them form groups without breaking any organic link between clusters of households in a neighborhood. The general instructions for constructing groups were:

1. Groups should consist of around 15 continuous / neighboring households;
2. Groups should generally not exceed 20 households

Generally, natural divisions such as rivers or open space between households were used to assign households into simple, contiguous groups. However, some exceptions were made based on practical constraints, in particular when:

1. Households were located in isolated places. If the number of such isolated households was too few to form their own group (say, only 1-2 households) they were added to the nearest group. On the other hand, if the number of such nearby households was higher but still less than 15, these households were put together to form a group. Ultimately, only 6 groups of less than 10 households were formed.
2. At the other extreme, sometimes households were very densely packed and it proved difficult to separate them meaningfully into discrete groups. The largest group in the study area consists of 33 households. Ultimately, only 3 groups consisted of more than 23 households.

[1] The neighborhood, or *para* in Bangla, is not a formal or official designation, but unofficial neighborhood boundaries were usually common knowledge in the community, and in these cases we followed local convention. If there were not well-defined neighborhoods in a village, or if a neighborhood needed to be divided because of its size, we used natural divisions such as rivers or roads where such existed. If such natural pre-existing divisions did not exist or were not practical, we created "neighborhoods" (for the purpose of the study) of households in simple, contiguous sets.

These groups included all households in the villages, including new households that had been formed since the beginning of the demand study.

SM2 Key Components of Hygienic Latrines

Figure SM2-1: Pour-flush latrine with offset sealed pit: overview

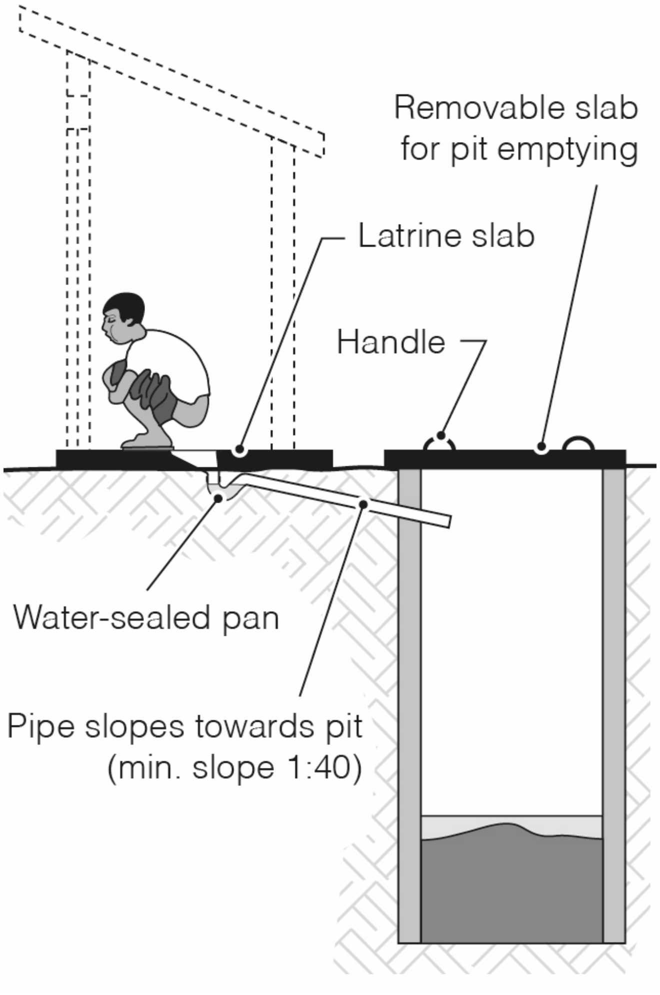

Notes: from Reed (2014). The key features are the slab, water seal and the offet sealed pit.

Figure SM2-2: Pour-flush latrine: pan and water seal

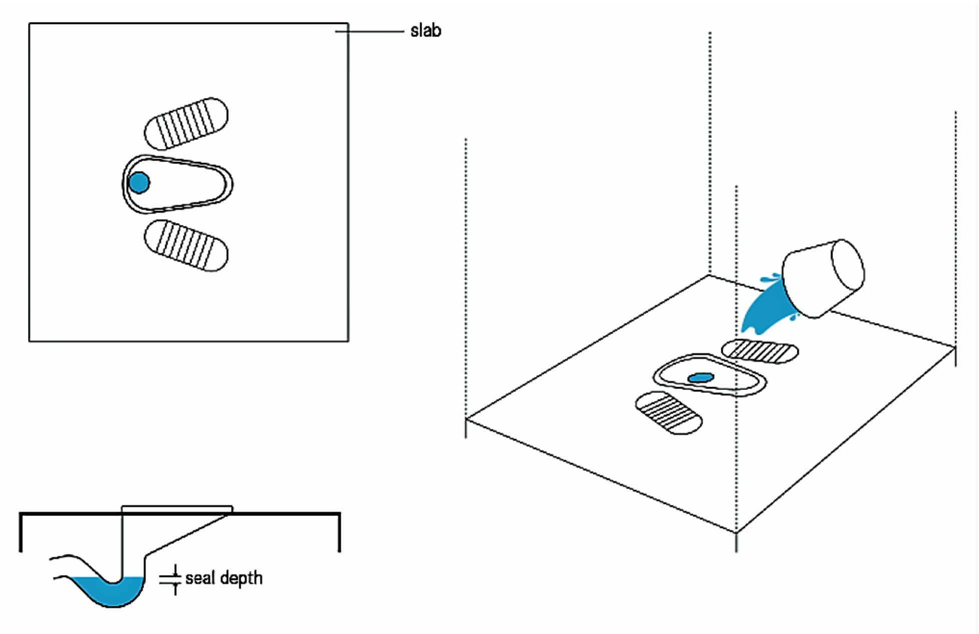

Notes: from Tilley et al. (2014). The water seal blocks flies from accessing the pit and blocks odors from escaping the pit.

Figure SM2-3: Sealed pit

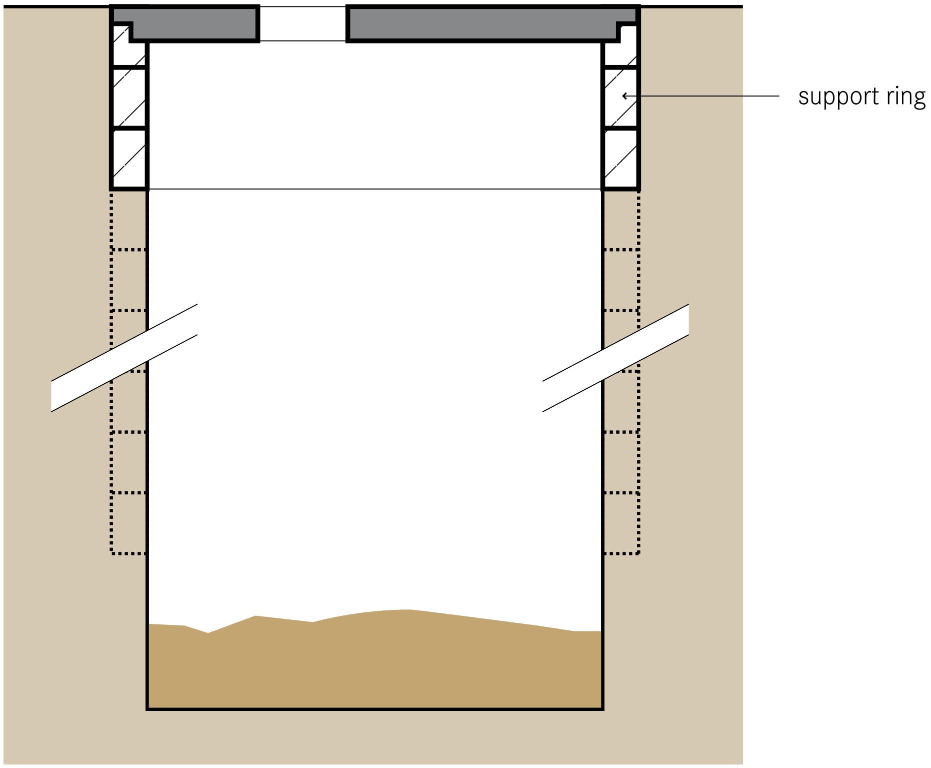

Notes: from Tilley et al. (2014). The sealed rings at the top of the pit reinforce against collapse and prevent surface water from entering the pit. Concrete rings are appropriate for the wet conditions of the study site. The lower section is lined, often with bricks in a honeycomb pattern, to collect solid waste while allowing liquid waste to seep into the ground.

Figure SM2-4: Twin Pit Latrine

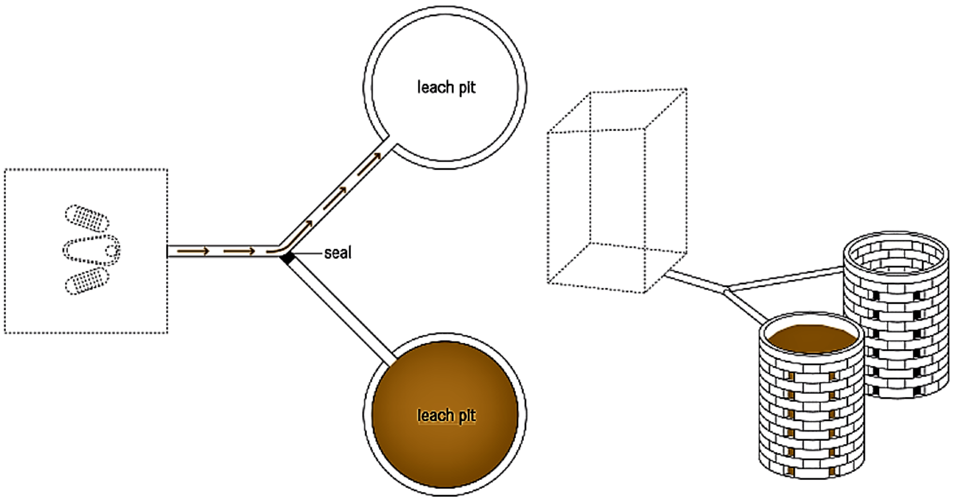

Notes: from Tilley et al. (2014). The twin pit design allows one pit to be sealed off when full and the other pit to be put in use. After several months of decomposition, the material in the first pit will have decomposed, making pit emptying no longer hazardous or unpleasant.

SM3 Outcome Variables

Here, we provide detail on how our outcome variables are constructed from our survey instruments. See Sections 3.2 and 4 for discussion of the concepts underlying these definitions.

Household-level variables

Short-term outcomes

- *Open defecation:*

  Survey Section 1, Q4, "What kind of facility is the latrine most regularly used (primary latrine) by the household?" Surveyor observes facility. Response 01 "Don't have any latrine / Open defecation" or 02 "Hanging latrine" coded as open defection.

  Because the short-term data collection was part of the assessment for rewards, self-report of open defecation was especially likely to be biased by treatment arm. Therefore, in this survey round, we use only OD as inferred from the surveyor's assessment of the facility the household reports as its primary latrine or defecation site.

- *Any Latrine Access:*

  Survey Section 1, Q4, "What kind of facility is the latrine most regularly used (primary latrine) by the household?" Surveyor observes facility. Response 01 "Don't have any latrine / Open defecation" or 02 "Hanging latrine" coded as not having access to a latrine, while any other response for this question - ranging from Response 03 "Open Pit/hole without slab and lid or cover" to Response 17 "Sanitary latrine with septic tank" - was coded as having access to a latrine.

- *Any Latrine Ownership:*

  Latrine ownership is defined as the sole or joint ownership of the household's primary latrine facility. Ownership is a strict subset of access – households without access to a latrine are coded as not owning a latrine facility.

  Survey Section 1, Q5, "What is the ownership status of the primary latrine? CODE: 01=Toilet jointly owned with another household, 02=Toilet solely owned by household, 03=community toilet, 04=owned by others/neighbor". Response 01 or Response 02 was coded as owning a latrine, while any other response was coded as not owning a latrine facility.

- *Hygienic Latrine Access:*

  A latrine that a household has access to (see above) is classified as hygienic if satisfies all three of the following criteria, which attempt to capture whether the latrine safely separates feces from the environment: (1) has an intact and functional slab; (2) has an

intact and functional water seal; and (3) does not have any observable leak from the pit or any other latrine component (such as the pipe or Y-junction).

Whether a latrine has an intact and functional slab is based on two questions. The first is Section 1, Q24, "Type of the latrine slab CODE: 00=No slab, 01=Concrete/cement, 02=Plastic, 03=Bamboo, 04=Brick, 05=Earthen, 06=Others". The second is Section 1, Q25, "What is the current condition of latrine slab? CODE: 01=Fully Intact, 02=Partially Broken, 03=Completely Broken". Response 00 for Q24 or Responses 02 or 03 for Q25 leads a latrine to be coded to not have a functional slab.

Whether a latrine has an intact and functional water seal is based on two questions. The first is Section 1, Q27, "What is the current condition of the water seal? CODE: 01=Fully Intact, 02=Partially Broken, 03=Completely Broken, 04=No water seal". A latrine is coded to have a functional/intact water seal with Response 01 to this question. On the other hand, a latrine is coded to not have a functional water seal for Responses 02, 03 and 04. The second is Section 1, Q4, "What kind of facility is the latrine most regularly used (primary latrine) by the household?" Response 01 "Don't have any latrine / Open defecation" or 02 "Hanging latrine" or 03 "Open Pit/hole without slab and lid or cover", as well as 08 "Ring-slab latrine (direct) with water seal:broken/none" or 11 "Single pit ring-slab latrine (Offset) with water seal: broken" or 14 "Double pit Ring-slab latrine (Offset) with water seal: broken", leads a latrine to be coded to not have a functional water seal.

Whether a latrine has components without any observed leaks is based on three questions. The first is Section 1, Q19a "(OBSERVE) Is there any leakage of the latrine pipe, Y junction, pit or the tank? Code: 01=Major, 02=Minor, 03=No leak". A response of 01 or 02 for this question leads a latrine to be coded to have a leaking component. The second is Section 1, Q18, "Is the latrine pipe linked to any ditch/canal/pond etc.? CODE: 01=Yes, 02=No". A response of 01 for this question leads a latrine to be coded to have a leaking component. The third is Section 1, Q4 is also used "What kind of facility is the latrine most regularly used (primary latrine) by the household?" . Response 01 "Don't have any latrine / Open defecation" or 02 "Hanging latrine" or "03. Open Pit/hole without slab and lid or cover" leads a latrine to be coded to have a leaking component.

- *Hygienic Latrine Ownership:*

  Hygienic latrine ownership is defined as the sole or joint ownership of a hygienic latrine facility a household has access to. Hygienic ownership is a strict subset of hygienic access – households without access to any latrine or a hygienic latrine are coded as not owning a hygienic latrine.

  Survey Section 1, Q5, "What is the ownership status of the primary latrine? CODE: 01=Toilet jointly owned with another household, 02=Toilet solely owned by household, 03=community toilet, 04=owned by others/neighbor". If a household has access to a hygienic latrine (see above), Response 01 or Response 02 is then coded as owning a hygienic latrine, while any other response was coded as not owning a hygienic latrine facility.

Medium-term outcomes

The medium-term outcomes are defined identically to those collected in the short term, except for household self-reported open defecation, which was not collected in the short term. There are slight differences in question and response numbering, so we include the definitions below in spite of the redundancy.

- *Open defecation:* Survey Section H, Q12, "What kind of facility is the latrine most regularly used (primary latrine) by the household?" Surveyor observes facility. Response 01 "Don't have any latrine / Open defecation" or 02 "Hanging latrine" coded as open defection.
- *Open defecation among adults (household self-report):* Survey Section H, Q7, "Do (adult men, adult women, children) use open spaces / bushes / hanging latrines for defecation?" Coded Yes if respondent answered Yes (01) for adult men or adult women.

  This measure was not collected in the short-term survey, since open defecation was connected to rewards and response bias would therefore likely be correlated with treatment.
- *Any Latrine Access:*

  Survey Section H-1, Q13, "What kind of facility is the latrine most regularly used (primary latrine) by the household?" Surveyor observes facility. Response 01 "Don't have any latrine / Open defecation" or 02 "Hanging latrine" coded as not having access to a latrine, while any other response for this question - ranging from Response 03 "Open Pit/hole without slab and lid or cover" to Response 17 "Sanitary latrine with septic tank" - was coded as having access to a latrine.
- *Any Latrine Ownership:*

  Latrine ownership is a strict subset of latrine access and is defined as the sole or joint ownership of the latrine facility a household has access to.

  Section H-1, Q18, "What is the ownership status of the primary latrine? CODE: 01=Toilet jointly owned with another household, 02=Toilet solely owned by household, 03= Other's toilet". Response 01 or Response 02 was coded as owning a latrine, while any other response was coded as not owning a latrine facility. Not having access to a latrine is also coded as not owning a latrine facility.
- *Hygienic Latrine Access:*

  A latrine that a household has access to (see above) is classified as hygienic if satisfies all three of the following criteria: (1) has an intact and functional slab; (2) has an intact and functional water seal; and (3) does not have any observable leak from the pit or any other latrine component (such as the pipe or Y-junction).

  Whether a latrine has an intact and functional slab is based on two questions. The first is Section H-1, Q46, "Type of the latrine slab CODE: 00=No slab, 01=Concrete/cement,

02=Plastic, 03=Bamboo, 04=Brick, 05=Earthen, 06=Others". The second is Section H-1, Q47, "What is the current condition of latrine slab? CODE: 01=Fully Intact, 02=Partially Broken, 03=Completely Broken". Response 00 for Q24 or Responses 02 or 03 for Q25 leads a latrine to be coded to not have a functional slab. Not having access to a latrine also leads this variable to be coded as zero.

Whether a latrine has an intact and functional water seal is based on two questions. The first is Section H-1, Q50, "What is the current condition of the water seal? CODE: 01=Fully Intact, 02=Partially Broken, 03=Completely Broken, 04=No water seal". A latrine is coded to have a functional/intact water seal with Response 01 to this question. On the other hand, a latrine is coded to not have a functional water seal for Responses 02, 03 and 04. The second is Section H-1, Q13, "What kind of facility is the latrine most regularly used (primary latrine) by the household?" Response 01 "Don't have any latrine / Open defecation" or 02 "Hanging latrine" or 03 "Open Pit/hole without slab and lid or cover", as well as 08 "Ring-slab latrine (direct) with water seal:broken/none" or 11 "Single pit ring-slab latrine (Offset) with water seal: broken" or 14 "Double pit Ring-slab latrine (Offset) with water seal: broken", leads a latrine to be coded to not have a functional water seal.

Whether a latrine has functional components without any observed leaks is based on three questions. The first is Section H-1, Q36a, "(OBSERVE) Is there any leakage of the latrine pipe, Y junction, pit or the tank? Code: 01=Major, 02=Minor, 03=No leak". A response of 01 or 02 for this question leads a latrine to be coded to have a leaking component. The second is Section H-1, Q35, "Is the latrine pipe linked to any ditch/canal/pond etc.? CODE: 01=Yes, 02=No". A response of 01 for this question leads a latrine to be coded to have a leaking component. The third is Survey Section H-1, Q13, "What kind of facility is the latrine most regularly used (primary latrine) by the household?" Response 01 "Don't have any latrine / Open defecation" or 02 "Hanging latrine" or "03. Open Pit/hole without slab and lid or cover" leads a latrine to be coded to have a leaking component.

- *Hygienic Latrine Ownership:*

  Hygienic latrine ownership is defined as the sole or joint ownership of a hygienic latrine facility a household has access to. Hygienic ownership is a strict subset of hygienic access – households without access to any latrine or a hygienic latrine are coded as not owning a hygienic latrine.

  Section H-1, Q18, "What is the ownership status of the primary latrine? CODE: 01=Toilet jointly owned with another household, 02=Toilet solely owned by household, 03= Other's toilet". If a household has access to a hygienic latrine (see above), Response 01 or Response 02 is then coded as owning a hygienic latrine, while any other response was coded as not owning a hygienic latrine facility.

Group shares

In all cases, group shares are the fraction of households surveyed in the group in the corresponding category. As discussed in Section 4 of the main text, the endline survey was conducted with a 50% subsample stratified by village, leading to some imbalance in the number of households surveyed per group. For groups with fewer than 6 households selected for the endline survey, we randomly selected a “top-up” sample from the remaining households and conducted a brief followup consisting of the endline’s modules on latrine condition and use.

Followup-02/Final Cluster Meeting Training Manual

Step-01: On the day before the cluster meeting, the selected leader will be called on mobile so that s/he tells all other members to be present at the meeting on time.

Step-02: After reaching a cluster, at first the Health Motivator will go to each house of the cluster, exchange greetings and invite them to join the meeting at a selected place. Then s/he will inspect the household's latrine/s and collect the information on the electronic tabs. If the latrine is unhygienic (based on the criteria listed below) then it must be explained to the household what steps they need to take in order to make the latrine hygienic.

<table>
<tr><td colspan="4">NOTE: Please ask the follow questions on the primary latrine which is the toilet facility that is used by the household members the majority of the time at the period during which the survey is being conducted.</td></tr>
<tr><td rowspan="9">1</td><td rowspan="9">What kind of facility is the primary latrine used by the household?</td><td>01. Don't have any latrine/Open defecation</td><td>10. Single pit Ring-slab latrine (Offset) with water seal: intact</td></tr>
<tr><td>02. Hanging latrine</td><td>11. Single pit ring-slab latrine (Offset) with water seal: broken</td></tr>
<tr><td>03. Open Pit/hole without slab and lid or cover</td><td>12. Single pit Ring-slab latrine (Offset) with flip/ pollythene</td></tr>
<tr><td>04. Pit latrine with slab but without lid or cover</td><td>13. Double pit Ring-slab latrine (Offset) with water seal: intact</td></tr>
<tr><td>05. Pit latrine with cover</td><td>14. Double pit Ring-slab latrine (Offset) with water seal: broken</td></tr>
<tr><td>06. Modern pit latrine with vent pipe</td><td>15. Double pit Ring-slab latrine (Offset) with flip/ pollythene</td></tr>
<tr><td>07. Ring-slab latrine (direct) with water seal: intact</td><td>16. Eco Latrine</td></tr>
<tr><td>08. Ring-slab latrine (direct) with water seal: broken/none</td><td>17. Sanitary latrine with septic tank</td></tr>
<tr><td>09. Ring-slab latrine (direct) with flip/ pollythene</td><td>|___|___|</td></tr>
<tr><td>2</td><td colspan="2">What is the ownership status of the primary latrine? CODE: 01=Toilet jointly owned/shared with another household, 02=private toilet solely owned by household, 03=community toilet, 04=uses someone else's latrine, 05=open spaces/bushes/hanging latrine</td><td>|___|___|</td></tr>
<tr><td>3</td><td colspan="2">Where is the primary latrine located? CODE: 01=In own homestead (attached), 02=Outside own homestead (not attached), 03=Community latrine, 04=Another household/neighbor's latrine, 05=open defecation</td><td>|___|___|</td></tr>
<tr><td>4</td><td colspan="2">How many other households share this latrine facility?</td><td>|___|___|</td></tr>
<tr><td>5</td><td colspan="2">What is the current condition of latrine slab? CODE: 01=Fully Intact, 02=Partially Broken, 03=Completely Broken, 04=No slab, 05=N/A</td><td>|___|___|</td></tr>
<tr><td>6</td><td colspan="2">Is there any visual evidence of <u>lumps of feces</u> in the toilet area? (does <u>not</u> apply for traces or floating fecal matter) CODE: 01 = On Pan; 02 = On Slab, 03 = On both Pan and slab, 04 =No fecal matter seen, 05=N/A</td><td>|___|___|</td></tr>
<tr><td>7</td><td colspan="2">What is the current condition of the water seal? CODE: 01=Fully Intact, 02=Partially Broken, 03=Completely Broken, 04= No Water Seal, 05=N/A</td><td>|___|___|</td></tr>
<tr><td>8</td><td colspan="2">(If offset) What is the current condition of delivery pipe? CODE: 01=Fully Intact, 02=Partially Broken, 03=Completely Broken, 04= No Delivery Pipe, 05=N/A</td><td>|___|___|</td></tr>
<tr><td>9</td><td colspan="2">(If offset) What is the current condition of pit cover? CODE: 01=Fully Intact, 02=Partially Broken, 03=Completely Broken, 04= No Pit Cover, 05=N/A</td><td>|___|___|</td></tr>
<tr><td>10</td><td colspan="2">Are there rings in the latrine pit? CODE: 01=Sufficient no. of Rings, 02=Insufficient No. of Rings, 03=No rings, 05=N/A</td><td>|___|___|</td></tr>
</table>

Notes for data collection:

1. Information has to be entered for hanging latrines
2. If latrine code is 03 then the response to Question 6 is 05/NA
3. If the latrine code is 08 and there is no evidence of feces lumps , then the response to Question 6 will be 04 or 'No fecal matter seen'
4. For hanging latrine (leaking latrine) even if the condition of the ring and pit cover is good, the response to Question-09 will be 02 or 'Partially broken'

5. Need to explain to the household that visible fecal matter makes the latrine unhygienic
6. We will also monitor rings to determine hygienic status – this should be explained
7. If the pit doesn't have sufficient number of rings, the latrine will be deemed unhygienic

Step-03: The meeting will start only when three-fourth of all the members of the cluster are present. The meeting will start by giving thanks to everyone. Everyone will introduce her/himself.

Step-04: Discussion on working as a group:

A. Purpose behind forming groups and solving a problem collectively:

- Unhygienic latrine - feces will be visible, spread bad smell and exposed to flies and insects. Extensive discussion later.
- Unhygienic latrines and unhygienic practices are a social/collective problem. If someone has an unhygienic latrine then it will adversely impact others as well. For example - (pointing finger to someone from the group) "Because of your unhygienic latrine someone else/someone else's children (pointing fingers to someone else) might get sick with cholera, typhoid, diarrhea, jaundice and polio. It is likely to bring economic, physical loss to you and your children."
- For this reason having hygienic latrine for one's own household is not enough, neighbors must have hygienic latrines as well.
- "Whose problem is this? Is this your problem? Does this problem need to be solved in a collective manner?" Because of the enormity of this problem, it needs to be solved in a collective manner.
- "Unity is strength" (Similar Bangla phrases to put emphasis on group work)

B. Read out the names of the members of the group and put emphasis on the importance of spreading the information to those who are absent.

Step-05: Health Motivators will start the discussion on the topics detailed below. Characteristics of a hygienic latrine needs to be repeated as many times as required. Discussion on what is on this script must end within 10-12 minutes.

A. DEFINITION, CHARACTERISTICS AND USE OF HYGIENIC LATRINES

<u>Broad definition of hygienic latrine</u>
1. Hygienic latrines limit the spread of diseases caused by water/feces and keep the environment pollution free.
2. Feces is enclosed in one place and it can not be seen from outside.
3. Flies or other insects cannot enter into the pit.

<u>Most Important technical characteristics of a hygienic latrine:</u>

(These are the characteristics which form the basis for judging if a latrine is hygienic or not. Repeat these characterisitcs often in the cluster meeting so that particiapnts can internalize these attributes.)

1. There must be a slab and it cannot be broken.
2. There must be a water-seal (gooseneck or siphon) and it cannot be broken.
3. There must be an adequate number of rings depending upon the depth of the well and those must not be broken.
4. There should be no gap between the cover of the slab and ring or between the ring and pit cover.
5. There should be no feces, flies or bad smell in or around the latrine.
6. Delivery pipe, y-junction, pit cover cannot be broken.
7. The latrine cannot be connected to a lake or any enclosed water body/ environment cannot be polluted.
8. There should be earth/cement moulded around the slab in case of direct pit latrines.

(Try to get these characteristics repeated by some of the participants)

Methods of using a hygienic latrine:

1. The pan should be made wet by using little water before each use. Feces will not stick on the pan if this is done
2. 1-2 pots (few liters) of water should be used after using the latrine
3. No solid object (like- stone, cloth, mud) should be thrown into the pan
4. The pan of the latrine and the slab/floor must be cleaned everyday.
5. If the latrine pit becomes full than another latrine must be built or the pit must be emptied.

B. Discuss about the number of hygienic/unhygienic latrines among the cluster members and their current condition. Disucuss what must be done to turn the current unhygienic latrines of the clusters into hygienic ones.

C. Targeted number of latrines, deadline and rewards (where applicable) as set by the office must be repeated infront of everybody present so that they can understand it clearly. Make sure that everyone has understood it by asking one or two of them.

D. Motivate each of the households to create and maintain separate hygienic latrine. It must be ensured that they understand that only one household will be rewarded (where applicable) for one hygienic latrine.

[Note: *Cluster meeting participants cannot be shamed.* During household latrine visit if the latrine is found to be unhygienic then the Health Motivator must explain the resaons as to why the latrine is unhygienic. But during the meeting the household member can never be pointed out and shamed. If any household member present in the meeting asks something about her latrine then her questions must be answered while visiting her home at the end of the meeting. However, if any participant asks a question which is applicable for all the participants then that answer can be given in front of everyone. For example if someone asks, "I have not built a separate cover for my offset latrine. But I have covered it up nicely with a carpet (*chatai* in Bangla). Is this hygienic?" answer to this sort of questions can be given in front of all the participants as everyone needs to know about this.]

Step-06: Like the initial meeting, the Health Motivator will conduct a group commitment session (for public commitment intervention) where people will commit to achieve the target. In case of private commitment, the Health Motivators will go to each household separately and make people commit privately.

Commitment

Public Commitment: During each cluster meeting, members from all the households of a cluster will commit publicly that those who do not yet have hygienic latrines will meet hygienic latrine standards as set by the project. Those with hygienic latrines would promise that they would help others reaching the goal within the time limit set by the project. The script of the pledge is as follows:

"I hereby promise before everyone present that I will do my best to set up hygienic latrines or improve existing ones into hygienic latrines for myself and for my neighbors by [end date]."

Private Commitment: After organizing the cluster meeting, health motivators will visit each household in the cluster. The member of the household who had previously attended the meeting will make a commitment before the health motivator that he/she will transform their unhygienic latrines to hygienic ones within the time limit set by the project. The script of the pledge is as follows:

"I hereby promise that I will do my best to set up hygienic latrines or improve existing ones into hygienic latrines for myself and for my surrounding neighbors by [end date]."

Reward Components

There are two threshold targets fixed for clusters, which is in terms of ownership of hygienic latrines. The lower threshold is 25% for [Union 1] while it is 33% for rest of the unions. The higher threshold is 50% for [Union 1] and 66% for the rest of the unions.

Monetary Incentive: A household will get a monetary reward of Tk. 250 or Tk. 500, depending on whether the household owns a hygienic latrine and the ownership of hygienic latrine at the cluster level is above the lower or the upper threshold, respectively.

Certificate: A household will get a certificate of hygiene attainment by a Member or the Chairman of the union, depending on whether the household owns a hygienic latrine and the ownership of hygienic latrine at the cluster level is above the lower or the upper threshold, respectively.

At the end of the meeting, the name of the members of the group should be repeated again and the cluster leader should be handed a complete list containing names of all the members of the cluster so that the group has a better understanding of its members.

Step-07: At the end of the meeting the Health Motivator will briefly recap the whole discussion from beginning to end and give thanks to everyone.

Decisions:

0. Every cluster must be treated the same way (except for the differences arising from the difference in treatment type).

1. Census form has to be filled up in case of new members of the cluster. But add format does not need to be filled for this round.

2. The 'add or deduct' from has to be filled up if any household migrates. Use the code '8888' as before in the tab and in the tab, manually add to the cluster the household has been joined.

3. Similar to last rounds, in case of absent households, the form will be filled by using absent code 9999. Collecting information about the latrine of absent household is still a must.

4. If any new member (whose name is not in the village list) joins the meeting then enter 7777, give a space and then enter the name in the tab.

5. Cluster leader cannot be changed unless it is a special situation.

6. If any household member uses two latrines then enter information about the hygienic one. If both the latrines are used equally then collect information about the one which is closer to dwelling and contact with the field supervisor.

7. **Rewards will be given based on the total number of (hygienic) latrines in the cluster, not based on the number of households (or their access to hygienic latrines). Tell that to the household members a few times.**

8. Each field supervisor must meet two Health Motivators everyday and oversee 3 meetings.

9. The cluster meeting in which the superivisor is monitoring, has to end with a one-minute summary discussion. This has to be recorded as well.

10. Field supervisor will completely cross check the information about the latine provided by Health Motivator.

11. Information on the hardcopy filled by Health Motivator cannot be sent to the server without the concerned filed supervisor checking it.

12. Audio record of each of the meeting must be kept in the tab and submitted to the concerned field supervisor.

13. In the certificate intervention, there is no need to mention whose signature is going to be put in. Just mention that the certificates will be issued by the Union Parishad.

14. Do not use the word 'leader' in the meeting.

15. Consecutive meetings in the same cluster cannot happen within 18-21 days.

16. The new deadline is [end date] to meet the hygienic latrine criteria. This should be mentioned instead of January 20.

17. Supervisors must monitor three meetings everyday. Cross checks of recordings of the Health Motivators will be done by the supervisors under the guidance of the Project Assistant and Project Associates.

18. Health Motivators and Field Supervisors will be rewarded based on how well the cluster meetings have been conducted according the scripts.

19. Need to finish meetings with the words that "This is the last time I have come to talk about hygienic latrines and the reward/certificate program (if applicable). The next time someone else will come to monitor your progress in achieving hygienic latrine status."

HHID: |___|___|___|___|___|___|

Cluster ID: |___|___|___|___|___|___|___| Village ID: |___|___|___| Village name: ______________ Union ID: |___| Para name: _________________

Interviewer ID: |___|___|___|___| Interviewer's name: ______________________ Supervisor's initial: ______________ Start Time:_______ End time: ________

HH head name: ______________________ Respondent's Name and ID: ___________________________ |____|____| Date: _______________________

(N.B.: If you find anything out of the ordinary at this household, please take notes and contact your supervisor)

Consent

(READ TO RESPONDENT AT THE START OF THE VISIT) **Introduction:** Good morning/afternoon. My name is ___________. I am working with Innovations with Poverty Action, an international research organization. We are currently interviewing households for a study on how people make decisions about sanitation. This study covers 4 unions ([redacted]) in the Tanore Upazilla of Rajshahi.

Procedures: We would like to invite you to participate in our study. If you choose to participate, today we will ask you some questions about the types of sanitation facilities your family uses and your family's interactions with other members of your community. This survey will take approximately 15 minutes of your time. We may also come back once in the next three-six months to conduct a longer follow-up survey that will include questions on the health of your family, the sources of water your family uses and your family's income and assets. The information collected in the follow-up surveys will be used to study how behavior in your community changes over time.

Risks and Benefits: We do not expect there to be any risk to you or your household associated with your participation in this study. There will be no direct benefit to you or your household for your participation in this survey. However, we hope that this research will result in findings that will help in improving access to sanitation facilities in communities throughout Bangladesh.

Confidentiality: All of your responses will remain confidential and will never be shared with anyone besides the researchers involved in this study. Neither your personal information nor any information linking your identity to your responses will ever be made public. Should you feel at any time that you are not comfortable answering a question or that your confidentiality is not assured, for example because someone else may be listening, please let us know.

Voluntary Participation: Participation in this study is completely voluntary. You can refuse to answer the entire survey, or you can tell us when a question makes you uncomfortable and we can skip that question. There is no need to answer any question that you do not wish to answer for any reason. If you like, you can end the interview at any time. There will be no penalty for ending or refusing to participate in the survey.

Questions and Concerns: If you have any questions, comments or concerns, you may contact [redacted]. The relevant principal investigators and their contact information is as follows: 1. [redacted]; 2. [redacted]

I have read (or someone has read to me) and understood the above information. I have had the opportunity to have any questions about this study answered and I agree to participate in this study. **CODE**: 01=Agree, 02=Do not agree **>> STOP SURVEY**, 03=Entire HH absent for extended period of time, 04=Migrated Household, 05=Combined Household, 06=Dwelling destroyed/perished |___|___|

HHID: |___|___|___|___|___|___|

**Section 1**				
1	(ASK and OBSERVE) How many latrines does the household own? (**00** if HH does not own any latrine)			|___|___|
2	(ASK) Does the household have regular access to a latrine? **CODE:** 01=YES, 02= NO **>> skip to Q04**			|___|___|
3	(ASK) Where is the latrine that is used most regularly by the household? **CODE:** 01=In own homestead, 02=Outside own homestead, 03= In neighbor's homestead			|___|___|
**(ASK if household doesn't open defecate): "Could you take me there?"**				
4	(OBSERVE AND ASK) What kind of facility is the latrine most regularly used (primary latrine) by the household? ***(if 01 then fill up Q12 to Q15 and Section 2)***	01. Don't have any latrine/Open defecation	10. Single pit Ring-slab latrine (Offset) with water seal: intact	
		02. Hanging latrine	11. Single pit ring-slab latrine (Offset) with water seal: broken	
		03. Open Pit/hole without slab and lid or cover	12. Single pit Ring-slab latrine (Offset) with flip/ pollythene	
		04. Pit latrine with slab but without lid or cover	13. Double pit Ring-slab latrine (Offset) with water seal: intact	
		05. Pit latrine with cover	14. Double pit Ring-slab latrine (Offset) with water seal: broken	
		06. Modern pit latrine with vent pipe	15. Double pit Ring-slab latrine (Offset) with flip/ pollythene	
		07. Ring-slab latrine (direct) with water seal: intact	16. Eco Latrine	
		08. Ring-slab latrine (direct) with water seal: broken/none	17. Sanitary latrine with septic tank	
		09. Ring-slab latrine (direct) with flip/ polythene	|___|___|	
**For Q5 to Q13, ASK**				
5	What is the ownership status of the primary latrine? **CODE:** 01=Toilet jointly owned with another household, 02=private toilet solely owned by household, 03=community toilet **>>Q12**, 04=owned by others/neighbor**>>Q08**			|___|___|
6	How much money was spent on the latrine (in total)? (in Tk.) **CODE:** 99=Don't remember/Don't know<br>**NOTE:** Ask about material/labor/transport cost separately before writing the total amount. If the cost is **99** taka, round it to **100** taka.			Total tk. _______________
7	a. Did you borrow any money to install the latrine? **CODE**: 01=Yes, 02=No**>>Q08**, 99= Don't Know**>>Q08**	|___|___|	b) If yes, from whom? (Code below)	|___|___|
**Borrowing Code:** 01=Bank, 02=NGO, 03=Cooperative/MFI, 04=Private (organization), 05= Private (Individual), 06=Bought on credit, 99=Don't Know				
8	How many households and individuals share the use of this latrine facility?	|___|___| households	|___|___| individuals	
9	Identify the households who own the latrine ***(Use "99" if you cannot find the HH and fill up Section 03. If outside the survey area, use "55")***			
	**a.** |___|___|___|___|___|___| **b.** |___|___|___|___|___|___|	**c.** |___|___|___|___|___|___|	**d.** |___|___|___|___|___|___|	
10	a) How long has your primary latrine been in place? **CODE:** 01=0 - 6 months; 02 =6 months - 1 year; 03=1 - 2 years; 04 = 2 - 3 years; 05= 3-5 years; 06=more than 5 years	|___|___|	b) ***If built since February, 2012*** How many months ago was this latrine built? **CODE:** 99=can't remember/don't know, 77=built before February, 2012	|___|___|
11	a. Number of rings (USE "00" IF NO RINGS) (**CODE**: 99=Don't know) (Use 66 only for septic tanks)	|___|___|	b. How deep is the pit? (***Record answer in fet***) **CODE:** 99=Don't know	|___|___|

1

HHID: |___|___|___|___|___|___|

<table>
<tr><td>12</td><td colspan="4">Where do the HH members usually wash their hands (henceforth, to be termed 'the hand-washing station') after going to the toilet?
CODE: 01 = Inside toilet facility, 02=less than 3ft from toilet facility, 03=3-9ft of toilet facility, 04=more than 9ft from toilet facility, 05=No specific place **>>Q16**, 06=Do not wash**>>Q16**</td><td>|___|___|</td></tr>
<tr><td>13</td><td colspan="4">Do HH members wash hands after returning from toilet? **CODE:** 01=Yes, with soap and water, 02= Yes, with only water, 03=Yes, With ash and water, 04= Yes, With clay and water (multiple responses allowed)</td><td>|___|___|
|___|___|</td></tr>
<tr><td colspan="6">**For Q14 to Q37, <u>OBSERVE</u>**</td></tr>
<tr><td>14</td><td colspan="4">Is water available at the hand washing station? **CODE:**1=Yes, adequate water is available, 2=No, adequate water is not available, 3=No water available</td><td>|___|___|</td></tr>
<tr><td>15</td><td colspan="4">Is soap and/or clay/ash available at the hand washing station? **CODE:** 01=Bar soap, 02=Powder/detergent, 03=Liquid soap, 04=Clay/ash, 05=No soap/clay/ash observed (multiple responses allowed)</td><td>|___|___|
|___|___|</td></tr>
<tr><td>16</td><td colspan="3">a. Have you (the interviewer) observed a HH member running to clean a latrine because of your presence or you suspect so? **CODE:** 1=Yes, 2=No ***(Take note if necessary)***</td><td colspan="2">|___|___| ______________________________</td></tr>
<tr><td>17</td><td>What materials were used to construct the superstructures?</td><td colspan="2">a. walls? |___|___| _______________</td><td colspan="2">b. roofs? |___|___|____________</td></tr>
<tr><td colspan="6">**CODE:** 00=none, 01=mud, 02=leaves/stick/straw, 03=bamboo thatch, 04=plastic sheets/polythene, 05=CI sheets/tin, 06=wood, 07=clay tiles, 08=bricks, 09=RC/cement/concrete, 10=other (specify)</td></tr>
<tr><td>18</td><td colspan="4">Is the latrine pipe linked to any ditch/canal/pond etc.? **CODE:** 1=Yes, 2=No</td><td>|___|___|</td></tr>
<tr><td>19</td><td>**a)** (OBSERVE) Leakage of the latrine pipe, Y junction, pit or the tank? Code: 01=Major, 02=Minor, 03=No leak**>>Q21**</td><td>|___|___|</td><td colspan="2">**b)** Leakage in which component? Code: 01=Pipe, 02=Pit, 03=Tank, 04=Y-junction ***(Can choose several)***</td><td>|___|___|, |___|___|, |___|___|</td></tr>
<tr><td>20</td><td colspan="4">***If pit thought to be leaking*** what are the signs of leaking? **CODE:** 01=Wet soil around pit, 02=Water-logged area outside/around pit, 03=Floating feces outside pit, 04=Some odour, 05=Strong odour *(multiple responses allowed)*</td><td>|___|___|, |___|___|, |___|___|</td></tr>
<tr><td>21</td><td colspan="4">***If offset*** what is the current condition of delivery pipe/Y junction? **CODE**: 01=Fully Intact, 02=Partially Broken, 03=Completely Broken, 04= No Delivery Pipe, 66=Not an offset pit latrine**>>Q23**</td><td>|___|___|</td></tr>
<tr><td>22</td><td colspan="4">***If offset*** What is the current condition of pit cover? **CODE**: 01=Fully Intact, 02=Partially Broken, 03=Completely Broken, 04= No Pit Cover</td><td>|___|___|</td></tr>
<tr><td>23</td><td colspan="4">Any broken ring? **CODE**: 01=Yes, 02=No, 66=No ring/Septic tank</td><td>|___|___|</td></tr>
<tr><td>24</td><td colspan="4">Type of the latrine slab **CODE:** 00=No slab**>>Q28**, 01=Concrete/cement, 02=Plastic, 03=Bamboo, 04=Brick, 05=Earthen, 06=Others (mention)</td><td>|___|___|</td></tr>
<tr><td>25</td><td colspan="4">What is the current condition of latrine slab? **CODE**: 01=Fully Intact, 02=Partially Broken, 03=Completely Broken</td><td>|___|___|</td></tr>
<tr><td>26</td><td colspan="4">**a.** Type of pan **CODE:** 1=Ceramic commode, 02=Ceramic pan, 03=Plastic pan, 04=Concrete/Cement pan, 05=Tin, 06= Bamboo top/covered with polythene, 07=No pan**>>Q08**</td><td>|___|___|</td></tr>
<tr><td>26</td><td colspan="4">**b.** What is the current condition of the pan? **CODE**: 01=Fully Intact, 02=Partially Broken, 03=Completely Broken</td><td>|___|___|</td></tr>
<tr><td>27</td><td colspan="4">What is the current condition of the water seal? **CODE**: 01=Fully Intact, 02=Partially Broken, 03=Completely Broken, 04=No water seal</td><td>|___|___|</td></tr>
<tr><td>28</td><td colspan="4">Presence of Vent pipe on the pit, inside the latrine or outside, condition and net? **CODE:** 01=Yes, good condition, with net, 02=Yes, good condition, without net, 03=Yes, broken, 04=No vent pipe</td><td>|___|___|</td></tr>
<tr><td>29</td><td colspan="4">Has the 'slab and pan' (for direct pit) or pit cover (for off-set) been placed properly on top of pit so that flies cannot enter/exit? **CODE**: 1=Yes,</td><td>|___|___|</td></tr>
</table>

HHID: |___|___|___|___|___|___|

<table>
<tr><td></td><td colspan="5">2=No, 66=No Slab/pan/pit cover</td><td></td></tr>
<tr><td rowspan="2">30</td><td colspan="2">a) Any evidence of fecal matter on the latrine floor? CODE: 00=None, 01=Trace amounts only, 02=Lumps of Feces</td><td>|___|___|</td><td colspan="2" rowspan="2">b) Any evidence of fecal matter on the pan? CODE: 00=None, 01=Trace amounts only, 02=Lumps of Feces, 66=No pan>>Q31</td><td rowspan="2">|___|___|</td></tr>
<tr><td colspan="2">c) Any fecal matter floating in the gooseneck/water-seal? CODE: 00=None, 01=Trace amounts only, 02=Lumps of Feces, 66=No water-seal</td><td>|___|___|</td></tr>
<tr><td>31</td><td colspan="5">Is there a bad smell in and around the latrine? CODE: 01=Strong bad smell, 02=Some bad smell, 03=No bad smell</td><td>|___|___|</td></tr>
<tr><td>32</td><td colspan="5">Is there any visibility of flies or insects inside or around the latrine? CODE: 01=Yes, 02=No</td><td>|___|___|</td></tr>
<tr><td rowspan="4">33</td><td>a) Slippers outside or inside latrine? CODE: 1=Yes, 2=No</td><td>|___|___|</td><td>b) Wet floor? CODE: 01=Yes, 02=No</td><td>|___|___|</td><td>c) Dis-coloration of pan (e.g. yellow/ green) CODE: 01=Yes, 02=No, 66=No pan>>e</td><td>|___|___|</td></tr>
<tr><td colspan="4">d) Do you observe the following in the pan (that denotes lack of use) (Multiple response possible) CODE: 01= Leaves, 02= dirt, 03=spider webs, 04=Nothing</td><td colspan="2">|___|___| , |___|___| , |___|___|</td></tr>
<tr><td>e) Water container in/near the latrine? CODE: 01=Yes, 02=No</td><td>|___|___|</td><td>f) Cleaning agents inside the latrine? CODE: 01=Broom/brush, 02=Harpik/Bleaching powder/detergent, 03=Ash, 04=No cleaning agent</td><td>|___|___|</td><td>g) Is there evidence that this latrine is used for storage? CODE: 01=Yes, 02=No</td><td>|___|___|</td></tr>
<tr><td colspan="5">h) Does the path to the toilet suggest regular use (is it clear, well-worn, etc)? CODE: 01 = Yes; 02 = No</td><td>|___|___|</td></tr>
<tr><td>34</td><td colspan="5">Level of the latrine pan: CODE: 01=Above yard height, 02=At yard height, 03=Somewhat lower than the yard, 04=Much lower than the yard</td><td>|___|___|</td></tr>
<tr><td>35</td><td colspan="5">How many lumps of feces have you observed in the compound? CODE: 01=No lump of feces, 02=1-5 lumps, 03=6-10 lumps, 04=More than 10 lumps</td><td>|___|___|</td></tr>
<tr><td>36</td><td colspan="5">Physical/Travel distance of latrine from main living room CODE: 00=Inside home compound, 01=01-10 ft, 02=11-20 ft, 03=21-30 ft, 04=More than 30 ft</td><td>|___|___|</td></tr>
<tr><td rowspan="2">37</td><td colspan="5">a. (If the source of drinking water is TW or Dug well) Physical/Travel distance of latrine from the TW/ DW (Use next question's code)</td><td>|___|___|</td></tr>
<tr><td colspan="5">b. Physical/Travel distance between the latrine and the source of water used in the latrine: Code: 00=Attached to the room, 01=01-10 ft, 02=11-20 ft, 03=21-30 ft, 04=More than 30 ft</td><td>|___|___|</td></tr>
<tr><td colspan="7">Section 02 (For Q38 to Q46 <u>ASK</u>)</td></tr>
<tr><td>38</td><td colspan="5">Since November 01, 2013, (the month of Karttik in Bangla calendar) have you installed a complete latrine or any latrine parts? CODE: 01=Complete latrine>>Q40, 02=Latrine Parts, 03=Neither >> Q42</td><td>|___|___|</td></tr>
<tr><td>39</td><td colspan="3">If you have installed any parts, list all such latrine parts (Use CODE B)</td><td colspan="3">|___|___| , |___|___| , |___|___| , |___|___| , |___|___|</td></tr>
<tr><td colspan="7">CODE B: 01=Water seal/goose-neck/syphon, 02=Slab/Pit Cover, 03=Pit Cover with pan, 04=Pan, 05=Delivery pipe/Y Junction, 06=Ventilation pipe, 07=Rings, 08=Mud/bricks/cement/sand, 09=Door, 10=Other (detail in note)</td></tr>
<tr><td>40</td><td colspan="5">Is this (for) the same latrine you have mentioned as your primary latrine? CODE: 01=Yes, 02=No</td><td>|___|___|</td></tr>
<tr><td>41</td><td colspan="5">If you have installed a complete latrine or any parts Since November 01, 2013 what was the amount spent in total (In taka) (Including transportation, labor etc.)</td><td>|___|___|___|___|___|</td></tr>
</table>

HHID: |___|___|___|___|___|___|

42	Since November 01, 2013 ***(prompt 'in the last three months' or 'since mid-Karttik' of Bangla calendar)*** has anyone from your community assisted you ('with advice-information/material/labor/cash/transport') to make your latrine hygienic? **CODE:** 01=Yes, 02=No**>>*Q44***
43	Since November 01, 2013 if you have received any assistance from your community to make your latrine hygienic, list the Households and the kind of assistance. **(If cannot find HHID, use "99" and fill up section 03. If HH is outside the survey area, use "55")**

a. HHID	b. Relationship with HH head (**Code:** 01=parents; 02=siblings; 03=children;04=near relatives;05=neighbor/community member; 06=other)	c. Assistance (**Code**: 01=Material; 02=Cash Loan **>>e**; 03=Cash help**>>e**; 04=Labor support**>>f**; 05=Advice or Information**>>g,h&i**; 06=Transport; 07=Other - Take Note)	d. What kind of material? (**Code B**);	e. Amount (in Tk.)	f. How many labor hours?	g. Frequency of mention/ discussion on latrines	h. In private or public gathering (01=private, 02=public, 03=both)	i. Did you feel pressurized? (**Code**: ***If No***, use 00; ***If Yes***, use pressure scale 01-05; 01=least and 05=highest pressure)
**a**	**b**	**c**	**d**	**e**	**f**	**g**	**h**	**i**

44

a. Since November 01, 2013, have you received any assistance ('with material/cash/advice') from the government/UP or any NGO to make your latrine hygienic? **CODE:** 1=Yes, 2=No**>>Q45**

b. NGO/Government/UP **Code:** 01=Government/UP; 02=BRAC; 03=World Vision; 04= IPA/VERC; 05=Other NGOs	c. Assistance Code (**Code**: 01=Material; 02=Cash Loan **>>e**; 03=Cash help**>>e**; 04=Labor support**>>f**; 05=Advice or Information**;** 06=Transport; 07=Other - Take Note)	d. What kind of material? (**Code** A); put commas in between each item	e. Amount (in Tk.)	f. Labor hours	Note

CODE B: 01=Water seal/goose-neck/syphon, 02=Slab/Pit Cover, 03=Pit Cover with pan, 04=Delivery pipe, 05=Ventilation pipe, 06=Rings, 07=Mud/bricks/cement/sand, 08=Transportation, 09=Labor, 10=Door, 11=Superstructure (e.g. tin), 12=Other (detail in note)

HHID: |___|___|___|___|___|___|

<table>
<tr><td>45</td><td colspan="3">Did you attend or know of any meeting sessions that were conducted in your village to promote hygienic sanitation between November 01, 2013 to February 05, 2014? CODE: 01= Yes, attended, 02=Knows, but did not attend, 03=Neither >>Q47 >>END</td><td>|___|___|</td></tr>
<tr><td rowspan="9">46</td><td colspan="4">(Even if HH didn't attend) What information was covered in these meetings? (Do not read out the options aloud) CODE: 1=Yes; 2=No</td></tr>
<tr><td>a. Private Pledge</td><td>|___|___|</td><td>i. Reward (Money)</td><td>|___|___|</td></tr>
<tr><td>b. Public Pledge</td><td>|___|___|</td><td>j. Reward (Certificate)</td><td>|___|___|</td></tr>
<tr><td>c. Deadline (regarding hygienic latrine attainment)</td><td>|___|___|</td><td>k. Target (percentage or no. of households)</td><td>|___|___|</td></tr>
<tr><td>d. Disadvantages of Open Defecation</td><td>|___|___|</td><td>l. Importance of washing hands</td><td>|___|___|</td></tr>
<tr><td>e. Importance of proper disposal of feces</td><td>|___|___|</td><td>m. Installing a hygienic latrine</td><td>|___|___|</td></tr>
<tr><td>f. Importance of hygienic sanitary habits</td><td>|___|___|</td><td>n. Assessing quality of latrine parts</td><td>|___|___|</td></tr>
<tr><td>g. Importance of using hygienic latrine</td><td>|___|___|</td><td>o. Places where quality latrines parts are available</td><td>|___|___|</td></tr>
<tr><td>h. Maintaining hygienic latrine</td><td>|___|___|</td><td>p. Others (Specify): ___________</td><td>|___|___|</td></tr>
<tr><td>47</td><td colspan="2">a. Did any of your neighbor build a latrine since November 01, 2013? CODE: 01=Yes; 02=No >>Q48 |___|___|</td><td>b. How many households?</td><td>|___|___|</td></tr>
<tr><td rowspan="9">46</td><td colspan="4">(Even if HH didn't attend) What information was covered was covered in these meetings? (READ OUT the options) CODE: 1=Yes; 2=No</td></tr>
<tr><td>a. Private Pledge</td><td>|___|___|</td><td>i. Reward (Money)</td><td>|___|___|</td></tr>
<tr><td>b. Public Pledge</td><td>|___|___|</td><td>j. Reward (Certificate)</td><td>|___|___|</td></tr>
<tr><td>c. Deadline (regarding hygienic latrine attainment)</td><td>|___|___|</td><td>k. Target (percentage or no. of households)</td><td>|___|___|</td></tr>
<tr><td>d. Disadvantages of Open Defecation</td><td>|___|___|</td><td>l. Importance of washing hands</td><td>|___|___|</td></tr>
<tr><td>e. Importance of proper disposal of faeces</td><td>|___|___|</td><td>m. Installing a hygienic latrine</td><td>|___|___|</td></tr>
<tr><td>f. Importance of hygienic sanitary habits</td><td>|___|___|</td><td>n. Assessing quality of latrine parts</td><td>|___|___|</td></tr>
<tr><td>g. Importance of using hygienic latrine</td><td>|___|___|</td><td>o. Places where quality latrines parts are available</td><td>|___|___|</td></tr>
<tr><td>h. Maintaining hygienic latrine</td><td>|___|___|</td><td>p. Others (Specify): ___________</td><td>|___|___|</td></tr>
</table>

SM6 Cost-Effectiveness Calculation

Public commitment

The cost per household for the common intervention (monthly group meetings for three consecutive months with a Health Motivator to encourage investment in and maintenance and use of hygienic latrines) was approximately USD 2. The marginal (implementation) cost of adding public commitment to the common intervention was negligible since no additional personnel/component was necessary. Since the average group size was 15.6 households, the group-level intervention cost was USD 31.2.

Regression results show that groups in the public commitment treatment had 4.5 percentage point more hygienic latrines in the short term and 5.7 percentage point more hygienic latrines in the medium term, compared to meetings only/common intervention (Table 3). On the other hand, compared to the pure control groups, the meetings only groups had approximately 1.5 percentage point fewer hygienic latrines in the short term and 2.5 pp fewer latrines in the medium term (Table A1). This translates into 1.09 (0.07*15.6) more hygienic latrines per group that can be attributed to the public commitment treatment (compared to pure control) in the short term. Dividing the group-level cost by the additional hygienic latrines in the group due to the treatment, gives a value of USD 28.6.

Similarly, in the medium term, 1.28 (0.082*15.6) more hygienic latrines per group that can be attributed to the public commitment treatment (compared to pure control). Dividing the group-level cost by the additional hygienic latrines in the group due to the treatment, gives a value of USD 24.4.

This is a simple back-of-the-envelope calculation, of course. We are assuming that the treatment effect is there only for the duration of the study, i.e., it drops to zero at the end of the study period. Moreover, we are looking only at the program costs, i.e., we are not including the cost to the household of making the improvements.

Monetary Reward

As mentioned earlier, the cost per household for the common intervention was USD 2. No additional cost was required for implementing the monetary reward treatment, except for the conditional reward which was calculated based on individual and group-level hygienic latrine ownership. We calculate that a total of USD 8,282 was disbursed among the monetary reward groups at the end of the intervention. These groups had a total of 3,612 households (Table 1), which brought the per household cost to USD 2.29. Together with the common intervention cost, the total per household cost for the monetary reward treatment was USD 4.29. The group-level cost was, therefore, USD 66.92 (15.6*4.29).

Regression results show that groups in the monetary reward treatment had 7.8 percentage points more hygienic latrines in the short term compared to meetings only/common intervention (Table 3). On the other hand, compared to the pure control groups, the meetings only groups had approximately 1.5 percentage point fewer hygienic latrines in the short term

(Table A1). This translates into 1.45 (0.093*15.6) more hygienic latrines per group that can be attributed to the monetary reward treatment (compared to pure control) in the short term. Dividing the group-level cost by the additional hygienic latrines in the group due to the treatment, gives a value of USD 46.15 (66.92/1.45).

We do not attempt to calculate the cost effectiveness of the monetary reward in the medium term, since the effect size (from the short term) almost completely dissipates and does not remain statistically significant (even at the 10 percent level).

Supplementary Materials References

Reed, B. (2014). "Pour-flush latrines." WEDC Guide No. 26. Loughborogh University. https://hdl.handle.net/2134/30994.

Tilley, E., L. Ulrich, C. Luthi, P. Reymond, and C. Zurbrugg (2014). *Compendium of Sanitation Systems and Technologies: 2nd Revised Edition*. Duebendorf, Switzerland: Swiss Federal Institute of Aquatic Science and Technology (Eawag). https://sswm.info/node/5587.
